# Dietary supplement consumption among active individuals in Saudi Arabia

**DOI:** 10.1371/journal.pone.0351208

**Published:** 2026-06-22

**Authors:** Abdulrahman A. Alsayegh, Abeer S. Alzaben, Yahya M. Abu Haddash, Abdulrahman M. Busayli, Rama M. Chandika, Munirah N. Alsuhaibani, Sara A. Albishi, Mahitab A. Hanbazaza, Omar I. Abuzaid, Majed M. Alkhalaf

**Affiliations:** 1 Department of Clinical Nutrition, College of Nursing and Health Sciences, Jazan University, Jazan, Saudi Arabia; 2 Department of Health Sciences, College of Health and Rehabilitation Sciences, Princess Nourah bint Abdulrahman University, Riyadh, Saudi Arabia; 3 Saudi Food and Drug Authority, Riyadh, Kingdom of Saudi Arabia; 4 Clinical Nutrition Department, College of Applied Medical Sciences, University of Hafr Al Batin, Hafr Al-Batin, Saudi Arabia; 5 Department of Food and Nutrition, Faculty of Human Sciences and Design, King Abdulaziz University, Jeddah, Saudi Arabia; 6 Department of Clinical Nutrition, College of Applied Medical Sciences, Imam Abdulrahman bin Faisal University, Dammam, Kingdom of Saudi Arabia; 7 Executive Directorate of Public Health Policies and Regulations at Saudi Public Health Authority, Riyadh, Saudi Arabia; University of Luzon, PHILIPPINES

## Abstract

**Background:**

Despite the increasing use of dietary supplements in Saudi Arabia, accurate information on their consumption patterns and predictors is essential for effective policy planning.

**Objective:**

This study aimed to estimate the prevalence of dietary supplement consumption, assess perceptions regarding their health effects, and identify demographic, health-related, and knowledge- and practice-based factors influencing their use among active individuals in Saudi Arabia.

**Methods:**

This cross-sectional study identified predictors of dietary supplement consumption using a Likert-scale questionnaire adapted from validated local and international tools with minor modifications. Structural equation modeling was conducted by defining multiple sets of regression equations. The analyses were performed using R software (Version 4.1.2) with the lavaan and semPlot packages. A two-tailed p-value < 0.05 was considered statistically significant.

**Results:**

A total of 3,800 active individuals from 13 Saudi Arabian regions participated in this study. The prevalence of dietary supplement use among active individuals was 63.82%. Health characteristics and knowledge and practice domains showed a highly significant influence on supplement consumption, whereas the demographic domain did not show a significant association in the structural equation model, although several demographic variables were significantly associated with supplement use in bivariate analysis.

**Conclusion:**

The prevalence of dietary supplement use among physically active individuals in Saudi Arabia exceeded the prevalence rates reported in several international populations, with more than three out of every five participants indicating use of dietary supplements.

## Introduction

The Dietary Supplement Health and Education Act of 1994 defines dietary supplements as products containing one or more nutritional components [1]. These include vitamins, minerals, herbs, meal replacements, sports nutrition products, natural food supplements, and other substances used to complement dietary intake [2]. Globally, dietary supplements have become widely used, with up to 60% of athletes reporting their use. The most commonly consumed supplements include multivitamin/multimineral preparations, amino acids or protein products, creatine, and sports drinks [3]. The primary motivations for dietary supplement use are to enhance general health and well-being, particularly when access to nutritious food is limited, and to reduce the risk of specific diseases. Additionally, athletes often use dietary supplements to increase energy levels and pain tolerance, maintain strength and performance, support immune function, and address nutritional deficiencies [4]. Several studies conducted in Saudi Arabia have reported that the prevalence of dietary supplement use among active individuals ranges from 30% to 90% [5–11]. More than 93.3% of Saudi professional athletes consume dietary supplements [12]. Furthermore, approximately half of gym members aged 18–25 years in Riyadh use dietary supplements such as protein powder, vitamins, sports drinks, high-carbohydrate bars, fat burners, and weight gain products [13]. Men are more likely than women to use nutritional supplements [5]. However, recent studies have also reported high usage rates among women attending fitness centers, ranging from 46% to 69% [14,15]. Notably, over 50% of participants believed that high dietary supplement intake was safe, suggesting limited awareness of the potential adverse effects associated with excessive use [8]. Similarly, a recent study conducted in western Saudi Arabia reported that although 82.5% of participants were aware of dietary supplements, only 41% knew the correct dosages, and nearly one-third were uncertain about their safety [16]. University students in health sciences used dietary supplements more frequently than students in other disciplines, despite their limited awareness of these products [17]. Only 25.8% of elite athletes were aware of the harmful consequences of irrational dietary supplement use. Moreover, the same study indicated that individuals had limited access to reliable sources of information regarding dietary supplements [7]. Another study found that approximately 42% of participants from Riyadh, Saudi Arabia, believed dietary supplements were generally safe to consume [10]. Several studies from Saudi Arabia have emphasized the need to raise awareness regarding dietary supplement use. However, research remains limited at the national level in assessing the predictors of dietary supplement consumption, which are essential for providing empirical evidence to guide effective policies and develop targeted interventions. Therefore, this study aimed to estimate the prevalence of dietary supplement use, assess perceptions regarding its health effects, and identify factors such as demographic, health, and knowledge-and practice-related variables that influence dietary supplement consumption among active individuals in Saudi Arabia.

## Materials and methods

### Study design and population

A cross-sectional study was conducted among active individuals in the Kingdom of Saudi Arabia between February 1, 2023, and February 1, 2024. Following a critical review of validated local and international tools assessing dietary supplement use, a questionnaire was adapted with minor modifications to ensure cultural relevance [12,18]. The questionnaire was translated into Arabic through a standardized forward–backward translation process performed by independent bilingual translators (Questionnaire_English_Version). Face validity was established through pilot testing with 25 gym users who were asked to provide feedback on the clarity, wording, and comprehension of each item. Minor linguistic adjustments were made based on participant feedback, and the obtained Cronbach’s alpha value of 0.81 indicated acceptable internal consistency. Content validity was evaluated by experts from the Saudi National Nutrition Committee. Reliability was assessed using the test–retest method, in which a sample of 25 gym users completed the questionnaire twice within a two-week interval. The two sets of responses were compared using a correlation coefficient, which demonstrated a statistically significant stability coefficient, indicating good test–retest reliability. A factor loading threshold greater than 0.2, a comparative fit index of 0.901, and a root mean square error of approximation of 0.072 obtained through confirmatory factor analysis supported the construct validity of the questionnaire. The final version of the questionnaire was subsequently approved by the National Nutrition Committee of the Saudi Food and Drug Authority. The questionnaire was administered in Arabic and consisted of 19 questions divided into three sections. The first section included nine questions aimed at collecting self-reported sociodemographic information from participants. These variables included gender (male or female), age (<25 years or ≥25 years), nationality (Saudi or non-Saudi), marital status (single, married, or divorced/widowed), region (central, eastern, western, northern, or southern), employment status (student, employee, unemployed, or retired/self-employed), educational level (pre–high school/uneducated, high school or equivalent, bachelor’s degree, or postgraduate certificate), income level(SAR) (<2,000, 2,000– < 5,000, 5,000– < 7,000, 7,000– < 10,000, or ≥10,000), and smoking status (yes or no). The second section contained five questions designed to collect self-reported health information from the participants. These variables included the following questions: How do you perceive your general health? (weak, fair, good, or excellent); Have you ever enrolled in a weight control program with a dietitian? (yes or no); How do you perceive your general fitness level? (weak, fair, good, or excellent); How many minutes per week do you engage in physical activity? (<150 minutes per week, ≥ 150 minutes per week, or do not know); and Do you have professional experience in sports? (yes or no). The final section comprised five questions designed to assess participants’ knowledge and practices regarding dietary supplement use. These questions were as follows: How often do you use dietary supplements? (daily, 4–6 times per week, 2–3 times per week, once per week, once per month, or once per month or less); Which of the following best describes your knowledge of dietary supplement ingredients? (all ingredients, most ingredients, some ingredients, or none); Do any of the supplements you use contain caffeine? (yes, do not know, or no); How confident are you in the effectiveness and benefits of the dietary supplements you use? (very confident, confident, somewhat confident, or not confident at all); and During the past three months, on average, how much money did you spend per month on dietary supplements (SAR)? (<500, 500– < 1,000, 1,000– < 1,500, or ≥1,500). The questionnaire was distributed online among active individuals in collaboration with gym administrators nationwide. All participants were aged ≥18 years, resided in Saudi Arabia, and engaged in ≥150 minutes of moderate-intensity or ≥75 minutes of vigorous-intensity aerobic physical activity per week. Individuals with acute diseases requiring medication, pregnant or lactating women, and those unable to read Arabic were excluded from the study.

### Active individuals

In this study, an active individual was defined as a person who engages in ≥150 minutes per week of moderate-intensity or ≥75 minutes per week of vigorous-intensity aerobic physical activity. Moderate-intensity activities included brisk walking (≥3 miles per hour but not race walking), water aerobics, bicycling at <10 miles per hour on level terrain without hills, doubles tennis, ballroom dancing, and general gardening. Vigorous-intensity activities included race walking, jogging or running, swimming laps, singles tennis, aerobic dancing, bicycling at ≥10 miles per hour including uphill paths, rope jumping, heavy gardening (continuous digging or hoeing), and hiking uphill or carrying a heavy backpack [19,20].

### Dietary supplements

The Dietary Supplement Health and Education Act of 1994 defines dietary supplements as products containing one or more nutritional components [1,2]. These include vitamins, minerals, herbs, meal replacements, sports nutrition products, natural food supplements, and other substances used to complement dietary intake. Based on the frequency of use, dietary supplements were categorized into ten main groups: proteins (e.g., amino acids, protein bars, protein powders, creatine, and protein-enriched milk); vitamins and minerals (e.g., multivitamins, vitamin A, vitamin B complex, vitamin C, calcium, selenium, zinc, and iron); sports drinks; herbs; weight gain supplements; high-carbohydrate bars; fat-loss supplements; omega-3; collagen; and coffee.

### Sample size and sampling frame

Saudi Arabia, officially known as the Kingdom of Saudi Arabia, is a country located in West Asia. It is divided into 13 administrative regions and has a population exceeding 32 million [21]. The required sample size for each administrative region was calculated based on the proportion of individuals aged ≥18 years who engaged in sports activities for ≥150 minutes per week [22], using a Z-value of 1.96, a 95% confidence interval (CI), an absolute precision of 5%, and a 20% non-response rate. After aggregating the required samples across all administrative regions, a total of 3,788 active individuals were determined as the necessary sample size for this study (S1 Table). A total of 90 gym centers were visited across the 13 provinces of Saudi Arabia, assuming an average of 50 gym users per center. Based on this assumption, 4,500 eligible participants were initially approached. Gym locations were identified using Google Maps and local business listings, as the study was restricted to gym users. Lists of eligible participants, including WhatsApp contact information, were compiled by gym volunteers or administrators. Participants were then enrolled using a systematic random sampling method to ensure national representativeness of gym users. Every kth participant (k = total eligible participants in the selected gym ÷ 50) was systematically selected until 50 participants were reached from each gym center. If a participant declined to participate, the subsequent individual on the list was included. The online questionnaire was distributed via WhatsApp with assistance from volunteers or gym administrators. In total, 3,800 gym users completed the survey, yielding an 84% response rate.

### Statistical analysis

Qualitative variables were presented as frequencies and percentages. Path analysis was conducted to examine the complex relationships between the observed variable—dietary supplement use—and the latent variables, including demographic, health, and knowledge and practice domains. Structural equation modeling (SEM) was performed by defining multiple sets of regression equations. A root mean square error of approximation <0.08, a comparative fit index ≥0.90, and a chi-squared (χ2) statistic were used to evaluate model fit. Data were coded, validated, and analyzed using IBM SPSS Statistics software (version 27.0; IBM Corp., Chicago, IL, USA). SEM plots were generated using R software (version 4.1.2) with the lavaan and semPlot packages. A two-tailed p-value <0.05 was considered statistically significant.

### Ethical consideration

All ethical procedures adhered to the principles outlined in the Declaration of Helsinki and the Saudi Bioethics standards. Participants were informed at the beginning of the Google Form questionnaire through a structured narrative describing the study’s purpose, their right to withdraw at any time without penalty, and the confidentiality of their responses. Written informed consent was digitally obtained prior to participation. Ethical approval for this study was granted by the Standing Committee for Scientific Research of Princess Nourah bint Abdulrahman University (IRB Log Number: 22-1191), Riyadh, Saudi Arabia.

## Results

A total of 3,800 active individuals (957 men and 2,843 women) participated in the study. More than half of the participants (2,057, 54.13%) were aged <25 years. The majority (3,547, 93.34%) were Saudi nationals, and over three-quarters (2,881, 75.82%) were single or unmarried. The largest proportion of participants (1,177, 30.97%) were from the southern region. Nearly half (1,860, 48.95%) were students, and more than half (2,369, 62.34%) held a bachelor’s degree. Almost half (1,868, 49.16%) reported a monthly income (SAR) <2,000. Most participants (3,496, 92%) were non-smokers (S2 Table). Approximately half (50.05%) of the participants rated their general health as excellent, while nearly half (47.18%) rated their fitness level as fair. More than one-fourth (26.55%) reported enrolling in weight control programs with dietitians, and over half (59.89%) engaged in ≥150 minutes of physical activity per week. Additionally, 10.13% were professional athletes (S3 Table). The association between background characteristics and dietary supplement consumption among active individuals is presented in Table 1. A significantly higher proportion of men (69.80%) used dietary supplements compared with women (61.80%) (p < 0.01). Similarly, single or unmarried participants (66.57%) consumed more supplements than married (55.65%) and divorced/widowed (50.00%) participants, and this difference was also highly significant (p < 0.01). Active individuals from the central region of Saudi Arabia (69.04%) consumed more dietary supplements than those from the eastern (63.27%), western (63.44%), northern (64.66%), and southern (59.81%) regions, and this difference was highly significant (p < 0.01). Employed participants (72.53%) reported greater supplement use than students (62.42%), unemployed individuals (57.88%), and retired or self-employed participants (49.83%) (p < 0.01). Participants with postgraduate education (79.71%) consumed more dietary supplements than those with pre–high school or no education (35.51%), high school or equivalent education (60.30%), and bachelor’s degrees (66.48%). Participants (77.31%) with a monthly income (SAR) of 5,000– < 7,000 consumed more dietary supplements than those in other income brackets, which was highly significant (p < 0.01). Those who spent (SAR) 500– < 1,000 monthly on dietary supplements (76.60%) reported higher consumption levels than participants in other expenditure groups (p < 0.01). Smokers (69.41%) consumed more dietary supplements than non-smokers (63.33%), and this difference was significant (p < 0.05). However, age and nationality did not show significant associations with supplement use.

**Table 1:** Association between background characteristics and dietary supplement consumption among active individuals.

Variable	Categories	Do you consume nutritional supplements?			^§^P-value
		Yes (%)	No (%)	Total (%)	
		2425 (63.82%)	1375 (36.18%)	3800 (100%)	
Gender	Male	668 (69.80%)	289 (30.20%)	957 (100%)	0.001^*^
	Female	1757 (61.80%)	1086 (38.20%)	2843 (100%)	
Age	<25	1308 (63.59%)	749 (36.41%)	2057 (100%)	0.78
	≥25	1117 (64.08%)	626 (35.92%)	1743 (100%)	
Nationality	Saudi	2271 (64.03%)	1276 (35.97%)	3547 (100%)	0.35
	Non-Saudi	154 (60.87%)	99 (39.13%)	253 (100%)	
Marital Status	Single/Unmarried	1918 (66.57%)	963 (33.43%)	2881 (100%)	0.001^*^
	Married	468 (55.65%)	373 (44.35%)	841 (100%)	
	Divorced/Widowed	39 (50.00%)	39 (50.00%)	78 (100%)	
Region	Central	582 (69.04%)	261 (30.96%)	843 (100%)	0.001^*^
	Eastern	205 (63.27%)	119 (36.73%)	324 (100%)	
	Western	387 (63.44%)	223 (36.56%)	610 (100%)	
	Northern	547 (64.66%)	299 (35.34%)	846 (100%)	
	Southern	704 (59.81%)	473 (40.19%)	1177 (100%)	
Employment status	Student	1161 (62.42%)	699 (37.58%)	1860 (100%)	0.001^*^
	Employee	816 (72.53%)	309 (27.47%)	1125 (100%)	
	Unemployed	301 (57.88%)	219 (42.12%)	520 (100%)	
	Retired/ Business	147 (49.83%)	148 (50.17%)	295 (100%)	
Educational status	Pre-high school/Uneducated	76 (35.51%)	138 (64.49%)	214 (100%)	0.001^*^
	High school or equal	609 (60.30%)	401 (39.70%)	1010 (100%)	
	Bachelor	1575 (66.48%)	794 (33.52%)	2369 (100%)	
	Post graduate certificate	165 (79.71%)	42 (20.29%)	207 (100%)	
Income level (SAR)	<2000	1108 (59.31%)	760 (40.69%)	1868 (100%)	0.001^*^
	2000- < 5000	554 (69.16%)	247 (30.84%)	801 (100%)	
	5000- < 7000	293 (77.31%)	86 (22.69%)	379 (100%)	
	7000- < 10000	202 (62.15%)	123 (37.85%)	325 (100%)	
	>10000	268 (62.76%)	159 (37.24%)	427 (100%)	
Money spent on NS monthly (SAR)	<500 -	1688 (59.94%)	1128 (40.06%)	2816 (100%)	0.001^*^
	500- < 1000 -	599 (76.60%)	183 (23.40%)	782 (100%)	
	1000- < 1500 -	113 (68.90%)	51 (31.10%)	164 (100%)	
	>1500 -	25 (65.79%)	13 (34.21%)	38 (100%)	
Smoking	Yes	211 (69.41%)	93 (30.59%)	304 (100%)	0.04^**^
	No	2214 (63.33%)	1282 (36.67%)	3496 (100%)	

Nutrition Supplement (NS), ^§^Chi-squared test, ^*^Highly significant (*p* < 0.01), and ^**^Significant (*p* < 0.05)

Active individuals who reported excellent health (1,352, 71.08%) were more likely to use dietary supplements, and this association was highly significant (p < 0.01). More than half (695, 68.88%) of the participants who had enrolled in a weight control program with a dietitian used dietary supplements, which was highly significant (p < 0.01). Participants who reported excellent fitness levels (800, 67.57%) were more likely to use dietary supplements, and this association was highly significant (p < 0.01). More than two-thirds (1,552, 68.19%) of those who engaged in ≥150 minutes of physical activity per week used dietary supplements, which was also highly significant (p < 0.01). Additionally, active individuals who were professional athletes (284, 73.77%) were more likely to use dietary supplements, and this association was highly significant (p < 0.01) (Table 2).

**Table 2:** Association between health characteristics and dietary supplement consumption among active individuals.

Variable	Do you consume nutritional supplements?			^§^P-value
	Yes (%)	No (%)	Total (%)	
	2425 (63.82%)	1375 (36.18%)	3800 (100%)	
How do you see your general health?				
Weak	4 (100.00%)	0 (0.00%)	4 (100%)	0.001^*^
Fair	895 (56.75%)	682 (43.25%)	1577 (100%)	
Good	174 (54.89%)	143 (45.11%)	317 (100%)	
Excellent	1352 (71.08%)	550 (28.92%)	1902 (100%)	
Have you ever enrolled in any weight control program with a dietitian?				
Yes	695 (68.88%)	314 (31.12%)	1009 (100%)	0.001^*^
No	1730 (61.98%)	1061 (38.02%)	2791 (100%)	
How do you see your general fitness level?				
Weak	31 (64.58%)	17 (35.42%)	48 (100%)	0.001^*^
Fair	1158 (64.58%)	635 (35.42%)	1793 (100%)	
Good	436 (56.26%)	339 (43.74%)	775 (100%)	
Excellent	800 (67.57%)	384 (32.43%)	1184 (100%)	
How long do you practice physical activity per week?				
< 150 min/wk	544 (59.85%)	365 (40.15%)	909 (100%)	0.001^*^
≥ 150 min/wk	1552 (68.19%)	724 (31.81%)	2276 (100%)	
Do not know	329 (53.50%)	286 (46.50%)	615 (100%)	
The level of professionalism in the sport?				
Yes	284 (73.77%)	101 (26.23%)	385 (100%)	0.001^*^
No	2141 (62.69%)	1274 (37.31%)	3415 (100%)	

§Chi-squared test, * Highly significant (*p* < 0.01), and ** Significant (*p* < 0.05).

Among active individuals, supplement use was predominantly concentrated in two categories. Protein supplements (37.00%) and vitamin/mineral supplements (36.11%) accounted for more than two-thirds of total usage combined, whereas all other supplement types were reported at substantially lower frequencies (S1 Fig). Most participants used dietary supplements daily (39.26%), followed by 4–6 times per week (18.93%) and 2–3 times per week (17.44%). A considerable proportion of participants reported having good knowledge of supplement ingredients, with 31.92% knowing most of the ingredients and 30.43% knowing all of them. More than half of the participants (53.69%) indicated that their supplements did not contain caffeine. Nearly half (46.14%) expressed confidence in the effectiveness and benefits of the supplements they used. The majority of participants spent (SAR) <500 per month on dietary supplements (69.61%), followed by 500–1,000 per month (24.70%) (Table 3).

**Table 3:** Knowledge and practices related to dietary supplement consumption among active individuals.

Variable	Categories	Active Individuals (%)
How often do you use nutritional supplements?	Daily	952 (39.26%)
	4-6 times/week	459 (18.93%)
	2-3 times/week	423 (17.44%)
	Once a week	383 (15.79%)
	Once a month	46 (1.90%)
	Once or less a month	162 (6.68%)
Which of the following represents your knowledge on nutritional supplement ingredients?	All the ingredients	738 (30.43%)
	Most of the ingredients	774 (31.92%)
	Some ingredients	690 (28.45%)
	None	223 (9.20%)
Do any of the supplements you use contain caffeine?	Yes	528 (21.77%)
	Do not know	595 (24.54%)
	No	1302 (53.69%)
How confident are you in the effectiveness and benefit of the nutritional supplement you used?	Very confident	664 (27.38%)
	Confident	1119 (46.14%)
	Somewhat confident	589 (24.29%)
	Not confident at all	53 (2.19%)
During the last three months, on average, how much money do you spend per month on nutritional supplements (SAR)	<500	1688 (69.61%)
	500- < 1000	599 (24.70%)
	1000- < 1500	113 (4.66%)
	≥1500	25 (1.03%)

**Fig 1:**
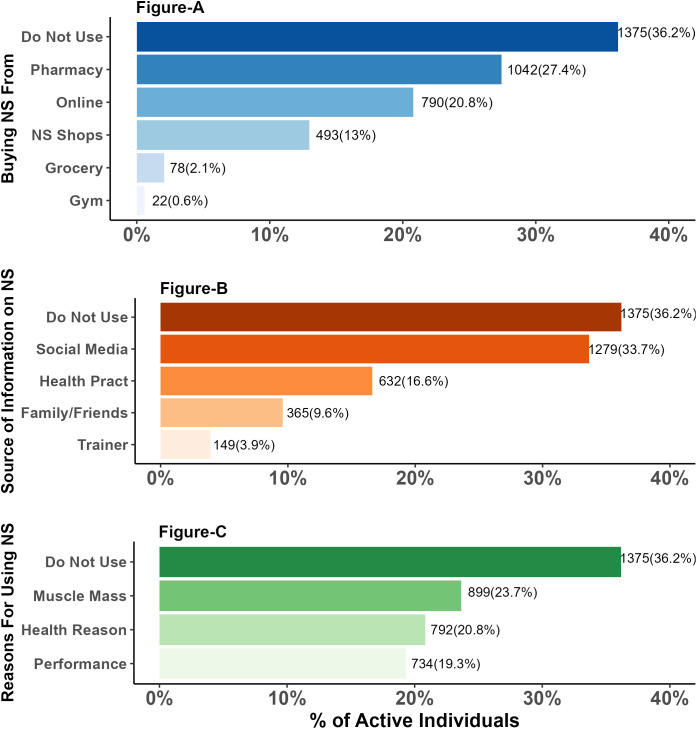
(A-C) Active individuals buying place, reasons for use and source of information on nutrition supplements.

More than one-fourth of participants (1,042, 27.4%) purchased dietary supplements from nearby pharmacies, followed by online sources (790, 20.8%) (Fig 1A). A large proportion of participants reported using dietary supplements to increase muscle mass (899, 23.7%), followed by general health improvement (792, 20.8%) (Fig 1B). The main source of information on dietary supplements was social media (1,279, 33.7%), followed by health practitioners (632, 16.6%) (Fig 1C).

**Fig 2:**
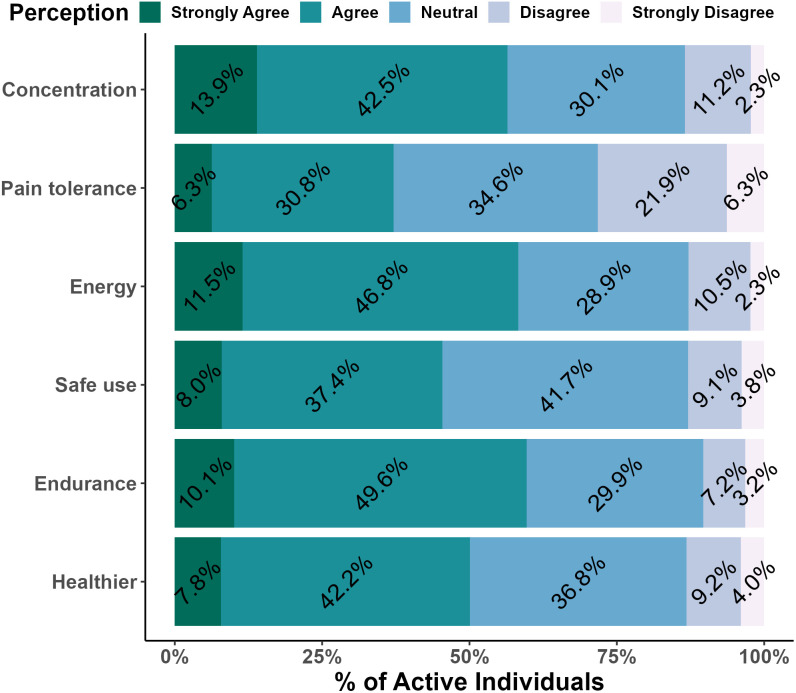
Participants’ perceptions on the use of nutrition supplements.

Fig 2 illustrates that participants’ perceptions had a positive influence on dietary supplement use. More than half of the participants strongly agreed or agreed, while less than one-fourth strongly disagreed or disagreed that dietary supplement use improved concentration, energy, endurance, and overall health. Fewer than half of the participants remained neutral regarding dietary supplement use for pain tolerance and safety.

**Fig 3:**
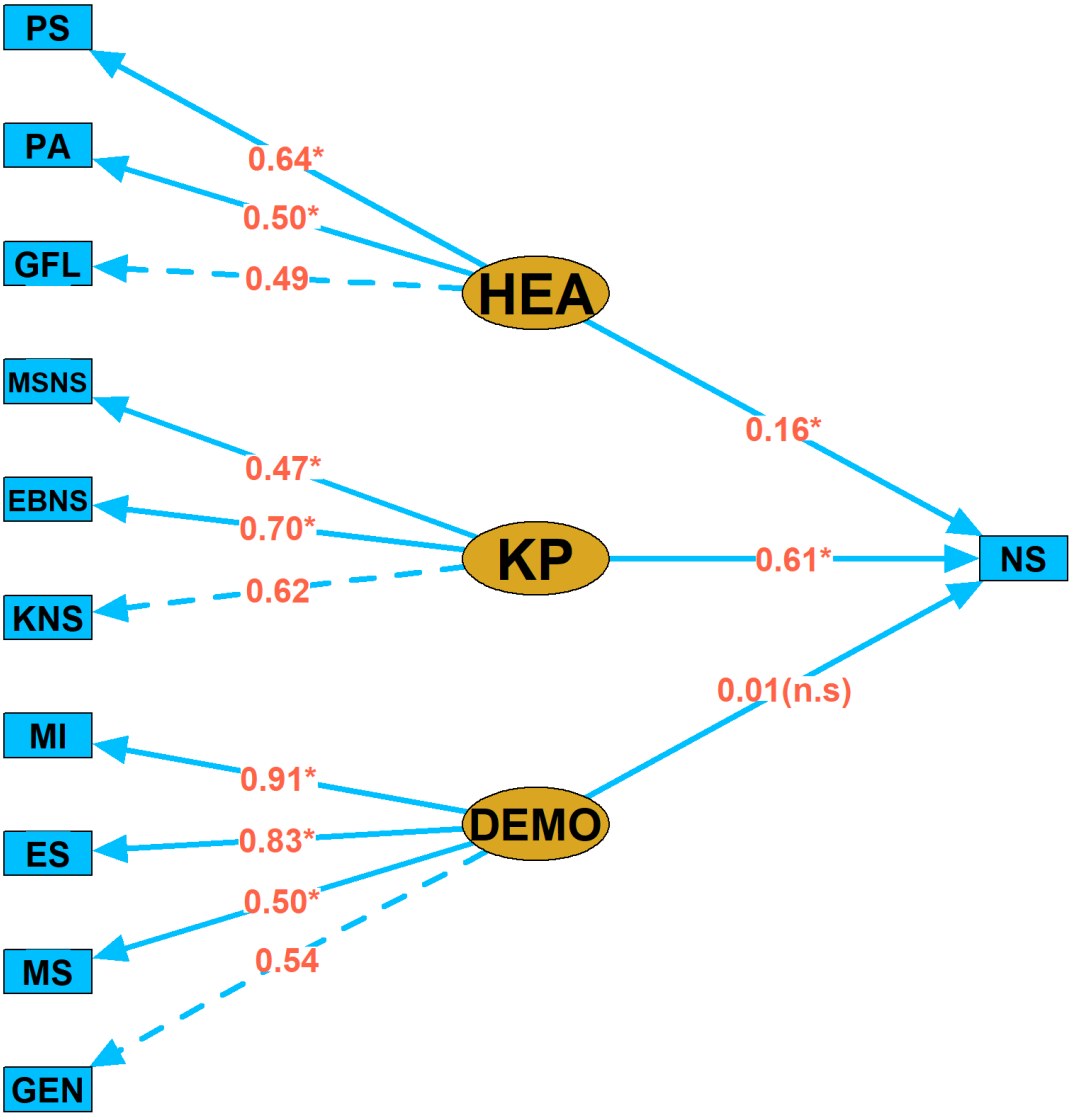
Factors associated with the consumption of nutrition supplements. Demographic and background characteristics (DEMO): GEN-Gender, MS-Marital status, ES-Employment status, MI-Monthly income per capita (SAR). Health characteristics (HEA): GFL-General fitness level, PA-Physical activity per week, PS-Professionalism in sport. Knowledge and practices (KP): UNS-Use nutritional supplements, KNS-Knowledge on nutritional supplement ingredients, EBNS- Effectiveness and benefit of the nutritional supplement, MSNS-Monthly spent on nutritional supplements (SAR) and *Highly Significant.

Bivariate analysis using the chi-square test (Table 1) revealed that age and nationality were non-significant predictors (p > 0.05) of dietary supplement consumption. Measurement validation was performed through confirmatory factor analysis, in which the items region, education level, and smoking under the demographic and background characteristics domain (DEMO). Additionally, the items from the health characteristics domain (HEA);use of supplements containing caffeine, enrollment in a weight control program with a dietitian, and general health did not meet the factor loading threshold of 0.40 (S2 Fig). Consequently, these eight variables were excluded from subsequent modeling to minimize model noise. Sequence Equation Modelling (SEM) results indicated χ2 = 771.703 (d.f. = 39, p = 0.00), comparative fit index = 0.953, root mean square error of approximation = 0.070 (90% CI: 0.066–0.075), and standardized root mean squared residual = 0.082. The three obtained domains; health (HEA = 0.16) and knowledge and practices (KP = 0.61) had a highly significant influence, whereas the demographic domain (DEMO = 0.01) did not show a significant association with dietary supplement use among active individuals. No evidence of collinearity was observed in the model (Fig 3).

## Discussion

The present study provides a national overview of dietary supplement consumption among active individuals in Saudi Arabia, with participation from 3,800 individuals. The findings demonstrated that dietary supplement use was highly prevalent, with significant differences observed across sociodemographic, health, and knowledge and practice variables. The overall prevalence of dietary supplement consumption among active individuals was 63.82%, which is consistent with recent Saudi studies conducted among female fitness-center users in Riyadh, reporting rates between 46% and 69% [14,15]. Compared with international findings, the prevalence observed in this study was higher than those reported in Cyprus (54%) [23], Romania (54%) [24], Portugal (44%) [25], and Brazil (37%) [26]. In terms of frequency, most active individuals consumed dietary supplements daily (39.2%), followed by 4–6 times per week (18.9%), suggesting that a substantial proportion incorporate supplements as a regular part of their diet. Although dietary supplements may offer health and performance benefits, their use can also carry potential risks of adverse effects [27]. In the United States, dietary supplements have been implicated in approximately 20% of drug-induced liver injury cases [28]. Additionally, more than 26,000 American military service members have reported adverse reactions to dietary supplements [29]. Prohormones, weight loss, and pre-/post-workout supplements were associated with the highest rates of adverse effects; herbal, multivitamin/mineral, and protein supplements showed moderate rates, whereas joint-health and single-vitamin or mineral products had the lowest rates [29]. These findings underscore the importance of promoting awareness among active individuals about the safe and informed use of dietary supplements.

Men, employed individuals, and those with medium to high monthly incomes reported higher dietary supplement consumption than other groups. This finding aligns with previous research, which shows that men and individuals with higher socioeconomic status tend to have better access to dietary supplements [30–32]. Moreover, participants with college or postgraduate qualifications demonstrated the highest rates of dietary supplement use. Consistent with this finding, the National Health and Nutrition Examination Survey also reported higher supplement consumption among individuals with college or higher education [33,34]. Recent data from Saudi Arabia further revealed that employed women were more likely to use dietary supplements than non-employed women [14], and supplement use was prevalent among educated, gym-attending women regardless of body mass index or income level [15]. Collectively, these findings highlight education as an important determinant of dietary supplement use.

Active individuals who rated their health and fitness levels as excellent were more likely to consume dietary supplements. Most participants (62%) reported knowing all or most of the ingredients in their dietary supplements, whereas a smaller proportion (28%) knew some, and 9% did not know any. Similarly, a study conducted in Portugal found that more than 70% of gym users stated they were well informed about dietary supplements, while only 4% reported feeling poorly informed [25]. Consistent with this, a recent Saudi study revealed that 82.5% of respondents were aware of dietary supplements; however, only 41% knew the correct dosages, and 30% expressed uncertainty regarding safety [16]. Nonetheless, these findings may reflect perceived rather than evidence-based knowledge, raising concerns about the reliability of self-reported awareness. Limited understanding of caffeine content may also predispose consumers to unintentional overconsumption and adverse effects, including sleep disturbances [35]. Most participants expressed confidence in the effectiveness and benefits of dietary supplements, consistent with findings from other studies [36]. Although regular dietary supplement use was common, reported monthly expenditure remained modest (approximately 500 SAR), suggesting that many purchases involved affordable or basic products.

Active individuals primarily purchased dietary supplements from pharmacies and online stores, followed by nutrition shops, grocery stores, and gyms. The most frequently reported reasons for dietary supplement use were to increase muscle mass, improve health, and enhance performance, respectively. Previous studies conducted among active Saudi individuals reported similar findings, with increasing muscle mass being the most common reason for supplement consumption [37–40]. Conversely, other studies identified bodybuilding [41,42], performance enhancement [12,43], improved appearance [9], energy provision [44], and fulfillment of nutritional requirements [14,45] as the main motivations. Collectively, these findings emphasize that the primary motivations for dietary supplement use among active individuals are to support health and improve physical fitness. Social media was identified as the predominant source of information on dietary supplements among active individuals, followed by healthcare professionals, family and friends, and gym coaches. In contrast, other Saudi studies reported that gym coaches were the primary source of information [37,39,42,43]. This shift may reflect a growing reliance on social media as an information channel. Therefore, evaluating the reliability of social media content and its influence on active individuals’ perceptions and attitudes toward dietary supplement use is warranted.

Most active individuals agreed that dietary supplements enhance endurance, boost energy levels, and improve concentration. These findings are consistent with previous studies conducted among active Saudi individuals [9,12,37,46,47]. Therefore, the results indicate a common belief in the potential of dietary supplements to improve both physical and cognitive performance. Furthermore, approximately half of the participants agreed that dietary supplements contribute to better overall health, similar to the findings of AlRuthia et al. [47]. Other studies have likewise reported that most active individuals believed dietary supplements made them healthier [9,12]. Participants’ perceptions were divided: some agreed that dietary supplements are safe to use and help increase pain tolerance, whereas others remained neutral. Similar proportions of respondents who agreed with the safety of dietary supplements were observed in previous studies [38,39]. Conversely, other studies involving gym attendees and professional football players reported that participants believed dietary supplements were safe to use [9,12], posed no adverse effects [42], and that protein supplements were not harmful [46]. These collective perceptions underscore the need for targeted education and awareness regarding the safety and efficacy of dietary supplements.

The structural equation model revealed complex relationships among demographic factors, health characteristics, knowledge and practices, and dietary supplement consumption. The analysis showed that greater knowledge (β = 0.61, p < 0.01) was significantly associated with higher dietary supplement consumption. For instance, awareness of the effectiveness and benefits of dietary supplements, as well as knowledge of their ingredients, was linked to increased consumption—likely due to greater confidence in their efficacy and safety. Additionally, higher monthly expenditure on dietary supplements was associated with a slight increase in consumption.

Overall, health characteristics demonstrated a weak but statistically significant positive association with dietary supplement consumption (β = 0.16, p < 0.01), suggesting that individuals engaged in regular fitness activities may perceive supplements as beneficial to their health. Notably, professionalism in sports exhibited a strong association with dietary supplement use, possibly reflecting the widespread belief that supplements enhance physical performance, endurance, and recovery. Furthermore, higher levels of weekly physical activity and better self-reported general fitness were positively associated with increased dietary supplement use. These findings suggest that individuals with greater involvement in physical activity and sports are more likely to incorporate dietary supplements into their health and performance optimization practices.

Demographic characteristics exhibited a very weak and non-significant association with dietary supplement consumption (β = 0.01, p > 0.05), indicating that demographic factors alone had limited explanatory power in predicting supplement use. Nevertheless, the analysis suggested that higher monthly income, employment status, marital status, and gender were related to dietary supplement consumption. Individuals with greater financial resources may have better access to dietary supplements, whereas employment and marital status may reflect lifestyle stability and health-related priorities that influence supplement use. Gender also demonstrated a positive, though relatively weak, association with dietary supplement consumption, suggesting that while gender differences exist, they play a minor role compared with other determinants. Overall, health and knowledge-related factors appear to have a more substantial influence on dietary supplement use than demographic characteristics among active individuals.

## Limitations

To the authors’ knowledge, this is the first study to evaluate dietary supplement consumption among active individuals on a national scale in Saudi Arabia. The study included a wide range of factors to comprehensively examine their associations with dietary supplement consumption. However, several limitations should be acknowledged. The cross-sectional design precludes the establishment of causal relationships, and the limited number of gym users may have introduced selection bias, leading to the under-representation of other groups such as community sports club members or informal exercisers. Furthermore, reliance on a self-reported questionnaire may have introduced recall bias, which could have influenced the accuracy of the responses. Future research should aim to evaluate the accuracy of knowledge regarding dietary supplements among active individuals in Saudi Arabia, including their understanding of general and sports nutrition, the efficacy and safety of dietary supplements, and recommended consumption guidelines. Identifying common misconceptions could help inform educational initiatives and awareness campaigns designed to improve knowledge dissemination and support informed decision-making about dietary supplement use.

## Conclusion

In conclusion, this study demonstrated that the prevalence of dietary supplement use among active individuals in Saudi Arabia was higher than prevelance rates reported in numerous international studies and consistent with findings from recent regional studies. In bivariate analysis, sociodemographic factors—including gender, employment status, income level, and education—were significantly associated with dietary supplement consumption; however, the demographic domain did not retain significance in the structural equation model. Health characteristics, such as self-rated health and fitness, were also positively associated with supplement use. Moreover, the study identified a shift toward social media as the primary source of information, reflecting changes in how active individuals obtain health-related knowledge. Future research should further investigate active individuals’ knowledge regarding the safety and effectiveness of dietary supplements to better assess their level of awareness. Accordingly, developing targeted educational and awareness programs is essential to promote safe, evidence-based, and informed dietary supplement use among active individuals in Saudi Arabia.

## Supporting information

S1 Table Sampling frame and the active individuals. Sample size (n) calculated by the Single proportion (Z2*P*(1-P))/e2. Where Z: Value from standard normal. Distribution corresponding to desired confidence level (Z = 1.96 for 95% CI), P: Expected true proportion: Desired precision, NR: Non Response Rate, Proportion: Percentage of individuals aged 18 years or above who practice sports activity (150 minutes and more per week) at the administrative region level (Source: Household Sports Practice Survey 2019, General Authority for Statistics).
(DOCX)

S2 Table Participants background characteristics. (DOCX)

S3 Table Participants health characteristics. (DOCX)

S1 Fig Consumption of dietary supplements by active individuals. (TIFF)

S2 Fig Confirmatory factor analysis. Demographic and background characteristics (DEMO): GEN-Gender, MS-Marital status, REG-Region, EL-Education level, ES-Employment status, MI-Monthly income per capita (SAR), SMO-Smoking. Health characteristics (HEA): GH-General health, EWC-Weight control program with a dietitian, GFL-General fitness level, PA-Physical activity per week, PS-Professionalism in sport. Knowledge and practices (KP): UNS-Use nutritional supplements, KNS-Knowledge on nutritional supplement ingredients, UCC-Use supplements contain caffeine, EBNS- Effectiveness and benefit of the nutritional supplement, MSNS-Monthly spent on nutritional supplements (SAR) and *Highly Significant” CFA plot indicated χ2 = 2619.559 (d.f = 116, p = 0.00), comparative fit index = 0.87, root mean square error of approximation = 0.075 (90% CI: 0.073–0.078), and standardized root mean squared residual = 0.100.
(TIFF)

S1 File Questionnaire english version. (PDF)
