# Supplementary material for: Dietary supplement consumption among active individuals in Saudi Arabia
Source: PLoS One. 2026 Jun 22;21(6):e0351208. doi: 10.1371/journal.pone.0351208 (PMC13286177; doi:10.1371/journal.pone.0351208)
Supplement: S1 Table — Sample size (n) calculated by the Single proportion (Z2*P*(1-P))/e2. Where Z: Value from standard normal. Distribution corresponding to desired confidence level (Z = 1.96 for 95% CI), P: Expected true proportion: Desired precision, NR: Non Response Rate, Proportion: Percentage of individuals aged 18 years or above who practice sports activity (150 minutes and more per week) at the administrative region level (Source: Household Sports Practice Survey 2019, General Authority for Statistics). (DOCX) [file pone.0351208.s001.docx]

### **Supplementary Information:**

| No. | Administrative Region | Proportion | Sample (n) | 20% NR | Desired Sample | Region |
| --- | --- | --- | --- | --- | --- | --- |
| 1 | Riyadh | 18.71 | 233.70 | 292.13 | 292 | Central |
| 2 | Makkah | 23.10 | 272.96 | 341.20 | 341 | Western |
| 3 | Madinah | 16.85 | 215.25 | 269.07 | 269 | Western |
| 4 | Qassim | 17.57 | 222.55 | 278.19 | 278 | Central |
| 5 | Eastern Region | 21.50 | 259.31 | 324.13 | 324 | Eastern |
| 6 | Asir | 17.32 | 220.07 | 275.09 | 275 | Southern |
| 7 | Tabuk | 17.44 | 221.21 | 276.51 | 277 | Northern |
| 8 | Hail | 16.22 | 208.84 | 261.06 | 261 | Central |
| 9 | Northern Borders | 17.54 | 222.27 | 277.84 | 278 | Northern |
| 10 | Jazan | 20.36 | 249.12 | 311.41 | 311 | Southern |
| 11 | Najran | 20.85 | 253.63 | 317.04 | 317 | Southern |
| 12 | Al-Baha | 17.24 | 219.28 | 274.11 | 274 | Southern |
| 13 | Al-Jouf | 18.60 | 232.67 | 290.83 | 291 | Northern |
| Total Percentage | | 20.04 |  |  | 3788 |  |

**Table S1. Sampling frame and the active individuals**

Sample size (n) calculated by the Single proportion (Z^2^*P*(1-P))/e^2^. Where Z: Value from standard normal. Distribution corresponding to desired confidence level (Z=1.96 for 95% CI), P: Expected true proportion: Desired precision, NR: Non Response Rate, Proportion: Percentage of individuals aged 18 years or above who practice sports activity (150 minutes and more per week) at the administrative region level (Source: Household Sports Practice Survey 2019, General Authority for Statistics)
