# Supplementary material for: Dietary supplement consumption among active individuals in Saudi Arabia
Source: PLoS One. 2026 Jun 22;21(6):e0351208. doi: 10.1371/journal.pone.0351208 (PMC13286177; doi:10.1371/journal.pone.0351208)
Supplement: S2 Table — (DOCX) [file pone.0351208.s002.docx]

### **Supplementary Information:**

**Table S2 Participants’ background characteristics**

| Variable | Categories | Frequency | Percentage |
| --- | --- | --- | --- |
| Gender | Male | 957 | 25.18% |
|  | Female | 2843 | 74.82% |
| Age | <25 | 2057 | 54.13% |
|  | ≥25 | 1743 | 45.86% |
| Nationality | Saudi | 3547 | 93.34% |
|  | Non-Saudi | 253 | 6.66% |
| Marital Status | Single | 2881 | 75.82% |
|  | Married | 841 | 22.13% |
|  | Divorced/Widowed | 78 | 2.05% |
| Region | Central | 843 | 22.18% |
|  | Eastern | 324 | 8.53% |
|  | Western | 610 | 16.05% |
|  | Northern | 846 | 22.26% |
|  | Southern | 1177 | 30.97% |
| Employment status | Student | 1860 | 48.95% |
|  | Employee | 1125 | 29.61% |
|  | Unemployed | 520 | 13.68% |
|  | Retired/ Business | 295 | 7.76% |
| Educational status | Pre-high school/Uneducated | 214 | 5.63% |
|  | High school or equal | 1010 | 26.58% |
|  | Bachelor | 2369 | 62.34% |
|  | Post graduate certificate | 207 | 5.45% |
| Income level  SAR | <2000 | 1868 | 49.16% |
|  | 2000-<5000 | 801 | 21.08% |
|  | 5000-<7000 | 379 | 9.97% |
|  | 7000-<10000 | 325 | 8.55% |
|  | >10000 | 427 | 11.24% |
| Smoking | Yes | 304 | 8% |
|  | No | 3496 | 92% |
