# Supplementary material for: Dietary supplement consumption among active individuals in Saudi Arabia
Source: PLoS One. 2026 Jun 22;21(6):e0351208. doi: 10.1371/journal.pone.0351208 (PMC13286177; doi:10.1371/journal.pone.0351208)
Supplement: S3 Table — (DOCX) [file pone.0351208.s003.docx]

### **Supplementary Information:**

**Table S3. Participants’ health characteristics**

| Variable | Categories | Frequency | Percentage |
| --- | --- | --- | --- |
| How do you see your general health? | Weak | 4 | 0.11% |
|  | Fair | 1577 | 41.5% |
|  | Good | 317 | 8.34% |
|  | Excellent | 1902 | 50.05% |
| Have you ever enrolled in any weight control program with a dietitian? | Yes | 1009 | 26.55% |
|  | No | 2791 | 73.45% |
| How do you see your general fitness level? | Weak | 48 | 1.26% |
|  | Fair | 1793 | 47.18% |
|  | Good | 775 | 20.39% |
|  | Excellent | 1184 | 31.16% |
| How long do you practice physical activity per week? | <150 min/WK | 909 | 23.92% |
|  | ≥150 min/WK | 2276 | 59.89% |
|  | Do not know | 615 | 16.18% |
| The level of professionalism in the sport? | Yes | 385 | 10.13% |
|  | No | 3415 | 89.87% |
