# Supplementary figures and images for: Dietary supplement consumption among active individuals in Saudi Arabia
Source: PLoS One. 2026 Jun 22;21(6):e0351208. doi: 10.1371/journal.pone.0351208 (PMC13286177; doi:10.1371/journal.pone.0351208)

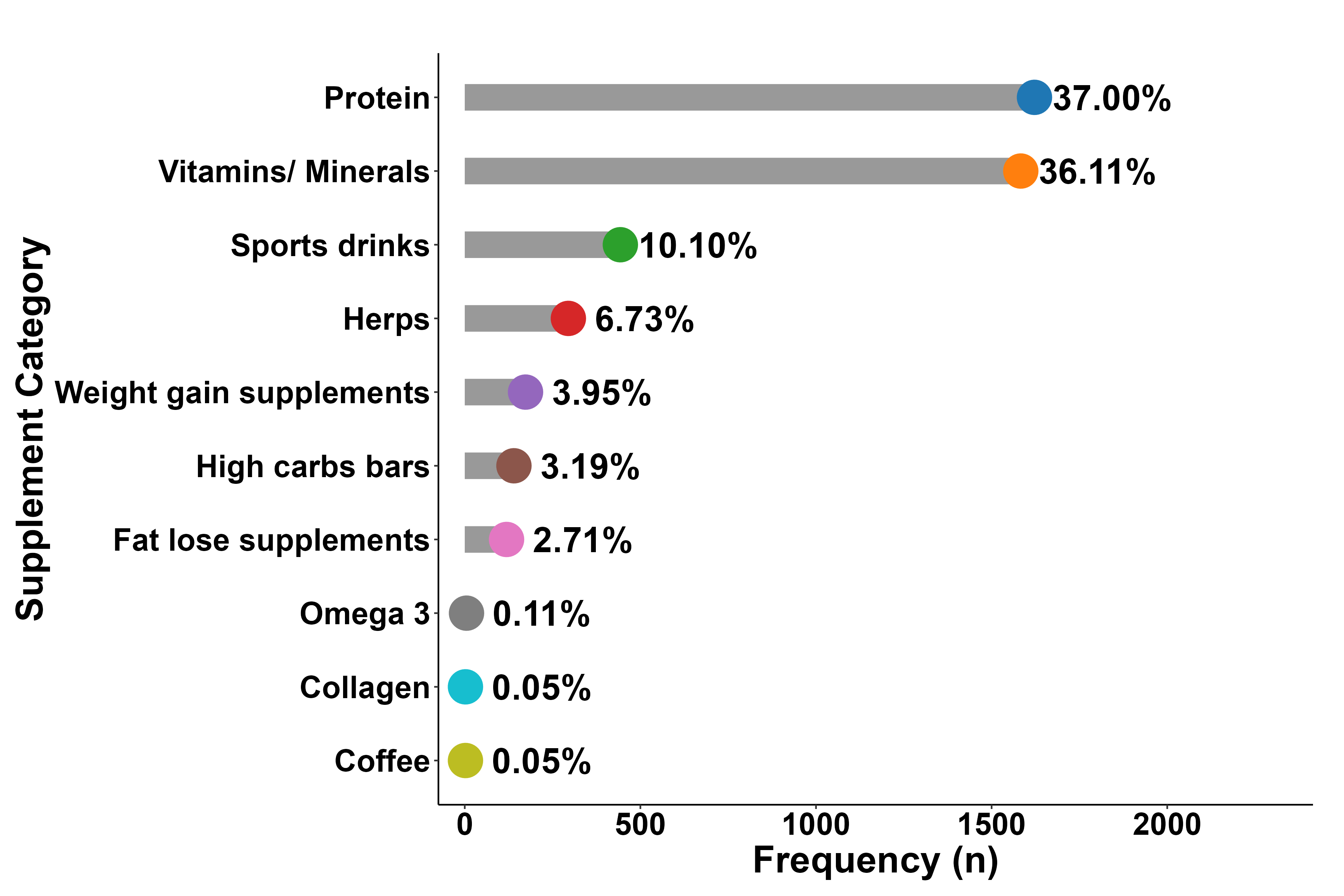

Supplement: S1 Fig — (TIFF) [file pone.0351208.s004.tiff]

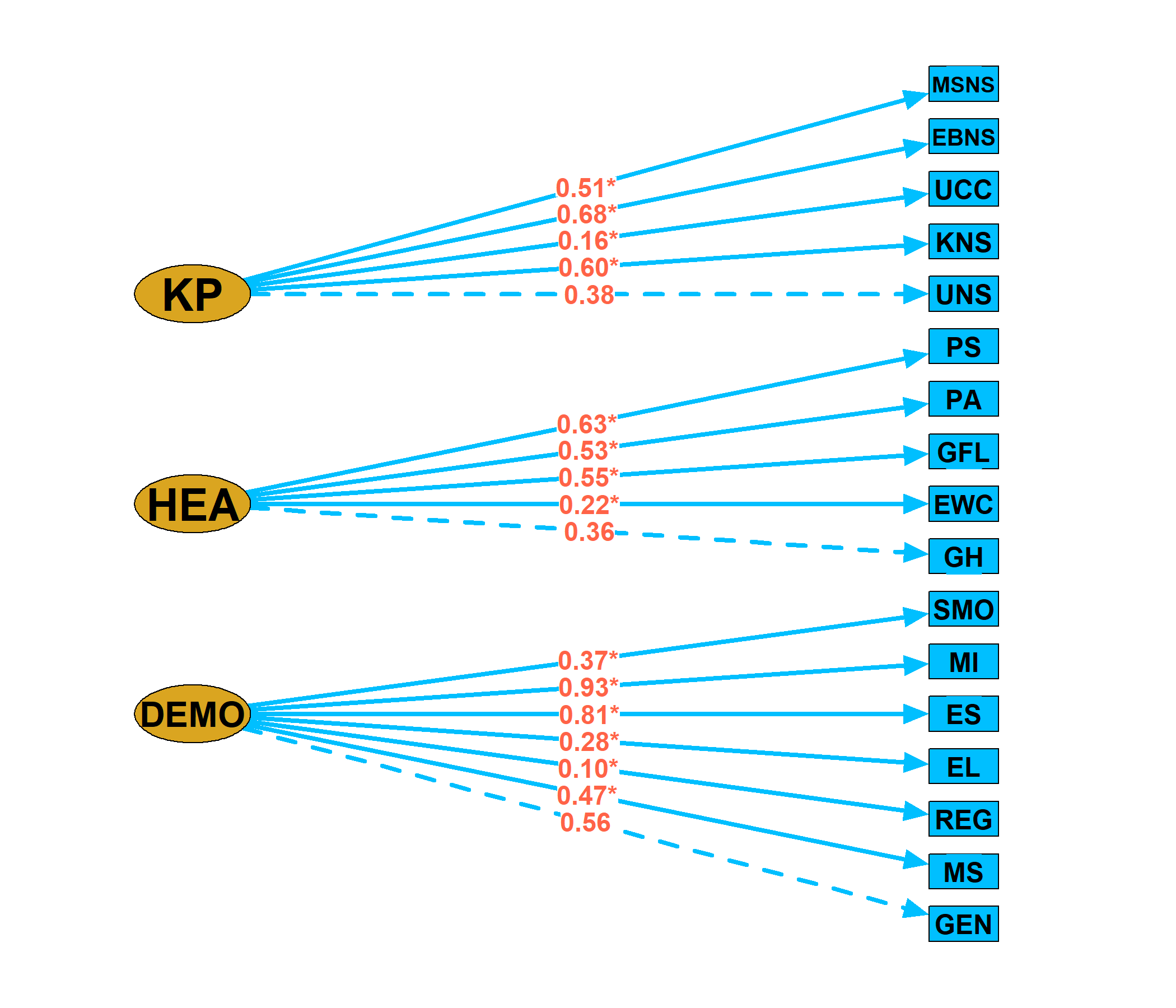

Supplement: S2 Fig — Demographic and background characteristics (DEMO): GEN-Gender, MS-Marital status, REG-Region, EL-Education level, ES-Employment status, MI-Monthly income per capita (SAR), SMO-Smoking. Health characteristics (HEA): GH-General health, EWC-Weight control program with a dietitian, GFL-General fitness level, PA-Physical activity per week, PS-Professionalism in sport. Knowledge and practices (KP): UNS-Use nutritional supplements, KNS-Knowledge on nutritional supplement ingredients, UCC-Use supplements contain caffeine, EBNS- Effectiveness and benefit of the nutritional supplement, MSNS-Monthly spent on nutritional supplements (SAR) and *Highly Significant” CFA plot indicated χ2 = 2619.559 (d.f = 116, p = 0.00), comparative fit index = 0.87, root mean square error of approximation = 0.075 (90% CI: 0.073–0.078), and standardized root mean squared residual = 0.100. (TIFF) [file pone.0351208.s005.tiff]
