# Supplementary material for: Dietary supplement consumption among active individuals in Saudi Arabia
Source: PLoS One. 2026 Jun 22;21(6):e0351208. doi: 10.1371/journal.pone.0351208 (PMC13286177; doi:10.1371/journal.pone.0351208)
Supplement: S1 File — (PDF) [file pone.0351208.s006.pdf]

Questionnaire

The consumption of dietary supplements among active individuals in Saudi Arabia

Dear participant,

We appreciate your valuable time and collaboration. Please check (√) one answer for each question

Demographic characteristics:

1. Gender? ☐ Male ☐ Female

2. What is your age today? ☐ <25 years ☐ 25-45 years ☐ 46- 60 years ☐ >60years

3. What is your height in cm? (Without shoes)? ☐ <160cm ☐ 160-180cm ☐ >180cm

4. Your weight in kilograms (light cloths)? ☐ <60kg ☐ 60-80kg ☐ >80kg

5. What is your ethnic background? ☐ Arabian ☐ Asian ☐ African ☐ Caucasian

6. What is your region?

☐ Northern region ☐ Southern region ☐ Eastern region ☐ Western region

☐ Central region

7. Please indicate the highest level of educational completion.

☐ High school/equal ☐ Pre high/uneducated

☐ Bachelor's degree ☐ Post graduate certificate

8. What is your marital status? ☐ Single ☐ Married ☐ Widowed/Divorced

9. What is your nationality? ☐ Saudi ☐ Non Saudi

10. Monthly how much money do you spent on nutritional supplements?

☐ 500-1000 ☐ 1000-1500 ☐ 1500 more ☐ Nothing

11. What is your employment status?

☐ Student ☐ Employed ☐ Unemployed ☐ Retired/Business

12. What is the estimated monthly income for your family?

☐ Less than 2,000 ☐ 2,000 to 5,000 ☐ 5,000 to 7,000

☐ 7,000 to 10,000 ☐ 10,000 more

Health Characteristics:

13. How do you see your general health? ☐ Excellent ☐ Good ☐ Fair ☐ Weak

14. How do you see your general eating habits?

☐ Excellent ☐ Good ☐ Fair ☐ Weak

15. Which of the following represents your implementation on weight?

☐ Lose weight ☐ Gain weight ☐ Maintaining weight

16. Have you ever enrolled in any weight control program with a nutritionist?

☐ Yes ☐ No

17. Which of the following diets represent your current diet?

☐ Low calories ☐ Vegetarian / Vegan ☐ Low fat

☐ High protein ☐ High carbohydrates ☐ Keto Diet

☐ No special diet Otherwise, please specify: ____________________

18. How long do you practice physical activity /week?

☐ <150 min/week ☐ ≥150 min/week ☐ Do not know

19. Do you smoke?

☐ Yes ☐ No

Awareness on dietary supplements:

20. Dietary supplements make me healthier.

☐ Strongly Agree ☐ Agree ☐ Neutral ☐ Disagree ☐ Strongly Disagree

21. Dietary supplements improve my endurance.

☐ Strongly Agree ☐ Agree ☐ Neutral ☐ Disagree ☐ Strongly Disagree

22. Dietary supplements are safe to use

☐ Strongly Agree ☐ Agree ☐ Neutral ☐ Disagree ☐ Strongly Disagree

23. Dietary supplements provide me with more energy

☐ Strongly Agree ☐ Agree ☐ Neutral ☐ Disagree ☐ Strongly Disagree

24. Dietary supplements increase my strength

☐ Strongly Agree ☐ Agree ☐ Neutral ☐ Disagree ☐ Strongly Disagree

25. Dietary supplements increase my ability to tolerate the pain

☐ Strongly Agree ☐ Agree ☐ Neutral ☐ Disagree ☐ Strongly Disagree

26. Dietary supplements improve my concentration

☐ Strongly Agree ☐ Agree ☐ Neutral ☐ Disagree ☐ Strongly Disagree

Fitness Questions:

27. How do you see your general fitness level?

☐ Excellent ☐ Good ☐ Fair ☐ Weak

28. Do you a professional Athlete?

☐ Yes ☐ No

29. The level of professionalism in the gym?

☐ Unprofessional ☐ Beginner ☐ Practitioner ☐ Professional

30. How long have you been using gym?

☐ < 1 year ☐ 1-2 year ☐ 2-3 years ☐ >3 years

31. Why do you go to gym? (Please tick all that apply)

☐ Health reasons ☐ Increase muscle mass ☐ Preparing for a competition

☐ Fun ☐ Weight loss ☐ Others ________________

32. What kind of exercise do you do in the gym?

☐ Cardiovascular ☐ Weight training ☐ Mixed ☐ Others

Dietary supplements Usage:

33. Do you use nutritional supplements?

☐ Yes ☐ No

34. What kind of nutrition supplement do you use? (Choose all relative options)

☐ Protein powder ☐ Vitamins/minerals ☐ Sport drinks

☐ High carb bars ☐ Fat loos supplements ☐ Weight gain supplements

☐ Herbs ☐ Amino Acids ☐ Others (Please Specify)…….

35. How often do you use nutritional supplements?

☐ Daily ☐ 2-3 times/week ☐ 4-6 times/week ☐ Once/week nutritional

☐ Once/month ☐ Never

36. What are your reasons for using nutritional supplements?

☐ Increase muscle mass ☐ Improve the performance ☐ Health reasons

37. Which of the following represents your knowledge on nutritional supplement ingredients

☐ All components ☐ Most of the ingredients ☐ Some ingredients

☐ None of the ingredients

38. Do any of the supplements you use contain caffeine?

☐ Yes ☐ No ☐ I don't know

39. Do you think that nutritional supplements comply with Saudi requirements?

☐ Yes ☐ No ☐ I don't know

40. Do you think the nutritional supplements have some health benefits?

☐ Yes ☐ No ☐ I don't know

41. How confident are you that your supplements will do as they claim?

☐ Very confident ☐ Confident ☐ Somewhat confident

☐ Not confident at all

42. How confident are you that your supplements are safe to consume?

☐ Very confident ☐ Confident ☐ Somewhat confident

☐ Not confident at all

43. What or who is the source of your nutritional supplement information?

☐ Family members ☐ Friends ☐ Personal trainer

☐ Health professional (such as doctor, nurse, dietitian, etc.)

☐ Journals (such as Men's Health, Muscles, Fitness, Flexibility, Figure, and Self)

☐ Online ☐ Store sales representative ☐ Television

☐ Social Media ☐ Others (Please specify)________________________

44. Where do you usually buy your nutritional supplements? (Please tick all that apply)

☐ Food supplement stores in Saudi Arabia ☐ Shops outside Saudi Arabia

☐ Drug store (pharmacies) ☐ Gym / fitness centre ☐ I don't know

☐ Grocery store ☐ Another source (Please specify): ________________

45. During the last three months, on average, how much money do you spent per month on nutritional supplements?

☐ 1-499 riyals ☐ 500-999 riyals ☐ 1000-1499 riyals ☐ ≥1500 riyals

☐ Other (Please specify) ....

46. Are you avoiding any supplements?

☐ Yes ☐ No

47. If yes, why you avoiding them? (Please tick all that apply)

☐ Health reasons ☐ Not recommended by a nutritionist

☐ Expensive ☐ Don't like the taste ☐ Others Please specify ………..

*****Thank you for your participation*****
