# Supplementary material for: Dietary supplement consumption among active individuals in Saudi Arabia
Source: PLoS One. 2026 Jun 22;21(6):e0351208. doi: 10.1371/journal.pone.0351208 (PMC13286177; doi:10.1371/journal.pone.0351208)
Supplement: Attachment — Submitted filename: Response to Reviewers.docx [file pone.0351208.s009.docx]

**Response to Additional Editor Comments - PLOS ONE**

**The consumption of dietary supplements among active individuals in Saudi Arabia**
**Manuscript ID: PONE-D-25-22005**

Dear Academic Editor,

Jordan Llego, PhD ELM, D. Hon. Ex., PhDN, RN.

Thank you for the opportunity to revise our manuscript and for the insightful comments provided by you. We appreciate the time and effort invested in evaluating our work. We have carefully considered all feedback and revised the manuscript accordingly. Below, we provided a detailed point-by-point response to each comment.

**Comment 1:** One major concern is the structural equation modeling. Although you recalculated the model using the recommended WLSMV estimator, the fit indices are still below the journal’s standards, and the reporting needs to be more transparent. Key details—such as confirmatory factor analysis results, factor loadings, measurement model validation, and reliability for latent constructs—are missing. The reasoning behind grouping different items into latent variables also needs more explanation. Additionally, age and nationality are still presented as if they’re psychometric constructs rather than categorical predictors. These points affect the statistical validity of the model and should be clarified or reconsidered.

**Author Response:** Thank you for this valuable observation. We followed point by point carefully to respond. Bivariate analysis using chi-square (Table 1) identified that age and nationality variables are non-significant predictors (p > 0.05) for the consumption of nutritional supplements. Measurement validation through Confirmatory Factor Analysis (CFA), where items Region (REG) and use supplements contain caffeine (UCC) were not met the factor loadings threshold 0.20 (Figure S2). Justifying the exclusion of four variables from subsequent modelling to reduce noise, the structural equation modelling (SEM) plot indicating 𝑥2 = 2047.530 (d.f = 99, p = 0.00), comparative fit index = 0.901, root mean square error of approximation = 0.072 (90% CI: 0.069–0.075), and standardized root mean squared residual = 0.098. The obtained three factors’ domains health (HEA= 0.11), and knowledge and practices (KP = 0.71) had a highly significant influence, while of demographic domain (DEMO = −0.02) not shown a significant relation on the usage of nutrition supplements among the active individuals, and there was no evidence of collinearity in the model (Figure 3). **[Manuscript** **Line 272-283]**

**Figure 3 annotate have been changed to** “Demographic and background characteristics (DEMO): GEN-Gender, MS-Marital status, EL-Education level, ES-Employment status, MI-Monthly income per capita (in Saudi riyals "SR "), SMO-Smoking. Health characteristics (HEA): GH-General health, EWC-Weight control program with a dietitian, GFL-General fitness level, PA-Physical activity per week, PS-Professionalism in sport. Knowledge and practices (KP): UNS-Use nutritional supplements, KNS-Knowledge on nutritional supplement ingredients, EBNS- Effectiveness and benefit of the nutritional supplement, MSNS-Monthly spent on nutritional supplements - (SR ) and *Highly Significant” **[Manuscript** **Line 572-579]**

**Added supplementary figure and annotate [Manuscript** **Line 595]**

**Figure S2. Confirmatory factor analysis**

Demographic and background characteristics (DEMO): GEN-Gender, MS-Marital status, REG-Region, EL-Education level, ES-Employment status, MI-Monthly income per capita (in Saudi riyals "SR "), SMO-Smoking. Health characteristics (HEA): GH-General health, EWC-Weight control program with a dietitian, GFL-General fitness level, PA-Physical activity per week, PS-Professionalism in sport. Knowledge and practices (KP): UNS-Use nutritional supplements, KNS-Knowledge on nutritional supplement ingredients, UCC-Use supplements contain caffeine, EBNS- Effectiveness and benefit of the nutritional supplement, MSNS-Monthly spent on nutritional supplements - (SR ) and *Highly Significant” CFA plot indicated 𝑥2 = 2619.559 (d.f = 116, p = 0.00), comparative fit index = 0.87, root mean square error of approximation = 0.075 (90% CI: 0.073–0.078), and standardized root mean squared residual = 0.100. **[Manuscript** **Line 596-606]**

**Comment 2:** The sampling strategy also needs clearer explanation. While you’ve clarified some parts of the process, the manuscript still doesn’t mention how many gyms were contacted, the response rate, or exactly how volunteers and administrators were involved in recruiting participants. It’s also not clear how representative the sample is, since the study uses non-probability purposive sampling among gym-goers, but the conclusions suggest national-level generalizability. Please consider either adjusting the claims or providing more detail to make the methodology transparent.

**Author Response: Thank to the Editor for this valuable observation, we have responded point by point.** A total of 90 gym centers were visited across the 13 provinces of Saudi Arabia, assuming an average number of gym users is 50 per each center. Based on this assumption, 4,500 eligible participants were approached. Google Maps or local business listings were used to compile lists of gymnasiums (the study was limited to gym users). Selected gym, eligible participants list with WhatsApp number collected from the gym volunteer/administrators and enrolled the participants using systematic random sampling method to ensure the representativeness of gym users from the entire nation. Every K^th^ participant (K = Total eligible participants in the selected gym/50) was included systematically from the list until to get the 50 participants from selected gym centers. If any participant not willing then included the following participant for the study. The online questionnaire was distributed through social media WhatsApp with the help of volunteer or gym administrators. In total 3,800 gym users participated with 84% of response rate. **[Manuscript** **Line 159-171]**

**Comment 3:** The data availability statement still doesn’t meet PLOS ONE requirements. The journal asks that the minimal dataset be publicly available unless there’s a strong ethical or legal reason not to. Saying that data is available upon request isn’t enough. Please update this section either by depositing the dataset in a public repository or by giving a clear justification, supported by your institution’s policy, and naming a data access committee if needed.

**Author Response: Thanks to the editor for this valuable comment, we have updated the data availability statement.** Data cannot be shared publicly because the data contains potentially identifying information. The research committee at the Clinical Nutrition Department, Jazan University has imposed data sharing restrictions on this study. Data requests can be directed to the corresponding author and to the chair of the research committee at the department (Dr. Huda Mubaraki [cln.ams@jazanu.edu.sa](mailto:cln.ams@jazanu.edu.sa)) for researchers who meet the criteria for access to confidential data.

**Comment 4:** The way dietary supplements are defined and categorized still needs work. You’ve included the DSHEA definition, but the results don’t separate out the different types of supplements participants used. Because “dietary supplements” covers such a wide range, not breaking them down makes it hard to interpret the findings. Reviewers specifically asked for a clear distinction between products like vitamins, herbs, protein powders, energy drinks, and prescribed supplements. This breakdown should be included in both the methods and results sections.

**Author Response: Thanks to the editor for this valuable comment.**

**In methods After dietary supplements definition:** Based on the frequency of consumption of dietary supplements categized into ten main items: Protein (such as amino acids, protein bar, protein powder, creatine, protein milk), vitamins/ minerals (such as multivitamins, vitamin A, vitamin B complex, and vitamin C, calcium, selenium, zinc, and iron), sports drinks, herps, weight gain supplements, High carps bar, Fat lose supplements, omega 3, Collagen and coffee **[Manuscript** **Line 146-151].**

**In results beginning of the table 3 narration:** Among active individuals, supplement use was predominantly concentrated in two categories. Protein (37.00%) and vitamins/minerals (36.11%) were used by more than two-thirds of participants combined, whereas all other supplement types were reported at substantially lower frequencies (Figure S1) **[Manuscript** **Line 243-246]**

**Added supplementary figure [Manuscript** **Line 594]**

**Figure S1. Consumption of dietary supplements by active individuals**

**Comment 5:** The questionnaire documentation also needs more detail. While you mention a pilot study and approval from the National Nutrition Committee, the manuscript still doesn’t explain how the questionnaire was adapted and translated, how cognitive testing was done, or provide psychometric details like internal consistency or construct validity. These are important for transparency and reproducibility, especially since the instrument is self-developed and key to your study. Please also include the full questionnaire or its item list as supplementary material.

**Author Response: Thanks to the editor for this valuable observation, we have responded point by point and also uploaded questionnaire as supplementary file.**

After a critical review of local and international studies validated tools assessing nutritional supplements use, a questionnaire was adapted with minor modifications to ensure cultural relevance (12,18). Translation into Arabic followed a standardized forward–backward translation process using independent bilingual translators. Face validity was assessed through pilot testing with 25 gym users. Participants were asked to comment on clarity, wording, and understanding of each item. Minor linguistic refinements were made based on participant feedback and the attained Cronbach’s alpha (0.81) entitling the acceptable internal consistency. Content validity was evaluated by the experts from national nutrition committee, Saudi Arabia. Reliability was assed using test-retest method. In which a sample of 25 gym users completed questionnaire twice within two weeks. The outcomes of the two times compared using correlation co-efficient that has shown a significantly stability co-efficient suggesting a good test-retest reliability. A threshold of factor loading greater than 0.2, comparative fit index 0.901 and root mean square error of approximation 0.072 through the confirmatory factor analysis supporting the construct validity of the questionnaire. **[Manuscript** **Line 86-100]**

**Comment 6:** There are also some conceptual and interpretive inconsistencies to address. The abstract, discussion, and conclusion still imply that dietary supplements affect health and performance, even though the study didn’t measure these outcomes. If perceived effects are part of your objectives, please make this clear in your analysis and presentation. If not, revise the conclusions so they match the descriptive and correlational nature of your data, and avoid using causal language throughout the manuscript.

**Author Response: Thanks to the editor for this valuable observation and we have rewritten the conclusion.** In conclusion, this study revealed that the prevalence of dietary supplement use among active individuals in Saudi Arabia was higher than rates reported internationally and consistent with recent regional studies. Sociodemographic factors, such as gender, employment status, level of income, and education level, showed a significant difference in dietary supplement consumption. Furthermore, health characteristics, such as self-rated health and fitness, were positively associated with dietary supplement consumption. Additionally, the study noted that there was a shift towards social media being the main source of information, which reflects changes in where active individuals obtain health-related information. Further studies are needed to examine the knowledge of active individuals regarding the safety and effectiveness to determine the level of awareness. Hence, it is very important to tailor awareness programs and educate active individuals effectively. **[Manuscript** **Line 408-418]**
