# Supplementary material for: Dietary supplement consumption among active individuals in Saudi Arabia
Source: PLoS One. 2026 Jun 22;21(6):e0351208. doi: 10.1371/journal.pone.0351208 (PMC13286177; doi:10.1371/journal.pone.0351208)
Supplement: Attachment — Submitted filename: Response to Reviewers 2.docx [file pone.0351208.s008.docx]

**Response to Reviewers - PLOS ONE**

**The consumption of dietary supplements among active individuals in Saudi Arabia**
**Manuscript ID: PONE-D-25-22005**

Dear Academic Editor,

Jordan Llego, PhD ELM, D. Hon. Ex., PhDN, RN.

Thank you for the opportunity to revise our manuscript and for the insightful comments provided by you and the reviewers. We appreciate the time and effort invested in evaluating our work. We have carefully considered all feedback and revised the manuscript accordingly. Below, we provided a detailed point-by-point response to each comment.

**#Journal Requirements and Author Response**

**Comment 1:** Please ensure that your manuscript meets PLOS ONE's style requirements, including those for file naming. The PLOS ONE style templates can be found at

<https://journals.plos.org/plosone/s/file?id=wjVg/PLOSOne_formatting_sample_main_body.pdf> and

[https://journals.plos.org/plosone/s/file?id=ba62/PLOSOne_formatting_sample_title_authors_affiliations.pdf](https://track.editorialmanager.com/CL0/https:%2F%2Fjournals.plos.org%2Fplosone%2Fs%2Ffile%3Fid=ba62%2FPLOSOne_formatting_sample_title_authors_affiliations.pdf/1/010f0199318487fa-f3462267-9edc-48f4-b904-a7dc5af6e3cf-000000/OmoBchGPr-EVCFs05-1t04httd5N1kwnfEOAut6oK6k=228)

**Author Response:** Thank you for the links provided. We followed the instructions carefully for the development of revised manuscript and also labelled the files for submission as:

‘Response to Reviewers’

‘Revised Manuscript with Track Changes’

‘Manuscript’

**Comment 2:** Please provide additional details regarding participant consent. In the ethics statement in the Methods and online submission information, please ensure that you have specified (1) whether consent was informed and (2) what type you obtained (for instance, written or verbal, and if verbal, how it was documented and witnessed). If your study included minors, state whether you obtained consent from parents or guardians. If the need for consent was waived by the ethics committee, please include this information.

If you are reporting a retrospective study of medical records or archived samples, please ensure that you have discussed whether all data were fully anonymized before you accessed them and/or whether the IRB or ethics committee waived the requirement for informed consent. If patients provided informed written consent to have data from their medical records used in research, please include this information.

**Author Response:** Data collected through online questionnaire. We agree with the editor observation, and have added details regarding participant consent.

### **Ethical consideration**

Ethical issues were addressed in accordance with the guidelines of the Declaration of Helsinki and the Saudi Bioethics standards. Participants were informed through a structure narrative at the beginning of the Google Form questionnaire about the study purpose, their right to withdraw at any time with no penalty, and assurance regarding the confidentiality of their response. Digitally recorded the written informed consent before participate in the survey. Ethical clearance for this study was granted by the Standing Committee for Scientific Research of Princess Nourah Bint Abdulrahman University (PNURSP2025R207), Riyadh, Saudi Arabia.

**[Page 6, line 168-183].**

**Comment 3:** Thank you for stating the following financial disclosure:
“Princess Nourah Bint Abdulrahman University Researchers Supporting Project number (PNURSP2025R207).”
Please state what role the funders took in the study. If the funders had no role, please state: 'The funders had no role in study design, data collection and analysis, decision to publish, or preparation of the manuscript.'
If this statement is not correct you must amend it as needed.
Please include this amended Role of Funder statement in your cover letter; we will change the online submission form on your behalf.

**Author Response:** Thanks to the editor for this suggestion. We have added the statement that “Princess Nourah Bint Abdulrahman University Researchers Supporting Project number (PNURSP2025R207), Riyadh, Saudi Arabia. The funders had no role in study design, data collection and analysis, decision to publish, or preparation of the manuscript.” and also mentioned the same in the cover letter **[Page 18, line 464-467].**
**Comment 4:** Thank you for stating the following in the Acknowledgments Section of your manuscript: “the authors would like to acknowledge and appreciate all participants for their cooperation in filling out the survey to complete this study, in addition to deep grateful for Princess Nourah bint Abdulrahman University for funding and supporting this work.”
We note that you have provided funding information that is currently declared in your Funding Statement. However, funding information should not appear in the Acknowledgments section or other areas of your manuscript. We will only publish funding information present in the Funding Statement section of the online submission form.
Please remove any funding-related text from the manuscript and let us know how you would like to update your Funding Statement. Currently, your Funding Statement reads as follows:
“Princess Nourah Bint Abdulrahman University Researchers Supporting Project number (PNURSP2025R207).”
Please include your amended statements within your cover letter; we will change the online submission form on your behalf.

**Author Response:** Thanks to the editor for this observation. We have removed funding-related text from acknowledgement section and updated the narrative as “The authors would like to acknowledge and appreciate all participants for their cooperation in filling out the survey to complete this study” **[Page 18, line 470-471]**

Updated funding statement: “Princess Nourah Bint Abdulrahman University Researchers Supporting Project (PNURSP2025R207), Riyad, Saudi Arabia**.**” **[Page 18, line 464-467].**

We have included the amended funding statements in the cover letter

**Comment 5:** We note that your Data Availability Statement is currently as follows: All relevant data are within the manuscript and its Supporting Information files.

Please confirm at this time whether or not your submission contains all raw data required to replicate the results of your study. Authors must share the “minimal data set” for their submission. PLOS defines the minimal data set to consist of the data required to replicate all study findings reported in the article, as well as related metadata and methods

(<https://journals.plos.org/plosone/s/data-availability#loc-minimal-data-set-definition>).

For example, authors should submit the following data:
- The values behind the means, standard deviations and other measures reported;
- The values used to build graphs;
- The points extracted from images for analysis.
Authors do not need to submit their entire data set if only a portion of the data was used in the reported study.
If your submission does not contain these data, please either upload them as Supporting Information files or deposit them to a stable, public repository and provide us with the relevant URLs, DOIs, or accession numbers. For a list of recommended repositories, please see <https://journals.plos.org/plosone/s/recommended-repositories>.
If there are ethical or legal restrictions on sharing a de-identified data set, please explain them in detail (e.g., data contain potentially sensitive information, data are owned by a third-party organization, etc.) and who has imposed them (e.g., an ethics committee). Please also provide contact information for a data access committee, ethics committee, or other institutional body to which data requests may be sent. If data are owned by a third party, please indicate how others may request data access.

**Author Response:** Thanks to the editor for this valuable observation. We updated the data availability statement as “The datasets generated and analyzed during the current study are available from the corresponding author upon reasonable request. Due to ethical considerations and privacy regulations, the data cannot be made publicly accessible. In fact, institutional guidelines mandate controlled access to safeguard potentially identifiable health information”.

We provided the raw data Excel file as supporting information [V1 (29092025 - V8_NS).xlsx] to replicate and assess the accuracy of the results by publishing authority.

**Comment 6:** Please include a separate caption for each figure in your manuscript.

**Author Response:** Thank you for this observation. We have removed Figure 1 from the manuscripts and adjusted the other figure numbers. Written separate caption for each figure in the manuscript.

Remove “Fig 1” and include “Table S1” **[Page 5, Line 147].**

“Fig 2A” changed to “Fig 1A” **[Page 11, Line 250].**

“Fig 2B” changed to “Fig 1B” **[Page 12, Line 252].**

“Fig 2C” changed to “Fig 1C” **[Page 12, Line 253].**

**Caption:** “**Figure 1(A-C) Active individuals buying place, reasons for use and source of information on nutrition supplements**” **[Page 23, line 608-609].**

“Fig 3” changed to “Fig 2” **[Page 12, Line 256].**

**Caption:** “**Figure 2 Participants’ perceptions on the use of nutrition supplements**” **[Page 23, line 611].**

“Fig 4” changed to “Fig 3” **[Page 12, Line 271**].

**Caption:** “**Figure 3: Factors associated with the consumption of nutrition supplements**”

Legend: “Figure 3 Demographic and background characteristics (DEMO): GEN-Gender, MS-Marital status, REG-Region, EL-Education level, ES-Employment status, MI-Monthly income per capita (in Saudi riyals "SR "), SMO-Smoking. Health characteristics (HEA): GH-General health, EWC-Weight control program with a dietitian, GFL-General fitness level, PA-Physical activity per week, PS-Professionalism in sport. Knowledge and practices (KP): UNS-Use nutritional supplements, KNS-Knowledge on nutritional supplement ingredients, UCC- Use supplement contain caffeine, EBNS- Effectiveness and benefit of the nutritional supplement, MSNS-Monthly spent on nutritional supplements - (SR ) and *Highly Significant”

**[Page 23, line 613-621]**

**Comment 7:** [We note that Figure 1  in your submission contain map/satellite image which may be copyrighted. All PLOS content is published under the Creative Commons Attribution License (CC BY 4.0), which means that the manuscript, images, and Supporting Information files will be freely available online, and any third party is permitted to access, download, copy, distribute, and use these materials in any way, even commercially, with proper attribution. For these reasons, we cannot publish previously copyrighted maps or satellite images created using proprietary data, such as Google software (Google Maps, Street View, and Earth). For more information, see our copyright guidelines: http://journals.plos.org/plosone/s/licenses-and-copyright.

We require you to either (1) present written permission from the copyright holder to publish these figures specifically under the CC BY 4.0 license, or (2) remove the figures from your submission:

a. You may seek permission from the original copyright holder of Figure 1 to publish the content specifically under the CC BY 4.0 license.

We recommend that you contact the original copyright holder with the Content Permission Form (http://journals.plos.org/plosone/s/file?id=7c09/content-permission-form.pdf) and the following text:
“I request permission for the open-access journal PLOS ONE to publish XXX under the Creative Commons Attribution License (CCAL) CC BY 4.0 (http://creativecommons.org/licenses/by/4.0/). Please be aware that this license allows unrestricted use and distribution, even commercially, by third parties. Please reply and provide explicit written permission to publish XXX under a CC BY license and complete the attached form.”

Please upload the completed Content Permission Form or other proof of granted permissions as an 'Other' file with your submission.

In the figure caption of the copyrighted figure, please include the following text: “Reprinted from [ref] under a CC BY license, with permission from [name of publisher], original copyright [original copyright year].”

b. If you are unable to obtain permission from the original copyright holder to publish these figures under the CC BY 4.0 license or if the copyright holder’s requirements are incompatible with the CC BY 4.0 license, please either i) remove the figure or ii) supply a replacement figure that complies with the CC BY 4.0 license. Please check copyright information on all replacement figures and update the figure caption with source information. If applicable, please specify in the figure caption text when a figure is similar but not identical to the original image and is therefore for illustrative purposes only.
The following resources for replacing copyrighted map figures may be helpful:

USGS National Map Viewer (public domain): http://viewer.nationalmap.gov/viewer/
The Gateway to Astronaut Photography of Earth (public domain): http://eol.jsc.nasa.gov/sseop/clickmap/
Maps at the CIA (public domain): https://www.cia.gov/library/publications/the-world-factbook/index.html and https://www.cia.gov/library/publications/cia-maps-publications/index.html
NASA Earth Observatory (public domain): http://earthobservatory.nasa.gov/
Landsat: http://landsat.visibleearth.nasa.gov/
USGS EROS (Earth Resources Observatory and Science (EROS) Center) (public domain): http://eros.usgs.gov/#
Natural Earth (public domain): http://www.naturalearthdata.com/](https://track.editorialmanager.com/CL0/https:%2F%2Fwww.editorialmanager.com%2Fpone%2Fadmin%2FWe%2520note%2520that%2520Figure%25201%2520%2520in%2520your%2520submission%2520contain%2520map%2Fsatellite%2520image%2520which%2520may%2520be%2520copyrighted.%2520All%2520PLOS%2520content%2520is%2520published%2520under%2520the%2520Creative%2520Commons%2520Attribution%2520License%2520(CC%2520BY%25204.0),%2520which%2520means%2520that%2520the%2520manuscript,%2520images,%2520and%2520Supporting%2520Information%2520files%2520will%2520be%2520freely%2520available%2520online,%2520and%2520any%2520third%2520party%2520is%2520permitted%2520to%2520access,%2520download,%2520copy,%2520distribute,%2520and%2520use%2520these%2520materials%2520in%2520any%2520way,%2520even%2520commercially,%2520with%2520proper%2520attribution.%2520For%2520these%2520reasons,%2520we%2520cannot%2520publish%2520previously%2520copyrighted%2520maps%2520or%2520satellite%2520images%2520created%2520using%2520proprietary%2520data,%2520such%2520as%2520Google%2520software%2520(Google%2520Maps,%2520Street%2520View,%2520and%2520Earth).%2520For%2520more%2520information,%2520see%2520our%2520copyright%2520guidelines:%2520http:%2Fjournals.plos.org%2Fplosone%2Fs%2Flicenses-and-copyright.%250b%250bWe%2520require%2520you%2520to%2520either%2520(1)%2520present%2520written%2520permission%2520from%2520the%2520copyright%2520holder%2520to%2520publish%2520these%2520figures%2520specifically%2520under%2520the%2520CC%2520BY%25204.0%2520license,%2520or%2520(2)%2520remove%2520the%2520figures%2520from%2520your%2520submission:%250b%250ba.%2520You%2520may%2520seek%2520permission%2520from%2520the%2520original%2520copyright%2520holder%2520of%2520Figure%25201%2520to%2520publish%2520the%2520content%2520specifically%2520under%2520the%2520CC%2520BY%25204.0%2520license.%2520%250b%250bWe%2520recommend%2520that%2520you%2520contact%2520the%2520original%2520copyright%2520holder%2520with%2520the%2520Content%2520Permission%2520Form%2520(http:%2Fjournals.plos.org%2Fplosone%2Fs%2Ffile%3Fid=7c09%2Fcontent-permission-form.pdf)%2520and%2520the%2520following%2520text:%250b%E2%80%9CI%2520request%2520permission%2520for%2520the%2520open-access%2520journal%2520PLOS%2520ONE%2520to%2520publish%2520XXX%2520under%2520the%2520Creative%2520Commons%2520Attribution%2520License%2520(CCAL)%2520CC%2520BY%25204.0%2520(http:%2F%2Fcreativecommons.org%2Flicenses%2Fby%2F4.0%2F).%2520Please%2520be%2520aware%2520that%2520this%2520license%2520allows%2520unrestricted%2520use%2520and%2520distribution,%2520even%2520commercially,%2520by%2520third%2520parties.%2520Please%2520reply%2520and%2520provide%2520%23Natural%2520Earth%2520(public%2520domain):%2520http:%2F%2Fwww.naturalearthdata.com%2F8.%2520We%2520notice%2520that%2520your%2520supplementary%2520table%2520is%2520included%2520in%2520the%2520manuscript%2520file.%2520Please%2520remove%2520them%2520and%2520upload%2520them%2520with%2520the%2520file%2520type%2520'Supporting%2520Information'.%2520Please%2520ensure%2520that%2520each%2520Supporting%2520Information%2520file%2520has%2520a%2520legend%2520listed%2520in%2520the%2520manuscript%2520after%2520the%2520references%2520list./1/010f0199318487fa-f3462267-9edc-48f4-b904-a7dc5af6e3cf-000000/vCF4j52FHuZmilGxIPDfJAnUox5YVoZf3b1Pzk4HH78=228)

**Author Response:** Thank you for this observation. We have written R-program and created the map of Saudi Arabia with the longitudes and latitudes of regions from <https://mapcruzin.com/free-saudi-arabia-country-city-place-gis-shapefiles.htm> and is licensed under Creative Commons Attribution Share-Alike 2.0 (CC BY-SA 2.0). Moreover, website is saying that to use without any permission. Not copied elsewhere therefore, we are unable to get permission from the copy right holder and decided to remove the figure 1 from the manuscript.

**Comment 8:** We notice that your supplementary table is included in the manuscript file. Please remove them and upload them with the file type 'Supporting Information'. Please ensure that each Supporting Information file has a legend listed in the manuscript after the references list.

**Author Response:** We agree with the editor observation. Removed the supplementary table from the manuscript and written the caption and legend of the supporting file after the references.

Caption: “**Table S1. Sampling frame and the active individuals**” **[Page 23, line 625-632]**

Legend: “Sample size (n) calculated by the Single proportion (Z2*P*(1-P))/e2. Where Z: Value from standard normal Distribution corresponding to desired confidence level (Z=1.96 for 95% CI), P: Expected true proportione: Desired precision, NR:Non Response Rate, Proportion: Percentage of individuals aged 18 years or above who practice sports activity (150 minutes and more per week) at the dministrative region level (Source:Household Sports Practice Survey 2019, General Authority for Statistics)” **[Page 23, line 625-632]**

**Comment 9:** If the reviewer comments include a recommendation to cite specific previously published works, please review and evaluate these publications to determine whether they are relevant and should be cited. There is no requirement to cite these works unless the editor has indicated otherwise.

**Author Response:** Thanks to the editor for this suggestion. Reviewer-1 suggested three recent, Saudi Arabia country specific studies. Aljebeli et al. (Cureus, 2023), Alotaibi et al. (Saudi Pharmaceutical Journal, 2024), and Alshehri et al. (Nutrients, 2025). We have reviewed and agreed to reviewer 1 suggestion to include and update the literature review and discussion by include in the manuscript. **[Page 2, line 62-68 & Page 13, line 290-291]**

**Additional Editor Comments:**

Thank you for submitting “The consumption of dietary supplements among active individuals in Saudi Arabia” to PLOS ONE. We appreciate the time, effort, and thought you have invested in this important work. Both reviewers recognize that your topic is timely and highly relevant for public health in Saudi Arabia. At the same time, they have identified several key areas—ranging from methods to analysis and presentation—where the manuscript could be strengthened. With thoughtful revisions, your study has the potential to make a meaningful contribution to the field.

**Comment: First,** please update your literature review and discussion to include recent, region-specific studies. Reviewer 1 has pointed to three particularly relevant papers—Aljebeli et al. (Cureus, 2023), Alotaibi et al. (Saudi Pharmaceutical Journal, 2024), and Alshehri et al. (Nutrients, 2025)—that will help sharpen your rationale and place your findings in the context of current Saudi data, including among female fitness-center populations. Integrating these references will also strengthen your interpretation of knowledge and attitude results. Where your findings differ from recent reports, briefly explore possible reasons, such as differences in sampling, setting, or definitions.

**Author Response:** Thanks to the editor for this suggestion. Reviewer-1 suggested three recent, Saudi Arabia country specific studies. Aljebeli et al. (Cureus, 2023), Alotaibi et al. (Saudi Pharmaceutical Journal, 2024), and Alshehri et al. (Nutrients, 2025). We have reviewed and agreed to reviewer 1 suggestion to include and update the literature review and discussion by include in the manuscript. **[Page 2, line 62-68 & Page 13, line 290-291]**

**Comment: Second,** please clarify your sampling approach and discuss its limitations more transparently. While you describe a two-stage cluster design with region-specific targets, actual recruitment was carried out online with the help of volunteers and gym administrators. This may have led to selection bias, favoring gym-affiliated and digitally connected participants, and under-representing others like community sports club members or informal exercisers. To strengthen your methods section, please: (a) explain how you compiled gym lists, which platforms you used, and how many gyms were contacted versus those that participated; (b) report the denominator, response rate, and regional yield; and (c) discuss representativeness in light of the large number of fitness facilities in Saudi Arabia, as Reviewer 1 suggested. If you did not use sampling weights, please update your description to reflect a nonprobability sample and temper your conclusions accordingly. These sampling considerations, along with recall and social desirability bias from self-reporting, should be clearly acknowledged in your Limitations section.

**Author Response:** Thank you for this valuable suggestion. We have rewritten the sampling approach and also updated the limitation section by mentioning the selection bias, favoring gym-affiliated and digitally connected participants, and under-representing others like community sports club members or informal exercisers.

Google Maps or local business listings were used to compile gym lists. The study was limited to gym users. Non-probability sampling method purposive sampling technique was used to ensure the representativeness of gym users from the entire nation. Online questionnaire was distributed through social media-WhatsApp with the help of volunteers, gym administrators. Further, participants were encouraged to recommend the link to other gym users. In total 3800 gym users were included in the study. **[Page 5, line 151-156]**

To the authors’ knowledge, this is the first to assess dietary supplement consumption among active individuals on a national scale. The study has included a wide range of factors to thoroughly examine how they are associated with dietary supplement consumption. However, our study has some limitations. This study cannot establish causality given its cross-sectional nature and limited gym users; this may have led to selection bias and under-representing others like community sports club members or informal exercisers. Furthermore, using a self-reported questionnaire may have introduced recall bias, which, in turn, influenced the results. Future research should focus on examining the accuracy of knowledge related to dietary supplements among active individuals in Saudi Arabia, including their understandings regarding general nutrition, sports nutrition, efficacy of dietary supplements, their potential risks, and guidelines for consumption. The identification of common misconceptions would help inform educational interventions and awareness campaigns intended to enhance knowledge dissemination and informed decision-making about supplement consumption. **[page 17, line 418-442]**

**Comment: Third,** please clarify your definitions and measurements. “Dietary supplements” can mean different things in various studies and policies, and products such as energy drinks, herbal remedies, protein powders, and prescribed supplements are often grouped together. Please provide a clear, authoritative definition (such as from the SFDA or DSHEA), specify exactly what you told participants qualified as a supplement, and break down supplement use by category (vitamins/minerals, protein powders, creatine or other ergogenics, herbal products, energy drinks, physician-prescribed products, and those recommended by dietitians). As Reviewer 2 observed, distinguishing between prescribed/nutritionist-suggested supplements and sports-nutrition products will help readers understand the behaviors you’re reporting.

**Author Response:** Thank you for this valuable observation. The definition of dietary supplement was given by DSHEA and it was stated now in the methods part. **[Page 5 line 135-139]**

The Dietary Supplement Health and Education Act of 1994 defines dietary supplements as a product with one or more nutritional components. Dietary supplements include vitamins, minerals, herbs, meal supplements, sports nutrition products, natural food supplements, and other goods used to supplement dietary ingredients

**Comment: Fourth,** consider streamlining and refocusing your tables for clarity. As Reviewer 2 noted, Tables 1 and 2 repeat information that is later stratified in Tables 3 and 4. You can enhance readability by combining baseline characteristics into a single, compact table and moving extra descriptive details to the Supplement. Main-text tables should directly support your study’s objectives—such as showing prevalence overall and by subgroups, or presenting adjusted associations. For Table 5, which currently mixes “knowledge and practice” items, please either (a) explain why these items are included and how they relate to your aims or (b) move less relevant items to the Supplement and revise the text to align outcomes with objectives. Across all tables, standardize denominators, ensure percentages add up, report exact p-values, and avoid overlapping bivariate tests that could inflate error rates. Focusing your tables this way will make your findings easier to interpret and more impactful.

**Author Response:** Thank you for this valuable observation. We agree with the reviewer 2 suggestion. We have moved Table 1 and Table 2 into supplementary files. The remaining Tables kept in the manuscript.

“Table 1” changed to “Table S2” **[Page 6, line 192]**

“Table 2” changed to “Table S3” **[Page 7, line 199]**

“Table 3” changed to “Table 1” **[Page 8, line 202 & Page 9, line 220 & 221]**

“Table 4” changed to “Table 2” **[Page 10, line 231 & line 233-234]**

“Table 5” changed to “Table 3” **[Page 11, line 244 & line 246-247]**

Titles after the reference written

“**Table S2. Participants background characteristics**” **[Page 23, line 634]**

“**Table S3. Participants health characteristics**” **[Page 24, line 636]**

Table 5 items were used to answer our study one the specific objective, to know the effect of knowledge and practices on the consumption of dietary supplements among active individuals in Saudi Arabia.

Across all tables, standardize denominators, ensure percentages add up, report exact p-values, and avoid overlapping bivariate tests that could inflate error rates. Focusing your tables this way will make your findings easier to interpret and more impactful.

**Comment: Fifth,** please revise your modeling strategy and reporting. Currently, there are inconsistencies in the structural equation modeling (SEM) results—for example, reporting a “non-significant Chi-square” together with a “P-value: 0.000.” Please re-calculate and report fit indices (including χ² with degrees of freedom and p-value, CFI/TLI, RMSEA with 90% CI, and SRMR), using an estimator suited for ordinal data, such as WLSMV. Before conducting path analysis, establish your measurement models for the latent constructs (“knowledge and practices” and “health”) by including factor loadings, reliability (ω/α), and rationale for item inclusion. Please also explain why age and nationality were removed for “low loadings,” since these are demographic variables, not latent factor indicators. If you continue using SEM, clarify what specific research question it addresses, and avoid causal language since your data are cross-sectional. Addressing these modeling issues will help readers trust and understand your analytic approach.

**Author Response:** Thank you for this valuable suggestion. We agreed to the reviewer suggestion and revised SEM modelling strategy with WLSMV and results are updated. In the prior model, demographic factors age and nationality removed from the analysis to improve the model fit since approaching the lower factor loadings. However, in the revised modelling included both age and nationality variables also.

Fig 3 Structural Equation Modelling (SEM) plot indicates that the model with $x^{2}$=3622.475 (d.f=165, P=0.00), Comparative Fit Index (CFI)=0 .746, Root Mean Square Error of Approximation (RMSEA)=0.074 (90% CI: 0.0072-0.076) and SRMR=0.102. The obtained three factors, demographic (DEMO = -0.15), health (HEA= 0.26), and knowledge and practices (KP = 0.86) have highly significant influence on the usage of nutrition supplements among the active individuals, and there was no evidence of collinearity in the model. Factor structures remain intact, and all paths are significant**. [Page 12, line 263-278]**.

**Comment: Sixth,** please make sure your aims, results, and conclusions are all aligned. Reviewer 2 pointed out that your conclusion suggests supplements had effects on endurance, energy, or health—outcomes not directly assessed in your study. If you wish to report participants’ perceptions of such effects, add this explicitly to your Objectives, specify which survey items measured perceived effects, and present those results consistently throughout your manuscript. Otherwise, revise your Abstract, Discussion, and Conclusion to keep the focus on prevalence, patterns, and correlates of supplement use, rather than on any effects.

**Author Response:**

**Comment: Seventh,** please strengthen your documentation of the survey instrument and data transparency. While you mention that the questionnaire was “self-developed” and “adapted from validated questionnaires,” it’s important to detail how you adapted and translated the instrument, including any pilot testing or psychometric evaluation. Please provide the original sources, describe the adaptation and translation/back-translation steps, and summarize cognitive testing and reliability metrics for each scale. Consider including item wording in a Supplement. Also, per PLOS ONE requirements, make the de-identified dataset and code available in a stable repository with DOIs at the time of revision, and update your Data Availability Statement accordingly. Finally, please confirm that your funding statement makes clear the funder’s role (or lack thereof) in study design, analysis, and publication decisions, and that your ethics and consent reporting matches both the Methods and submission system.

**Author Response:**

**Questionnaire:** Pilot study was conducted and final version of the questionnaire was approved by the National Nutrition Committee of Saudi Food and Drug Administration (SFDA). **[Page 3, line 93-94]**

**Data Availability:** Supporting file “Raw data Excel file [V1 (29092025 - V8_NS).xlsx]” uploaded to replicate and assess the accuracy of the results by publishing authority. Requesting the editor to use sheet-4 of the file.

**Updated funding statement:** “Princess Nourah Bint Abdulrahman University Researchers Supporting Project number (PNURSP2025R207), Riyadh, Saudi Arabia.” Mentioned in cover letter also

**Funder’s role in the study:** “The funders had no role in study design, data collection and analysis, decision to publish, or preparation of the manuscript.” Mentioned in the cover letter also.

**Comment: Eighth,** your manuscript would benefit from careful language and presentation polishing. There are some recurring grammatical errors (e.g., “predectors,” “demonistrated”), spacing and capitalization inconsistencies, rounding issues, and a few contradictions between text and figures. We recommend a professional English-language edit and a final technical review to ensure consistency in your data and presentation. These steps will help your work read smoothly and appear as strong as possible.

**Author Response: Thank you for this recommendation. Proof reading certificate attached as supporting file.**

**Comment: In your rebuttal,** in addition to responding to both reviewers’ comments point by point, please also include: (1) a clear plan for which tables will stay in the main text versus the Supplement (and your reasoning); (2) a precise operational definition of “dietary supplement” as used in your survey, along with updated, category-specific results; (3) corrected and fully reported SEM/CFA results (or your rationale for removing these analyses, if you choose to focus on descriptive or associational findings); (4) an expanded Limitations section, including discussion of selection and coverage bias from your online, gym-based recruitment and whether sampling weights were used; and (5) updated references from 2023–2025, including but not limited to the three key works suggested by Reviewer 1.

**Author Response:**

1. A clear plan for which tables will stay in the main text versus the Supplement (and your reasoning);

Table 1 and Table 2 moved into supplementary files. The remaining Tables kept in the manuscript.

“Table 1” changed to “Table S2” **[Page 6, line 191-192]**

“Table 2” changed to “Table S3” **[Page 7, line 199]**

“Table 3” changed to “Table 1” **[Page 8, line 202 & Page 9, line 220-221]**

“Table 4” changed to “Table 2” **[Page 10, line 231 & Page10, line 223-224]**

“Table 5” changed to “Table 3” **[Page 11, line 244 & Page 11, line 246-247]**

Supplementary table titles after the reference written

“**Table S2. Participants background characteristics**” **[Page 23, line 634]**

“**Table S3. Participants health characteristics**” **[Page 24, line 636]**

Table 5 items were used to answer our study one the specific objective, to know the effect of knowledge and practices on the consumption of dietary supplements among active individuals in Saudi Arabia.

(2) A precise operational definition of “dietary supplement” as used in your survey, along with updated, category-specific results;

**Author Response:**

Definition added. **[Page 5, line 135-139]**

(3) Corrected and fully reported SEM/CFA results (or your rationale for removing these analyses, if you choose to focus on descriptive or associational findings)

Fig 3 Structural Equation Modelling (SEM) plot indicates that the model with $x^{2}$=3622.475 (d.f=165, P=0.00), Comparative Fit Index (CFI)=0 .746, Root Mean Square Error of Approximation (RMSEA)=0.074 (90% CI: 0.0072-0.076) and SRMR=0.102. The obtained three factors, demographic (DEMO = -0.15), health (HEA= 0.26), and knowledge and practices (KP = 0.86) have highly significant influence on the usage of nutrition supplements among the active individuals, and there was no evidence of collinearity in the model. Factor structures remain intact, and all paths are significant**. [Page 12, line 263-278].**

(4) an expanded Limitations section, including discussion of selection and coverage bias from your online, gym-based recruitment and whether sampling weights were used;

**Author Response:**

To the authors’ knowledge, this is the first to assess dietary supplement consumption among active individuals on a national scale. The study has included a wide range of factors to thoroughly examine how they are associated with dietary supplement consumption. However, our study has some limitations. This study cannot establish causality given its cross-sectional nature and limited gym users; this may have led to selection bias and under-representing others like community sports club members or informal exercisers. Furthermore, using a self-reported questionnaire may have introduced recall bias, which, in turn, influenced the results. Future research should focus on examining the accuracy of knowledge related to dietary supplements among active individuals in Saudi Arabia, including their understandings regarding general nutrition, sports nutrition, efficacy of dietary supplements, their potential risks, and guidelines for consumption. The identification of common misconceptions would help inform educational interventions and awareness campaigns intended to enhance knowledge dissemination and informed decision-making about supplement consumption. **[page 17, line 418-442]**

(5) Updated references from 2023–2025, including but not limited to the three key works suggested by Reviewer 1.

**Author Response: Three references 14, 15, and 16 were added [page 19-20, line 506-515]**

We look forward to receiving your thoroughly revised manuscript, along with a detailed response to reviewers and tracked-changes files. This decision is based on the version you submitted and the peer reviews provided. Thank you again for your hard work and commitment to improving your study.

**Reviewers' comments:
Reviewer's Responses to Questions**

**Comments to the Author**

1. Is the manuscript technically sound, and do the data support the conclusions?
The manuscript must describe a technically sound piece of scientific research with data that supports the conclusions. Experiments must have been conducted rigorously, with appropriate controls, replication, and sample sizes. The conclusions must be drawn appropriately based on the data presented.

Reviewer #1: Yes

Reviewer #2: Partly

2. Has the statistical analysis been performed appropriately and rigorously?

Reviewer #1: Yes

Reviewer #2: Yes

3. Have the authors made all data underlying the findings in their manuscript fully available?

The [PLOS Data policy](http://track.editorialmanager.com/CL0/http:%2F%2Fwww.plosone.org%2Fstatic%2Fpolicies.action%23sharing/1/010f0199318487fa-f3462267-9edc-48f4-b904-a7dc5af6e3cf-000000/MYxjZYA9NkQk8KwW1obDA17E1zejQOn1-Kgg9HVBL9I=228) requires authors to make all data underlying the findings described in their manuscript fully available without restriction, with rare exception (please refer to the Data Availability Statement in the manuscript PDF file). The data should be provided as part of the manuscript or its supporting information, or deposited to a public repository. For example, in addition to summary statistics, the data points behind means, medians and variance measures should be available. If there are restrictions on publicly sharing data—e.g. participant privacy or use of data from a third party—those must be specified.

Reviewer #1: Yes

Reviewer #2: No

4. Is the manuscript presented in an intelligible fashion and written in standard English?

PLOS ONE does not copyedit accepted manuscripts, so the language in submitted articles must be clear, correct, and unambiguous. Any typographical or grammatical errors should be corrected at revision, so please note any specific errors here.

Reviewer #1: Yes

Reviewer #2: Yes

5. Review Comments to the Author

Please use the space provided to explain your answers to the questions above. You may also include additional comments for the author, including concerns about dual publication, research ethics, or publication ethics. (Please upload your review as an attachment if it exceeds 20,000 characters)

Reviewer #1: This manuscript investigates the prevalence and patterns of dietary supplement consumption among physically active individuals in Saudi Arabia. This is an interesting topic given the increasing popularity of supplements and limited data in this region.
I have few recommendations
more updated references should be cited
1) Aljebeli, Shahad et al. “The Prevalence and Awareness of Dietary Supplement Use Among Saudi Women Visiting Fitness Centers in Riyadh, Saudi Arabia.” Cureus vol. 15,6 e41031. 27 Jun. 2023, doi:10.7759/cureus.41031
2) Alotaibi, Abdulkarim F et al. “Pattern of dietary supplement use and its psychosocial predictors among females visiting fitness centers in Saudi Arabia: Findings from a cross-sectional study.” Saudi pharmaceutical journal : SPJ : the official publication of the Saudi Pharmaceutical Society vol. 32,3 (2024): 101966. doi:10.1016/j.jsps.2024.101966
3) Alshehri, A.A.; Alqahtani, S.; Aldajani, R.; Alsharabi, B.; Alzahrani, W.; Alguthami, G.; Khawagi, W.Y.; Arida, H. Knowledge, Attitudes, and Practices of Dietary Supplement Use in Western Saudi Arabia: A Cross-Sectional Study. Nutrients 2025, 17, 1233. <https://doi.org/10.3390/nu17071233>

**Author Response:** Thank you for the valuable recommendation, We have included and update the literature review and discussion in the manuscript.

I think more limitation should be addressed such as
selection bias as the questionnaire was distributed online though gym administration taking into account that there is around 3,966 fitness gym is KSA. Participants may be recruited from gyms, fitness centers, or sports clubs, which may not represent all active Saudis.
Participants may include herbal products, energy drinks, protein powders, or even medications under "supplements," which makes comparisons difficult and inn the study there was clarification about supplements used by the participants

Updated Limitation section: To the authors’ knowledge, this is the first to assess dietary supplement consumption among active individuals on a national scale. The study has included a wide range of factors to thoroughly examine how they are associated with dietary supplement consumption. However, our study has some limitations. This study cannot establish causality given its cross-sectional nature and limited gym users; this may have led to selection bias and under-representing others like community sports club members or informal exercisers. Furthermore, using a self-reported questionnaire may have introduced recall bias, which, in turn, influenced the results. Future research should focus on examining the accuracy of knowledge related to dietary supplements among active individuals in Saudi Arabia, including their understandings regarding general nutrition, sports nutrition, efficacy of dietary supplements, their potential risks, and guidelines for consumption. The identification of common misconceptions would help inform educational interventions and awareness campaigns intended to enhance knowledge dissemination and informed decision-making about supplement consumption. **[page 17, line 418-442]**

Reviewer #2: Tables 1 & 2 are additional, while these characteristics are already presented in Tables 3 & 4 with more details in relation to the use or non-use of nutritional supplements. There is information about the specific use or consumption of nutritional supplements, including vitamins, herbs etc. and further it can be categorized with other health characteristics, etc.
Data presented in table 5 related to the knowledge and practice in relation to consumption of nutritional supplements is irrelevant and not in line with the rationale of the study.
Conclusion of study is also not in accordance with the findings/results, as the effect of nutritional supplements is not the objective of study. the perception of participants about their effect on their health should be added in the objectives and detailed in the results and discussion part.

**Author response:**

Table 1 and Table 2 moved into supplementary files. The remaining Tables kept in the manuscript.

“Table 1” changed to “Table S2” **[Page 6, line 192]**

“Table 2” changed to “Table S3” **[Page 7, line 199]**

“Table 3” changed to “Table 1” **[Page 8, line 202 & Page 9, line 220 & 221]**

“Table 4” changed to “Table 2” **[Page 10, line 231 & line 233-234]**

“Table 5” changed to “Table 3” **[Page 11, line 244 & line 246-247]**

Supplementary table titles after the reference written

“**Table S2. Participants background characteristics**” **[Page 23, line 634]**

“**Table S3. Participants health characteristics**” **[Page 24, line 636]**

Table 5 items were used to answer our study one the specific objective, to know the effect of knowledge and practices on the consumption of dietary supplements among active individuals in Saudi Arabia.

This study aimed to estimate the prevalence for the consumption of dietary supplements, to assess the perceptions about the use of dietary supplements effect on health and to identify the factors like demographic, health, knowledge and practices effecting the consumption of dietary supplements among active individuals in Saudi Arabia. **[Page 1, line 25-31 & page 3 line 83-86]**

6. PLOS authors have the option to publish the peer review history of their article ([what does this mean?](https://track.editorialmanager.com/CL0/https:%2F%2Fjournals.plos.org%2Fplosone%2Fs%2Feditorial-and-peer-review-process%23loc-peer-review-history/1/010f0199318487fa-f3462267-9edc-48f4-b904-a7dc5af6e3cf-000000/NNP83lLOeZ_484Q7P9cq-AakI-ikgE-c2MHiDZYcBwE=228)). If published, this will include your full peer review and any attached files.

If you choose “no”, your identity will remain anonymous but your review may still be made public.

Do you want your identity to be public for this peer review? For information about this choice, including consent withdrawal, please see our [Privacy Policy](https://track.editorialmanager.com/CL0/https:%2F%2Fwww.plos.org%2Fprivacy-policy/1/010f0199318487fa-f3462267-9edc-48f4-b904-a7dc5af6e3cf-000000/IuwyHIo71URLI2WS8fzzO_6YByJpwlNDIvLHkQC1MOw=228).

Reviewer #1: Yes: Khloud Ghafouri

Reviewer #2: Yes: Intisar Ahmad Siddiqui

[NOTE: If reviewer comments were submitted as an attachment file, they will be attached to this email and accessible via the submission site. Please log into your account, locate the manuscript record, and check for the action link "View Attachments". If this link does not appear, there are no attachment files.]

While revising your submission, please upload your figure files to the Preflight Analysis and Conversion Engine (PACE) digital diagnostic tool, [https://pacev2.apexcovantage.com/](https://track.editorialmanager.com/CL0/https:%2F%2Fpacev2.apexcovantage.com%2F/1/010f0199318487fa-f3462267-9edc-48f4-b904-a7dc5af6e3cf-000000/cUGMq58ft76FCbbsN44vcE88lvALvng3WMeHqqn5eq4=228). PACE helps ensure that figures meet PLOS requirements. To use PACE, you must first register as a user. Registration is free. Then, login and navigate to the UPLOAD tab, where you will find detailed instructions on how to use the tool. If you encounter any issues or have any questions when using PACE, please email PLOS at [figures@plos.org](mailto:figures@plos.org). Please note that Supporting Information files do not need this step.
