# Supplementary material for: Dietary supplement consumption among active individuals in Saudi Arabia
Source: PLoS One. 2026 Jun 22;21(6):e0351208. doi: 10.1371/journal.pone.0351208 (PMC13286177; doi:10.1371/journal.pone.0351208)
Supplement: Attachment — Submitted filename: Response_to_Reviewers_auresp_3.docx [file pone.0351208.s010.docx]

**Response to Additional Editor Comments - PLOS ONE**

**The consumption of dietary supplements among active individuals in Saudi Arabia**
**Manuscript ID: PONE-D-25-22005R2**

Dear Academic Editor,

Jordan Llego, PhD ELM, D. Hon. Ex., PhDN, RN.

Thank you for the opportunity to revise our manuscript and for the insightful comments provided by you. We appreciate the time and effort invested in evaluating our work. We have carefully considered all feedback and revised the manuscript accordingly. Below, we provided a detailed point-by-point response to each comment.

**Comment 1:** First, while the structural equation modeling section is now clearer, some concerns remain about the measurement model’s robustness and reporting. Specifically, the relatively low factor-loading threshold, borderline fit indices, and the inclusion of a demographic latent construct with a non-significant path need clearer justification. You might want to strengthen your reporting of the CFA and reliability for each construct, or consider simplifying your analytical approach if that makes sense. Addressing this will help readers have more confidence in your model and its interpretation.

**Author Response:** Thanks to the editor for this valuable suggestion. We have increased the factor-loading threshold from 0.20 to 0.40. Consequently, CFI was increased from 0.901 to 0.953. The latent variables, use supplements contain caffeine (UCC), use nutritional supplements (UNS) from knowledge and practices (KP) domain, items; weight control program with a dietitian (EWC), general health (GH) from health characteristics (HEA) domain and the items smoking (SMO), employment status (ES), education level (EL), region (REG) from Demographic and background characteristics (DEMO) domain were not met the increased factor loading threshold 0.40 and removed from further path way analysis (SEM) plot. The demographic construct was considered to test its theoretical relevance and to demonstrate that demographic characteristics did not materially influence supplement use in the presence of health and knowledge factors. Reporting this non-significant pathway provides transparency and supports the interpretation that health and knowledge-related domains are more salient determinants.

**Comment 2:** Second, the data availability statement, though expanded, may still need more clarity for full compliance with PLOS ONE policy. Please clearly state the ethical or legal reasons for restricting public access to the dataset and make sure they align with your institution’s regulations. If possible, let us know whether a de-identified or partial dataset could be shared, or clarify exactly under what conditions data access can be granted.

**Author Response:** Thanks to the editor for this valuable comment, we have updated the data availability statement. ‘Anonymised data will be available in supporting information files’ (Data Set: NS_CLN_Data_Supporting_File.xlsx)

**Comment 3:** Third, your manuscript would benefit from a careful language edit. While your content is generally clear, there are still a few grammatical errors, typos, and minor inconsistencies in terminology to address for a polished, professional presentation.

**Author Response:** Thank you for this recommendation. We have made language and grammar check by a professional proofreading company “editage” and the certificate was attached as supporting file.
