# Supplementary material for: Dietary supplement consumption among active individuals in Saudi Arabia
Source: PLoS One. 2026 Jun 22;21(6):e0351208. doi: 10.1371/journal.pone.0351208 (PMC13286177; doi:10.1371/journal.pone.0351208)
Supplement: Attachment — Submitted filename: Response to Reviewers 5.docx [file pone.0351208.s011.docx]

**Response to Additional Editor Comments - PLOS ONE**

**Dietary supplement consumption among active individuals in Saudi Arabia**
**Manuscript ID: [PONE-D-25-22005R3]-[EMID:30830b9183d50455]**

Dear Academic Editor,

Jordan Llego, PhD ELM, D. Hon. Ex., PhDN, RN.

We sincerely thank the Editor for the opportunity to revise our manuscript and for the insightful comments provided. We appreciate the time and effort dedicated to evaluating our work. All feedback has been carefully considered, and the manuscript has been revised accordingly. Below is a detailed, point-by-point response to each of the comments.

**Journal Requirements**

**Comment 1:** If the reviewer comments include a recommendation to cite specific previously published works, please review and evaluate these publications to determine whether they are relevant and should be cited. There is no requirement to cite these works unless the editor has indicated otherwise.

**Author Response:** We thank the Editor for this suggestion. However, as this is the fifth revision and we have not received specific recommendations from the reviewers to incorporate additional references at this stage, we have maintained the current list to preserve the focus of the study.

**Comment 2:** Please review your reference list to ensure that it is complete and correct. If you have cited papers that have been retracted, please include the rationale for doing so in the manuscript text, or remove these references and replace them with relevant current references. Any changes to the reference list should be mentioned in the rebuttal letter that accompanies your revised manuscript. If you need to cite a retracted article, indicate the article’s retracted status in the References list and also include a citation and full reference for the retraction notice.

**Author Response:** We appreciate the Editor's emphasis on the accuracy of the reference list. We have conducted a comprehensive review of all citations to ensure they are complete, accurate, and strictly formatted according to the journal’s guidelines. Furthermore, we have verified the status of all cited works and confirm that no retracted articles are included in this manuscript. No substantive changes were deemed necessary during this verification process.

**Additional Editor Comments**

**Comment 3:** Please double-check that your Data Availability statement in the manuscript matches what you’ve selected in the submission system. Right now, the manuscript says an anonymized dataset is available in the Supporting Information, but the submission metadata suggests there may be restrictions—these need to be consistent. If the data are fully available, please say so clearly in both places and indicate there are no restrictions. If there are restrictions, please explain the ethical or legal reasons, say who can be contacted for data access, and clarify exactly what can be shared and under what circumstances.

**Author Response:** We thank the Editor for this important observation. Please note that the option to modify this section in the online submission system is no longer accessible at this stage of the revision process. However, we would like to clarify that the data availability remains as stated in the manuscript: **“Anonymized data will be available in the supporting information files”** (Data Set: NS_CLN_Data_Supporting_File.xlsx). We have ensured that this file is correctly uploaded and properly cited within the text to maintain full transparency.

**Comment 4:** Also, please do a final check to make sure that ethics approval numbers, funding information, institutional names, and any reference codes are listed the same way throughout your manuscript and submission materials. Although the writing is much improved, it’s a good idea to do one last careful edit for typos, consistent use of terms and abbreviations, and to make sure all supplementary files are correctly named, uploaded, and referenced in the text.

**Author Response:** Thank you for this constructive suggestion. We have performed a rigorous final review of the entire manuscript and submission materials to ensure absolute consistency. All ethical approval numbers, funding details, institutional affiliations, and reference codes have been cross-verified for accuracy. Additionally, we have conducted a final linguistic and formatting edit to eliminate any typographical errors and ensure the consistent use of terminology and abbreviations. All supplementary files have been verified for correct naming and proper citation within the text. We would like to express our gratitude to the Editor once again for the opportunity to further refine and improve the quality of our manuscript.

**Changes and line numbers in the file:** Revised Manuscript with Track Changes

Line 106-107: Saudi Arabia currency symbol () added. “level-“changed to “level ()”

Line 123: Saudi Arabia currency symbol () added. “dietary supplements ()?” changed to “dietary supplements ()?

Line 124: “>1,500” changed to “≥1,500”

Line 157: Deleted “see”

Line 195: Saudi Arabia currency symbol () added. “monthly income of <2000 ()” changed to “monthly income () <2000”

Line 196: Deleted “see”

Line 200: Deleted “see”

Line 213: Saudi Arabia currency symbol () added. “monthly income ()” changed to “monthly income ()”

Line 215: Saudi Arabia currency symbol () added. “Those who spent ()” changed to “Those who spent ()”

Line 235: Deleted “see”

Line 244: “S1 Fig” changed to “Figure S1”

Line 250: Saudi Arabia currency symbol () added. “participants spent <500 ()” changed to “participants spent () <500”

Line 251: “500-1,000 ()” changed to “500-1,000” and deleted “see”

Line 258: “Fig1A” changed to “Figure-A”

Line 260: “Fig1B” changed to “Figure-B”

Line 262: “Fig1C” changed to “Figure-C”

Line 272: Deleted “were examined”

Line 274: “-“ changed to “;” and S2 Fig” changed to “Figure S2”

Line 276: Full form provided. “SEM” changed to “Sequence Equation Modelling (SEM)”

Line 279: Deleted “_”

Line 282: “Fig3” changed to “Figure 3”

**Tables:**

Line 223: Table 1; Variable Marital Status, sub-heading “Single” changed to “Single/Unmarried”

Line 223: Table 1; Variable “Income level (SR)” changed to “Income level ()”

Line 223: Table 1; Variable “Money spent on NS monthly (SR)” changed to “Money spent on NS monthly ()”

Line 224: Table 1: Below the table annotate added “Nutrition Supplement (NS)”

Line 238: Table 2; Variable “<150 min/WK” changed <150 min/wk” and “≥150 min/WK” changed to “≥150 min/wk”

Line 255: Table 3; Last variable “(SR)” changed to “()” ; “<500 –“ changed to “<500”; “500-<1000-“ changed to “500-1000”; “1000-<1500 –“ changed to “1000-1500”; and >1500 –“ changed to “≥1500”
