# Supplementary material for: Dietary supplement consumption among active individuals in Saudi Arabia
Source: PLoS One. 2026 Jun 22;21(6):e0351208. doi: 10.1371/journal.pone.0351208 (PMC13286177; doi:10.1371/journal.pone.0351208)
Supplement: Attachment — Submitted filename: Response to Reviewers 5-26.docx [file pone.0351208.s012.docx]

**Response to Editor Comments - PLOS ONE**

**Dietary supplement consumption among active individuals in Saudi Arabia**
**Manuscript ID: [PONE-D-25-22005R4] - [EMID:55a0a1d72bb1cee3]**

Dear Academic Editor,

Jordan Llego, PhD ELM, D. Hon. Ex., PhDN, RN.

We sincerely thank to the Editor for your thorough and constructive assessment of our manuscript. We have carefully addressed all points raised and provide detailed responses below. All changes are highlighted in the accompanying Revised Manuscript with Track Changes.

**Editor's Comments and Authors' Responses**

**Editor's Comment 1 - Internal consistency of demographic findings:** The manuscript does not present a coherent interpretation of the role of demographic factors. In the abstract, demographic factors are reported as not exhibiting a significant effect. In the Results section, several demographic variables in the bivariate analysis are reported as statistically significant. In the SEM section, the demographic domain is described as non-significant. In the Conclusion, sociodemographic factors are again described as significantly associated with dietary supplement consumption. These statements are not interchangeable and should not be presented together without clear explanation. It is essential to distinguish between variables significant in the bivariate analysis and those remaining significant in the structural model, and this distinction must be maintained consistently across the Abstract, Results, Discussion, and Conclusion.

**Author Response:** We thank the Editor for identifying this critical inconsistency. We fully agree that the distinction between bivariate and structural findings must be explicitly and consistently maintained throughout the manuscript. We have made the following targeted revisions:

(a) Abstract - Results: The phrase "demographic factors did not exhibit a significant effect" has been replaced with: "the demographic domain did not show a significant association in the structural equation model, although several demographic variables were significantly associated with supplement use in bivariate analysis." This wording now accurately reflects the layered nature of the findings.

(b) Conclusion: The statement "Sociodemographic factors - including gender, employment status, income level, and education - were significantly associated with dietary supplement consumption" has been revised to: "In bivariate analysis, sociodemographic factors - including gender, employment status, income level, and education - were significantly associated with dietary supplement consumption; however, the demographic domain did not retain significance in the structural equation model." This ensures readers clearly understand the level of analysis at which each association was observed.

The Results and Discussion sections already correctly describe both the bivariate (Table 1) and SEM findings separately, and these sections have been reviewed to confirm they remain consistent with the above changes.

**Editor's Comment 2 - Inconsistent description of the questionnaire instrument:** In the abstract, the questionnaire is described as a self-developed Likert-scale instrument, while in the Methods section it is characterized as adapted from validated local and international tools with minor modifications. This inconsistency should be resolved to ensure a single, accurate, and consistent description of the instrument.

**Author Response:** We apologize for this inconsistency. The Methods section accurately describes the instrument as adapted from validated local and international tools with minor modifications to ensure cultural relevance. The Abstract has been corrected accordingly. The phrase "self-developed Likert-scale questionnaire" has been replaced with "Likert-scale questionnaire adapted from validated local and international tools with minor modifications," bringing the Abstract into full alignment with the Methods section.

**Editor's Comment 3 - SEM model fit criteria and interpretation:** The manuscript states that good model fit is indicated by RMSEA <0.05, CFI >=0.92, and a non-significant chi-squared statistic, yet the reported SEM results do not fully meet these criteria. Additionally, the manuscript claims that construct validity was supported by CFA, although the reported fit indices are only modest rather than unequivocally strong.

**Author Response:** We thank the Editor for this important methodological observation. The reported SEM indices (RMSEA = 0.070, CFI = 0.953, SRMR = 0.082) do not meet the stricter thresholds of RMSEA <0.05 and CFI >=0.92 stated in the original Statistical Analysis section. We have therefore revised the stated thresholds to those that the model actually satisfies: RMSEA <0.08 and CFI >=0.90, which are widely accepted as indicators of acceptable fit in structural equation modeling (Hu & Bentler, 1999; Browne & Cudeck, 1993). The phrase regarding chi-squared non-significance has also been revised, as a significant chi-squared statistic is expected with large samples and is not informative as a standalone criterion.

Regarding construct validity, the CFA fit indices (CFI = 0.901, RMSEA = 0.072) support acceptable rather than strong construct validity. The relevant text has been reviewed to ensure the claim is appropriately qualified and does not overstate the evidence.

**Editor's Comment 4 - Currency notation and formatting issues:** Visible formatting and presentation issues persist, including unresolved currency notation and wording that indicate the manuscript has not undergone a thorough final proofread. Terminology, symbols, table labels, and sentence-level wording should be fully standardized throughout the revised manuscript.

**Author Response:** We sincerely apologize for these presentation issues. A thorough editorial review has been conducted across the full manuscript. Specifically:

(a) Currency notation: All instances of the unresolved Saudi Riyal symbol - appearing as an embedded image or unrendered character - have been replaced uniformly with the standard abbreviation "SAR" throughout the manuscript, including the Methods, Results, Tables, and all figure/supplementary captions.

(b) General proofreading: Sentence-level wording, table labels, and terminology have been reviewed and standardized throughout the manuscript. We are confident the revised manuscript now meets the journal presentation standards.

We hope that the revised manuscript now satisfactorily addresses all concerns raised. We remain available to provide any further clarification required.

Yours sincerely,

Abdulrahman A. Alsayegh, on behalf of all co-authors

Corresponding Author: aalsayegh@jazanu.edu.sa
