# Supplementary material for: Dietary supplement consumption among active individuals in Saudi Arabia
Source: PLoS One. 2026 Jun 22;21(6):e0351208. doi: 10.1371/journal.pone.0351208 (PMC13286177; doi:10.1371/journal.pone.0351208)
Supplement: Peer review history

# Peer review: Decision Letter 0

**DOI:** 10.1371/journal.pone.0351208.r001

**Authors:** Jordan Llego

9 Sep 2025

PONE-D-25-22005

The consumption of dietary supplements among active individuals in Saudi Arabia

PLOS ONE

Dear Dr. Alsayegh,

Thank you for submitting your manuscript to PLOS ONE. After careful consideration, we feel that it has merit but does not fully meet PLOS ONE’s publication criteria as it currently stands. Therefore, we invite you to submit a revised version of the manuscript that addresses the points raised during the review process.

Please submit your revised manuscript by Oct 24 2025 11:59PM. If you will need more time than this to complete your revisions, please reply to this message or contact the journal office at plosone@plos.org. When you're ready to submit your revision, log on to https://www.editorialmanager.com/pone/ and select the 'Submissions Needing Revision' folder to locate your manuscript file.

Please include the following items when submitting your revised manuscript:

- A rebuttal letter that responds to each point raised by the academic editor and reviewer(s). You should upload this letter as a separate file labeled 'Response to Reviewers'.
- A marked-up copy of your manuscript that highlights changes made to the original version. You should upload this as a separate file labeled 'Revised Manuscript with Track Changes'.
- An unmarked version of your revised paper without tracked changes. You should upload this as a separate file labeled 'Manuscript'.

If you would like to make changes to your financial disclosure, please include your updated statement in your cover letter. Guidelines for resubmitting your figure files are available below the reviewer comments at the end of this letter.

If applicable, we recommend that you deposit your laboratory protocols in protocols.io to enhance the reproducibility of your results. Protocols.io assigns your protocol its own identifier (DOI) so that it can be cited independently in the future. For instructions see: https://journals.plos.org/plosone/s/submission-guidelines#loc-laboratory-protocols. Additionally, PLOS ONE offers an option for publishing peer-reviewed Lab Protocol articles, which describe protocols hosted on protocols.io. Read more information on sharing protocols at https://plos.org/protocols?utm_medium=editorial-email&utm_source=authorletters&utm_campaign=protocols.

We look forward to receiving your revised manuscript.

Kind regards,

Jordan Llego, PhD ELM, D. Hon. Ex., PhDN, RN

Academic Editor

PLOS ONE

Journal Requirements:

When submitting your revision, we need you to address these additional requirements.

1. Please ensure that your manuscript meets PLOS ONE's style requirements, including those for file naming. The PLOS ONE style templates can be found at

https://journals.plos.org/plosone/s/file?id=wjVg/PLOSOne_formatting_sample_main_body.pdf and

https://journals.plos.org/plosone/s/file?id=ba62/PLOSOne_formatting_sample_title_authors_affiliations.pdf

2. Please provide additional details regarding participant consent. In the ethics statement in the Methods and online submission information, please ensure that you have specified (1) whether consent was informed and (2) what type you obtained (for instance, written or verbal, and if verbal, how it was documented and witnessed). If your study included minors, state whether you obtained consent from parents or guardians. If the need for consent was waived by the ethics committee, please include this information.

If you are reporting a retrospective study of medical records or archived samples, please ensure that you have discussed whether all data were fully anonymized before you accessed them and/or whether the IRB or ethics committee waived the requirement for informed consent. If patients provided informed written consent to have data from their medical records used in research, please include this information.

3. Thank you for stating the following financial disclosure:

“Princess Nourah Bint Abdulrahman University Researchers Supporting Project number (PNURSP2025R207).”

Please state what role the funders took in the study. If the funders had no role, please state: 'The funders had no role in study design, data collection and analysis, decision to publish, or preparation of the manuscript.'

If this statement is not correct you must amend it as needed.

Please include this amended Role of Funder statement in your cover letter; we will change the online submission form on your behalf.

4. Thank you for stating the following in the Acknowledgments Section of your manuscript:

“the authors would like to acknowledge and appreciate all participants for their cooperation in filling out the survey to complete this study, in addition to deep grateful for Princess Nourah bint Abdulrahman University for funding and supporting this work.”

We note that you have provided funding information that is currently declared in your Funding Statement. However, funding information should not appear in the Acknowledgments section or other areas of your manuscript. We will only publish funding information present in the Funding Statement section of the online submission form.

Please remove any funding-related text from the manuscript and let us know how you would like to update your Funding Statement. Currently, your Funding Statement reads as follows:

“Princess Nourah Bint Abdulrahman University Researchers Supporting Project number (PNURSP2025R207).”

Please include your amended statements within your cover letter; we will change the online submission form on your behalf.

5. We note that your Data Availability Statement is currently as follows: All relevant data are within the manuscript and its Supporting Information files.

Please confirm at this time whether or not your submission contains all raw data required to replicate the results of your study. Authors must share the “minimal data set” for their submission. PLOS defines the minimal data set to consist of the data required to replicate all study findings reported in the article, as well as related metadata and methods (https://journals.plos.org/plosone/s/data-availability#loc-minimal-data-set-definition).

For example, authors should submit the following data:

- The values behind the means, standard deviations and other measures reported;

- The values used to build graphs;

- The points extracted from images for analysis.

Authors do not need to submit their entire data set if only a portion of the data was used in the reported study.

If your submission does not contain these data, please either upload them as Supporting Information files or deposit them to a stable, public repository and provide us with the relevant URLs, DOIs, or accession numbers. For a list of recommended repositories, please see https://journals.plos.org/plosone/s/recommended-repositories.

If there are ethical or legal restrictions on sharing a de-identified data set, please explain them in detail (e.g., data contain potentially sensitive information, data are owned by a third-party organization, etc.) and who has imposed them (e.g., an ethics committee). Please also provide contact information for a data access committee, ethics committee, or other institutional body to which data requests may be sent. If data are owned by a third party, please indicate how others may request data access.

6. Please include a separate caption for each figure in your manuscript.

7. We note that Figure 1  in your submission contain map/satellite image which may be copyrighted. All PLOS content is published under the Creative Commons Attribution License (CC BY 4.0), which means that the manuscript, images, and Supporting Information files will be freely available online, and any third party is permitted to access, download, copy, distribute, and use these materials in any way, even commercially, with proper attribution. For these reasons, we cannot publish previously copyrighted maps or satellite images created using proprietary data, such as Google software (Google Maps, Street View, and Earth). For more information, see our copyright guidelines: http://journals.plos.org/plosone/s/licenses-and-copyright.

We require you to either (1) present written permission from the copyright holder to publish these figures specifically under the CC BY 4.0 license, or (2) remove the figures from your submission:

a. You may seek permission from the original copyright holder of Figure 1 to publish the content specifically under the CC BY 4.0 license.

We recommend that you contact the original copyright holder with the Content Permission Form (http://journals.plos.org/plosone/s/file?id=7c09/content-permission-form.pdf) and the following text:

“I request permission for the open-access journal PLOS ONE to publish XXX under the Creative Commons Attribution License (CCAL) CC BY 4.0 (http://creativecommons.org/licenses/by/4.0/). Please be aware that this license allows unrestricted use and distribution, even commercially, by third parties. Please reply and provide explicit written permission to publish XXX under a CC BY license and complete the attached form.”

Please upload the completed Content Permission Form or other proof of granted permissions as an 'Other' file with your submission.

In the figure caption of the copyrighted figure, please include the following text: “Reprinted from [ref] under a CC BY license, with permission from [name of publisher], original copyright [original copyright year].”

b. If you are unable to obtain permission from the original copyright holder to publish these figures under the CC BY 4.0 license or if the copyright holder’s requirements are incompatible with the CC BY 4.0 license, please either i) remove the figure or ii) supply a replacement figure that complies with the CC BY 4.0 license. Please check copyright information on all replacement figures and update the figure caption with source information. If applicable, please specify in the figure caption text when a figure is similar but not identical to the original image and is therefore for illustrative purposes only.

The following resources for replacing copyrighted map figures may be helpful:

USGS National Map Viewer (public domain): http://viewer.nationalmap.gov/viewer/

The Gateway to Astronaut Photography of Earth (public domain): http://eol.jsc.nasa.gov/sseop/clickmap/

Maps at the CIA (public domain): https://www.cia.gov/library/publications/the-world-factbook/index.html and https://www.cia.gov/library/publications/cia-maps-publications/index.html

NASA Earth Observatory (public domain): http://earthobservatory.nasa.gov/

Landsat: http://landsat.visibleearth.nasa.gov/

USGS EROS (Earth Resources Observatory and Science (EROS) Center) (public domain): http://eros.usgs.gov/#

Natural Earth (public domain): http://www.naturalearthdata.com/

8. We notice that your supplementary table is included in the manuscript file. Please remove them and upload them with the file type 'Supporting Information'. Please ensure that each Supporting Information file has a legend listed in the manuscript after the references list.

9. If the reviewer comments include a recommendation to cite specific previously published works, please review and evaluate these publications to determine whether they are relevant and should be cited. There is no requirement to cite these works unless the editor has indicated otherwise.

Additional Editor Comments:

Thank you for submitting “The consumption of dietary supplements among active individuals in Saudi Arabia” to PLOS ONE. We appreciate the time, effort, and thought you have invested in this important work. Both reviewers recognize that your topic is timely and highly relevant for public health in Saudi Arabia. At the same time, they have identified several key areas—ranging from methods to analysis and presentation—where the manuscript could be strengthened. With thoughtful revisions, your study has the potential to make a meaningful contribution to the field.

First, please update your literature review and discussion to include recent, region-specific studies. Reviewer 1 has pointed to three particularly relevant papers—Aljebeli et al. (Cureus, 2023), Alotaibi et al. (Saudi Pharmaceutical Journal, 2024), and Alshehri et al. (Nutrients, 2025)—that will help sharpen your rationale and place your findings in the context of current Saudi data, including among female fitness-center populations. Integrating these references will also strengthen your interpretation of knowledge and attitude results. Where your findings differ from recent reports, briefly explore possible reasons, such as differences in sampling, setting, or definitions.

Second, please clarify your sampling approach and discuss its limitations more transparently. While you describe a two-stage cluster design with region-specific targets, actual recruitment was carried out online with the help of volunteers and gym administrators. This may have led to selection bias, favoring gym-affiliated and digitally connected participants, and under-representing others like community sports club members or informal exercisers. To strengthen your methods section, please: (a) explain how you compiled gym lists, which platforms you used, and how many gyms were contacted versus those that participated; (b) report the denominator, response rate, and regional yield; and (c) discuss representativeness in light of the large number of fitness facilities in Saudi Arabia, as Reviewer 1 suggested. If you did not use sampling weights, please update your description to reflect a nonprobability sample and temper your conclusions accordingly. These sampling considerations, along with recall and social desirability bias from self-reporting, should be clearly acknowledged in your Limitations section.

Third, please clarify your definitions and measurements. “Dietary supplements” can mean different things in various studies and policies, and products such as energy drinks, herbal remedies, protein powders, and prescribed supplements are often grouped together. Please provide a clear, authoritative definition (such as from the SFDA or DSHEA), specify exactly what you told participants qualified as a supplement, and break down supplement use by category (vitamins/minerals, protein powders, creatine or other ergogenics, herbal products, energy drinks, physician-prescribed products, and those recommended by dietitians). As Reviewer 2 observed, distinguishing between prescribed/nutritionist-suggested supplements and sports-nutrition products will help readers understand the behaviors you’re reporting.

Fourth, consider streamlining and refocusing your tables for clarity. As Reviewer 2 noted, Tables 1 and 2 repeat information that is later stratified in Tables 3 and 4. You can enhance readability by combining baseline characteristics into a single, compact table and moving extra descriptive details to the Supplement. Main-text tables should directly support your study’s objectives—such as showing prevalence overall and by subgroups, or presenting adjusted associations. For Table 5, which currently mixes “knowledge and practice” items, please either (a) explain why these items are included and how they relate to your aims or (b) move less relevant items to the Supplement and revise the text to align outcomes with objectives. Across all tables, standardize denominators, ensure percentages add up, report exact p-values, and avoid overlapping bivariate tests that could inflate error rates. Focusing your tables this way will make your findings easier to interpret and more impactful.

Fifth, please revise your modeling strategy and reporting. Currently, there are inconsistencies in the structural equation modeling (SEM) results—for example, reporting a “non-significant Chi-square” together with a “P-value: 0.000.” Please re-calculate and report fit indices (including χ² with degrees of freedom and p-value, CFI/TLI, RMSEA with 90% CI, and SRMR), using an estimator suited for ordinal data, such as WLSMV. Before conducting path analysis, establish your measurement models for the latent constructs (“knowledge and practices” and “health”) by including factor loadings, reliability (ω/α), and rationale for item inclusion. Please also explain why age and nationality were removed for “low loadings,” since these are demographic variables, not latent factor indicators. If you continue using SEM, clarify what specific research question it addresses, and avoid causal language since your data are cross-sectional. Addressing these modeling issues will help readers trust and understand your analytic approach.

Sixth, please make sure your aims, results, and conclusions are all aligned. Reviewer 2 pointed out that your conclusion suggests supplements had effects on endurance, energy, or health—outcomes not directly assessed in your study. If you wish to report participants’ perceptions of such effects, add this explicitly to your Objectives, specify which survey items measured perceived effects, and present those results consistently throughout your manuscript. Otherwise, revise your Abstract, Discussion, and Conclusion to keep the focus on prevalence, patterns, and correlates of supplement use, rather than on any effects.

Seventh, please strengthen your documentation of the survey instrument and data transparency. While you mention that the questionnaire was “self-developed” and “adapted from validated questionnaires,” it’s important to detail how you adapted and translated the instrument, including any pilot testing or psychometric evaluation. Please provide the original sources, describe the adaptation and translation/back-translation steps, and summarize cognitive testing and reliability metrics for each scale. Consider including item wording in a Supplement. Also, per PLOS ONE requirements, make the de-identified dataset and code available in a stable repository with DOIs at the time of revision, and update your Data Availability Statement accordingly. Finally, please confirm that your funding statement makes clear the funder’s role (or lack thereof) in study design, analysis, and publication decisions, and that your ethics and consent reporting matches both the Methods and submission system.

Eighth, your manuscript would benefit from careful language and presentation polishing. There are some recurring grammatical errors (e.g., “predectors,” “demonistrated”), spacing and capitalization inconsistencies, rounding issues, and a few contradictions between text and figures. We recommend a professional English-language edit and a final technical review to ensure consistency in your data and presentation. These steps will help your work read smoothly and appear as strong as possible.

In your rebuttal, in addition to responding to both reviewers’ comments point by point, please also include: (1) a clear plan for which tables will stay in the main text versus the Supplement (and your reasoning); (2) a precise operational definition of “dietary supplement” as used in your survey, along with updated, category-specific results; (3) corrected and fully reported SEM/CFA results (or your rationale for removing these analyses, if you choose to focus on descriptive or associational findings); (4) an expanded Limitations section, including discussion of selection and coverage bias from your online, gym-based recruitment and whether sampling weights were used; and (5) updated references from 2023–2025, including but not limited to the three key works suggested by Reviewer 1.

We look forward to receiving your thoroughly revised manuscript, along with a detailed response to reviewers and tracked-changes files. This decision is based on the version you submitted and the peer reviews provided. Thank you again for your hard work and commitment to improving your study.

[Note: HTML markup is below. Please do not edit.]

Reviewers' comments:

Reviewer's Responses to Questions

**Comments to the Author**

1. Is the manuscript technically sound, and do the data support the conclusions?

The manuscript must describe a technically sound piece of scientific research with data that supports the conclusions. Experiments must have been conducted rigorously, with appropriate controls, replication, and sample sizes. The conclusions must be drawn appropriately based on the data presented.

Reviewer #1: Yes

Reviewer #2: Partly

**********

2. Has the statistical analysis been performed appropriately and rigorously?

Reviewer #1: Yes

Reviewer #2: Yes

**********

3. Have the authors made all data underlying the findings in their manuscript fully available?

The PLOS Data policy requires authors to make all data underlying the findings described in their manuscript fully available without restriction, with rare exception (please refer to the Data Availability Statement in the manuscript PDF file). The data should be provided as part of the manuscript or its supporting information, or deposited to a public repository. For example, in addition to summary statistics, the data points behind means, medians and variance measures should be available. If there are restrictions on publicly sharing data—e.g. participant privacy or use of data from a third party—those must be specified.

Reviewer #1: Yes

Reviewer #2: No

**********

4. Is the manuscript presented in an intelligible fashion and written in standard English?

PLOS ONE does not copyedit accepted manuscripts, so the language in submitted articles must be clear, correct, and unambiguous. Any typographical or grammatical errors should be corrected at revision, so please note any specific errors here.

Reviewer #1: Yes

Reviewer #2: Yes

**********

5. Review Comments to the Author

Please use the space provided to explain your answers to the questions above. You may also include additional comments for the author, including concerns about dual publication, research ethics, or publication ethics. (Please upload your review as an attachment if it exceeds 20,000 characters)

Reviewer #1: This manuscript investigates the prevalence and patterns of dietary supplement consumption among physically active individuals in Saudi Arabia. This is an interesting topic given the increasing popularity of supplements and limited data in this region.

I have few recommendations

more updated references should be cited

1) Aljebeli, Shahad et al. “The Prevalence and Awareness of Dietary Supplement Use Among Saudi Women Visiting Fitness Centers in Riyadh, Saudi Arabia.” Cureus vol. 15,6 e41031. 27 Jun. 2023, doi:10.7759/cureus.41031

2) Alotaibi, Abdulkarim F et al. “Pattern of dietary supplement use and its psychosocial predictors among females visiting fitness centers in Saudi Arabia: Findings from a cross-sectional study.” Saudi pharmaceutical journal : SPJ : the official publication of the Saudi Pharmaceutical Society vol. 32,3 (2024): 101966. doi:10.1016/j.jsps.2024.101966

Alshehri, A.A.; Alqahtani, S.; Aldajani, R.; Alsharabi, B.; Alzahrani, W.; Alguthami, G.; Khawagi, W.Y.; Arida, H. Knowledge, Attitudes, and Practices of Dietary Supplement Use in Western Saudi Arabia: A Cross-Sectional Study. Nutrients 2025, 17, 1233. https://doi.org/10.3390/nu17071233

I think more limitation should be addressed such as

selection bias as the questionnaire was distributed online though gym administration taking into account that there is around 3,966 fitness gym is KSA. Participants may be recruited from gyms, fitness centers, or sports clubs, which may not represent all active Saudis.

Participants may include herbal products, energy drinks, protein powders, or even medications under "supplements," which makes comparisons difficult and inn the study there was clarification about supplements used by the participants

Reviewer #2: Tables 1 & 2 are additional, while these characteristics are already presented in Tables 3 & 4 with more details in relation to the use or non-use of nutritional supplements. There is information about the specific use or consumption of nutritional supplements, including vitamins, herbs etc. and further it can be categorized with other health characteristics, etc.

The details of prescribed supplements in addition to dietary nutritional supplements and the nutritionist's suggested supplements are not categorized.

Data presented in table 5 related to the knowledge and practice in relation to consumption of nutritional supplements is irrelevant and not in line with the rationale of the study.

Conclusion of study is also not in accordance with the findings/results, as the effect of nutritional supplements is not the objective of study. the perception of participants about their effect on their health should be added in the objectives and detailed in the results and discussion part.

**********

6. PLOS authors have the option to publish the peer review history of their article (what does this mean?). If published, this will include your full peer review and any attached files.

If you choose “no”, your identity will remain anonymous but your review may still be made public.

**Do you want your identity to be public for this peer review?**   For information about this choice, including consent withdrawal, please see our Privacy Policy.

Reviewer #1: **Yes:**  Khloud Ghafouri

Reviewer #2: **Yes:**  Intisar Ahmad Siddiqui

**********

[NOTE: If reviewer comments were submitted as an attachment file, they will be attached to this email and accessible via the submission site. Please log into your account, locate the manuscript record, and check for the action link "View Attachments". If this link does not appear, there are no attachment files.]

While revising your submission, please upload your figure files to the Preflight Analysis and Conversion Engine (PACE) digital diagnostic tool, https://pacev2.apexcovantage.com/. PACE helps ensure that figures meet PLOS requirements. To use PACE, you must first register as a user. Registration is free. Then, login and navigate to the UPLOAD tab, where you will find detailed instructions on how to use the tool. If you encounter any issues or have any questions when using PACE, please email PLOS at figures@plos.org. Please note that Supporting Information files do not need this step.

# Author comment: Author response to Decision Letter 1

**DOI:** 10.1371/journal.pone.0351208.r002

3 Nov 2025

Response to Reviewers - PLOS ONE

The consumption of dietary supplements among active individuals in Saudi Arabia

Manuscript ID: PONE-D-25-22005

Dear Academic Editor,

Jordan Llego, PhD ELM, D. Hon. Ex., PhDN, RN.

Thank you for the opportunity to revise our manuscript and for the insightful comments provided by you and the reviewers. We appreciate the time and effort invested in evaluating our work. We have carefully considered all feedback and revised the manuscript accordingly. Below, we provided a detailed point-by-point response to each comment.

#Journal Requirements and Author Response

Comment 1: Please ensure that your manuscript meets PLOS ONE's style requirements, including those for file naming. The PLOS ONE style templates can be found at

https://journals.plos.org/plosone/s/file?id=wjVg/PLOSOne_formatting_sample_main_body.pdf and

https://journals.plos.org/plosone/s/file?id=ba62/PLOSOne_formatting_sample_title_authors_affiliations.pdf

Author Response: Thank you for the links provided. We followed the instructions carefully for the development of revised manuscript and also labelled the files for submission as:

‘Response to Reviewers’

‘Revised Manuscript with Track Changes’

‘Manuscript’

Comment 2: Please provide additional details regarding participant consent. In the ethics statement in the Methods and online submission information, please ensure that you have specified (1) whether consent was informed and (2) what type you obtained (for instance, written or verbal, and if verbal, how it was documented and witnessed). If your study included minors, state whether you obtained consent from parents or guardians. If the need for consent was waived by the ethics committee, please include this information.

If you are reporting a retrospective study of medical records or archived samples, please ensure that you have discussed whether all data were fully anonymized before you accessed them and/or whether the IRB or ethics committee waived the requirement for informed consent. If patients provided informed written consent to have data from their medical records used in research, please include this information.

Author Response: Data collected through online questionnaire. We agree with the editor observation, and have added details regarding participant consent.

Ethical consideration

Ethical issues were addressed in accordance with the guidelines of the Declaration of Helsinki and the Saudi Bioethics standards. Participants were informed through a structure narrative at the beginning of the Google Form questionnaire about the study purpose, their right to withdraw at any time with no penalty, and assurance regarding the confidentiality of their response. Digitally recorded the written informed consent before participate in the survey. Ethical clearance for this study was granted by the Standing Committee for Scientific Research of Princess Nourah Bint Abdulrahman University (PNURSP2025R207), Riyadh, Saudi Arabia.

[Page 6, line 168-183].

Comment 3: Thank you for stating the following financial disclosure:

“Princess Nourah Bint Abdulrahman University Researchers Supporting Project number (PNURSP2025R207).”

Please state what role the funders took in the study. If the funders had no role, please state: 'The funders had no role in study design, data collection and analysis, decision to publish, or preparation of the manuscript.'

If this statement is not correct you must amend it as needed.

Please include this amended Role of Funder statement in your cover letter; we will change the online submission form on your behalf.

Author Response: Thanks to the editor for this suggestion. We have added the statement that “Princess Nourah Bint Abdulrahman University Researchers Supporting Project number (PNURSP2025R207), Riyadh, Saudi Arabia. The funders had no role in study design, data collection and analysis, decision to publish, or preparation of the manuscript.” and also mentioned the same in the cover letter [Page 18, line 464-467].

Comment 4: Thank you for stating the following in the Acknowledgments Section of your manuscript: “the authors would like to acknowledge and appreciate all participants for their cooperation in filling out the survey to complete this study, in addition to deep grateful for Princess Nourah bint Abdulrahman University for funding and supporting this work.”

We note that you have provided funding information that is currently declared in your Funding Statement. However, funding information should not appear in the Acknowledgments section or other areas of your manuscript. We will only publish funding information present in the Funding Statement section of the online submission form.

Please remove any funding-related text from the manuscript and let us know how you would like to update your Funding Statement. Currently, your Funding Statement reads as follows:

“Princess Nourah Bint Abdulrahman University Researchers Supporting Project number (PNURSP2025R207).”

Please include your amended statements within your cover letter; we will change the online submission form on your behalf.

Author Response: Thanks to the editor for this observation. We have removed funding-related text from acknowledgement section and updated the narrative as “The authors would like to acknowledge and appreciate all participants for their cooperation in filling out the survey to complete this study” [Page 18, line 470-471]

Updated funding statement: “Princess Nourah Bint Abdulrahman University Researchers Supporting Project (PNURSP2025R207), Riyad, Saudi Arabia.” [Page 18, line 464-467].

We have included the amended funding statements in the cover letter

Comment 5: We note that your Data Availability Statement is currently as follows: All relevant data are within the manuscript and its Supporting Information files.

Please confirm at this time whether or not your submission contains all raw data required to replicate the results of your study. Authors must share the “minimal data set” for their submission. PLOS defines the minimal data set to consist of the data required to replicate all study findings reported in the article, as well as related metadata and methods

(https://journals.plos.org/plosone/s/data-availability#loc-minimal-data-set-definition).

For example, authors should submit the following data:

- The values behind the means, standard deviations and other measures reported;

- The values used to build graphs;

- The points extracted from images for analysis.

Authors do not need to submit their entire data set if only a portion of the data was used in the reported study.

If your submission does not contain these data, please either upload them as Supporting Information files or deposit them to a stable, public repository and provide us with the relevant URLs, DOIs, or accession numbers. For a list of recommended repositories, please see https://journals.plos.org/plosone/s/recommended-repositories.

If there are ethical or legal restrictions on sharing a de-identified data set, please explain them in detail (e.g., data contain potentially sensitive information, data are owned by a third-party organization, etc.) and who has imposed them (e.g., an ethics committee). Please also provide contact information for a data access committee, ethics committee, or other institutional body to which data requests may be sent. If data are owned by a third party, please indicate how others may request data access.

Author Response: We agree with the editor observation. Now provided the raw data Excel file [V1 (29092025 - V8_NS).xlsx] to replicate and assess the accuracy of the results by publishing authority. Requesting the editor to use sheet-4 of the file.

Comment 6: Please include a separate caption for each figure in your manuscript.

Author Response: Thank you for this observation. We have removed Figure 1 from the manuscripts and adjusted the other figure numbers. Written separate caption for each figure in the manuscript.

Remove “Fig 1” and include “Table S1” [Page 5, Line 147].

“Fig 2A” changed to “Fig 1A” [Page 11, Line 250].

“Fig 2B” changed to “Fig 1B” [Page 12, Line 252].

“Fig 2C” changed to “Fig 1C” [Page 12, Line 253].

Caption: “Figure 1(A-C) Active individuals buying place, reasons for use and source of information on nutrition supplements” [Page 23, line 608-609].

“Fig 3” changed to “Fig 2” [Page 12, Line 256].

Caption: “Figure 2 Participants’ perceptions on the use of nutrition supplements” [Page 23, line 611].

“Fig 4” changed to “Fig 3” [Page 12, Line 271].

Caption: “Figure 3: Factors associated with the consumption of nutrition supplements”

Legend: “Figure 3 Demographic and background characteristics (DEMO): GEN-Gender, MS-Marital status, REG-Region, EL-Education level, ES-Employment status, MI-Monthly income per capita (in Saudi riyals "SR "), SMO-Smoking. Health characteristics (HEA): GH-General health, EWC-Weight control program with a dietitian, GFL-General fitness level, PA-Physical activity per week, PS-Professionalism in sport. Knowledge and practices (KP): UNS-Use nutritional supplements, KNS-Knowledge on nutritional supplement ingredients, UCC- Use supplement contain caffeine, EBNS- Effectiveness and benefit of the nutritional supplement, MSNS-Monthly spent on nutritional supplements - (SR ) and *Highly Significant”

[Page 23, line 613-621]

Comment 7: We note that Figure 1 in your submission contain map/satellite image which may be copyrighted. All PLOS content is published under the Creative Commons Attribution License (CC BY 4.0), which means that the manuscript, images, and Supporting Information files will be freely available online, and any third party is permitted to access, download, copy, distribute, and use these materials in any way, even commercially, with proper attribution. For these reasons, we cannot publish previously copyrighted maps or satellite images created using proprietary data, such as Google software (Google Maps, Street View, and Earth). For more information, see our copyright guidelines: http://journals.plos.org/plosone/s/licenses-and-copyright.

We require you to either (1) present written permission from the copyright holder to publish these figures specifically under the CC BY 4.0 license, or (2) remove the figures from your submission:

a. You may seek permission from the original copyright holder of Figure 1 to publish the content specifically under the CC BY 4.0 license.

We recommend that you contact the original copyright holder with the Content Permission Form (http://journals.plos.org/plosone/s/file?id=7c09/content-permission-form.pdf) and the following text:

“I request permission for the open-access journal PLOS ONE to publish XXX under the Creative Commons Attribution License (CCAL) CC BY 4.0 (http://creativecommons.org/licenses/by/4.0/). Please be aware that this license allows unrestricted use and distribution, even commercially, by third parties. Please reply and provide explicit written permission to publish XXX under a CC BY license and complete the attached form.”

Please upload the completed Content Permission Form or other proof of granted permissions as an 'Other' file with your submission.

In the figure caption of the copyrighted figure, please include the following text: “Reprinted from [ref] under a CC BY license, with permission from [name of publisher], original copyright [original copyright year].”

b. If you are unable to obtain permission from the original copyright holder to publish these figures under the CC BY 4.0 license or if the copyright holder’s requirements are incompatible with the CC BY 4.0 license, please either i) remove the figure or ii) supply a replacement figure that complies with the CC BY 4.0 license. Please check copyright information on all replacement figures and update the figure caption with source information. If applicable, please specify in the figure caption text when a figure is similar but not identical to the original image and is therefore for illustrative purposes only.

The following resources for replacing copyrighted map figures may be helpful:

USGS National Map Viewer (public domain): http://viewer.nationalmap.gov/viewer/

The Gateway to Astronaut Photography of Earth (public domain): http://eol.jsc.nasa.gov/sseop/clickmap/

Maps at the CIA (public domain): https://www.cia.gov/library/publications/the-world-factbook/index.html and https://www.cia.gov/library/publications/cia-maps-publications/index.html

NASA Earth Observatory (public domain): http://earthobservatory.nasa.gov/

Landsat: http://landsat.visibleearth.nasa.gov/

USGS EROS (Earth Resources Observatory and Science (EROS) Center) (public domain): http://eros.usgs.gov/#

Natural Earth (public domain): http://www.naturalearthdata.com/

Author Response: Thank you for this observation. We have written R-program and created the map of Saudi Arabia with the longitudes and latitudes of regions from https://mapcruzin.com/free-saudi-arabia-country-city-place-gis-shapefiles.htm and is licensed under Creative Commons Attribution Share-Alike 2.0 (CC BY-SA 2.0). Moreover, website is saying that to use without any permission. Not copied elsewhere therefore, we are unable to get permission from the copy right holder and decided to remove the figure 1 from the manuscript.

Comment 8: We notice that your supplementary table is included in the manuscript file. Please remove them and upload them with the file type 'Supporting Information'. Please ensure that each Supporting Information file has a legend listed in the manuscript after the references list.

Author Response: We agree with the editor observation. Removed the supplementary table from the manuscript and written the caption and legend of the supporting file after the references.

Caption: “Table S1. Sampling frame and the active individuals” [Page 23, line 625-632]

Legend: “Sample size (n) calculated by the Single proportion (Z2*P*(1-P))/e2. Where Z: Value from standard normal Distribution corresponding to desired confidence level (Z=1.96 for 95% CI), P: Expected true proportione: Desired precision, NR:Non Response Rate, Proportion: Percentage of individuals aged 18 years or above who practice sports activity (150 minutes and more per week) at the dministrative region level (Source:Household Sports Practice Survey 2019, General Authority for Statistics)” [Page 23, line 625-632]

Comment 9: If the reviewer comments include a recommendation to cite specific previously published works, please review and evaluate these publications to determine whether they are relevant and should be cited. There is no requirement to cite these works unless the editor has indicated otherwise.

Author Response: Thanks to the editor for this suggestion. Reviewer-1 suggested three recent, Saudi Arabia country specific studies. Aljebeli et al. (Cureus, 2023), Alotaibi et al. (Saudi Pharmaceutical Journal, 2024), and Alshehri et al. (Nutrients, 2025). We have reviewed and agreed to reviewer 1 suggestion to include and update the literature review and discussion by include in the manuscript. [Page 2, line 62-68 & Page 13, line 290-291]

Additional Editor Comments:

Thank you for submitting “The consumption of dietary supplements among active individuals in Saudi Arabia” to PLOS ONE. We appreciate the time, effort, and thought you have invested in this important work. Both reviewers recognize that your topic is timely and highly relevant for public health in Saudi Arabia. At the same time, they have identified several key areas—ranging from methods to analysis and presentation—where the manuscript could be strengthened. With thoughtful revisions, your study has the potential to make a meaningful contribution to the field.

Comment: First, please update your literature review and discussion to include recent, region-specific studies. Reviewer 1 has pointed to three particularly relevant papers—Aljebeli et al. (Cureus, 2023), Alotaibi et al. (Saudi Pharmaceutical Journal, 2024), and Alshehri et al. (Nutrients, 2025)—that will help sharpen your rationale and place your findings in the context of current Saudi data, including among female fitness-center populations. Integrating these references will also strengthen your interpretation of knowledge and attitude results. Where your findings

Attachment Submitted filename: Response to Reviewers 2.docx

# Peer review: Decision Letter 1

**DOI:** 10.1371/journal.pone.0351208.r003

**Authors:** Jordan Llego

18 Nov 2025

PONE-D-25-22005R1

The consumption of dietary supplements among active individuals in Saudi Arabia

PLOS ONE

Dear Dr. Alsayegh,

Thank you for submitting your manuscript to PLOS ONE. After careful consideration, we feel that it has merit but does not fully meet PLOS ONE’s publication criteria as it currently stands. Therefore, we invite you to submit a revised version of the manuscript that addresses the points raised during the review process.

Please submit your revised manuscript by Jan 02 2026 11:59PM. If you will need more time than this to complete your revisions, please reply to this message or contact the journal office at plosone@plos.org. When you're ready to submit your revision, log on to https://www.editorialmanager.com/pone/ and select the 'Submissions Needing Revision' folder to locate your manuscript file.

Please include the following items when submitting your revised manuscript:

- A rebuttal letter that responds to each point raised by the academic editor and reviewer(s). You should upload this letter as a separate file labeled 'Response to Reviewers'.
- A marked-up copy of your manuscript that highlights changes made to the original version. You should upload this as a separate file labeled 'Revised Manuscript with Track Changes'.
- An unmarked version of your revised paper without tracked changes. You should upload this as a separate file labeled 'Manuscript'.

If you would like to make changes to your financial disclosure, please include your updated statement in your cover letter. Guidelines for resubmitting your figure files are available below the reviewer comments at the end of this letter.

If applicable, we recommend that you deposit your laboratory protocols in protocols.io to enhance the reproducibility of your results. Protocols.io assigns your protocol its own identifier (DOI) so that it can be cited independently in the future. For instructions see: https://journals.plos.org/plosone/s/submission-guidelines#loc-laboratory-protocols. Additionally, PLOS ONE offers an option for publishing peer-reviewed Lab Protocol articles, which describe protocols hosted on protocols.io. Read more information on sharing protocols at https://plos.org/protocols?utm_medium=editorial-email&utm_source=authorletters&utm_campaign=protocols.

We look forward to receiving your revised manuscript.

Kind regards,

Jordan Llego, PhD ELM, D. Hon. Ex., PhDN, RN

Academic Editor

PLOS ONE

Journal Requirements:

If the reviewer comments include a recommendation to cite specific previously published works, please review and evaluate these publications to determine whether they are relevant and should be cited. There is no requirement to cite these works unless the editor has indicated otherwise.

Additional Editor Comments:

Thank you for submitting the revised version of your manuscript, “The consumption of dietary supplements among active individuals in Saudi Arabia.” We appreciate all the effort you’ve put into responding to the initial feedback from reviewers and the editorial office. Your revisions show real commitment, especially with the updated literature review, moving some tables to the supplementary section, removing copyrighted materials, and expanding the limitations. Despite these improvements, a careful review shows that the manuscript still isn’t quite ready for publication. A number of important methodological, analytical, and reporting issues still need attention before the paper can progress.

One major concern is the structural equation modeling. Although you recalculated the model using the recommended WLSMV estimator, the fit indices are still below the journal’s standards, and the reporting needs to be more transparent. Key details—such as confirmatory factor analysis results, factor loadings, measurement model validation, and reliability for latent constructs—are missing. The reasoning behind grouping different items into latent variables also needs more explanation. Additionally, age and nationality are still presented as if they’re psychometric constructs rather than categorical predictors. These points affect the statistical validity of the model and should be clarified or reconsidered.

The sampling strategy also needs clearer explanation. While you’ve clarified some parts of the process, the manuscript still doesn’t mention how many gyms were contacted, the response rate, or exactly how volunteers and administrators were involved in recruiting participants. It’s also not clear how representative the sample is, since the study uses non-probability purposive sampling among gym-goers, but the conclusions suggest national-level generalizability. Please consider either adjusting the claims or providing more detail to make the methodology transparent.

The data availability statement still doesn’t meet PLOS ONE requirements. The journal asks that the minimal dataset be publicly available unless there’s a strong ethical or legal reason not to. Saying that data is available upon request isn’t enough. Please update this section either by depositing the dataset in a public repository or by giving a clear justification, supported by your institution’s policy, and naming a data access committee if needed.

The way dietary supplements are defined and categorized still needs work. You’ve included the DSHEA definition, but the results don’t separate out the different types of supplements participants used. Because “dietary supplements” covers such a wide range, not breaking them down makes it hard to interpret the findings. Reviewers specifically asked for a clear distinction between products like vitamins, herbs, protein powders, energy drinks, and prescribed supplements. This breakdown should be included in both the methods and results sections.

The questionnaire documentation also needs more detail. While you mention a pilot study and approval from the National Nutrition Committee, the manuscript still doesn’t explain how the questionnaire was adapted and translated, how cognitive testing was done, or provide psychometric details like internal consistency or construct validity. These are important for transparency and reproducibility, especially since the instrument is self-developed and key to your study. Please also include the full questionnaire or its item list as supplementary material.

There are also some conceptual and interpretive inconsistencies to address. The abstract, discussion, and conclusion still imply that dietary supplements affect health and performance, even though the study didn’t measure these outcomes. If perceived effects are part of your objectives, please make this clear in your analysis and presentation. If not, revise the conclusions so they match the descriptive and correlational nature of your data, and avoid using causal language throughout the manuscript.

Finally, even though you submitted a proofreading certificate, there are still some grammar issues, formatting inconsistencies, repeated statements, and unclear phrases that affect readability. Tables, figures, and statistical outputs would also benefit from careful editing to improve their accuracy and clarity.

Because of these remaining concerns, the manuscript will need another significant revision before it can be considered for publication. We encourage you to address all the methodological, statistical, and reporting issues, make sure your conclusions match your data, provide more transparency about the survey instrument, and review the language and presentation throughout. With thoughtful and thorough revision, this study could offer valuable insights into dietary supplement use among active individuals in Saudi Arabia. We look forward to seeing your revised version.

[Note: HTML markup is below. Please do not edit.]

Reviewers' comments:

Reviewer's Responses to Questions

**Comments to the Author**

1. If the authors have adequately addressed your comments raised in a previous round of review and you feel that this manuscript is now acceptable for publication, you may indicate that here to bypass the “Comments to the Author” section, enter your conflict of interest statement in the “Confidential to Editor” section, and submit your "Accept" recommendation.

Reviewer #2: All comments have been addressed

**********

2. Is the manuscript technically sound, and do the data support the conclusions?

The manuscript must describe a technically sound piece of scientific research with data that supports the conclusions. Experiments must have been conducted rigorously, with appropriate controls, replication, and sample sizes. The conclusions must be drawn appropriately based on the data presented.

Reviewer #2: Yes

**********

3. Has the statistical analysis been performed appropriately and rigorously?

Reviewer #2: Yes

**********

4. Have the authors made all data underlying the findings in their manuscript fully available?

The PLOS Data policy requires authors to make all data underlying the findings described in their manuscript fully available without restriction, with rare exception (please refer to the Data Availability Statement in the manuscript PDF file). The data should be provided as part of the manuscript or its supporting information, or deposited to a public repository. For example, in addition to summary statistics, the data points behind means, medians and variance measures should be available. If there are restrictions on publicly sharing data—e.g. participant privacy or use of data from a third party—those must be specified.

Reviewer #2: Yes

**********

5. Is the manuscript presented in an intelligible fashion and written in standard English?

PLOS ONE does not copyedit accepted manuscripts, so the language in submitted articles must be clear, correct, and unambiguous. Any typographical or grammatical errors should be corrected at revision, so please note any specific errors here.

Reviewer #2: Yes

**********

6. Review Comments to the Author

Please use the space provided to explain your answers to the questions above. You may also include additional comments for the author, including concerns about dual publication, research ethics, or publication ethics. (Please upload your review as an attachment if it exceeds 20,000 characters)

Reviewer #2: The revision has addressed the comments, and modifications have been made in the revised manuscript. It can be accepted for further processing.

**********

7. PLOS authors have the option to publish the peer review history of their article (what does this mean?). If published, this will include your full peer review and any attached files.

If you choose “no”, your identity will remain anonymous but your review may still be made public.

**Do you want your identity to be public for this peer review?**   For information about this choice, including consent withdrawal, please see our Privacy Policy.

Reviewer #2: **Yes:**  Intisar Ahmad Siddiqui

**********

[NOTE: If reviewer comments were submitted as an attachment file, they will be attached to this email and accessible via the submission site. Please log into your account, locate the manuscript record, and check for the action link "View Attachments". If this link does not appear, there are no attachment files.]

To ensure your figures meet our technical requirements, please review our figure guidelines: https://journals.plos.org/plosone/s/figures

You may also use PLOS’s free figure tool, NAAS, to help you prepare publication quality figures: https://journals.plos.org/plosone/s/figures#loc-tools-for-figure-preparation.

NAAS will assess whether your figures meet our technical requirements by comparing each figure against our figure specifications.

# Author comment: Author response to Decision Letter 2

**DOI:** 10.1371/journal.pone.0351208.r004

11 Jan 2026

Response to Additional Editor Comments - PLOS ONE

The consumption of dietary supplements among active individuals in Saudi Arabia

Manuscript ID: PONE-D-25-22005

Dear Academic Editor,

Jordan Llego, PhD ELM, D. Hon. Ex., PhDN, RN.

Thank you for the opportunity to revise our manuscript and for the insightful comments provided by you. We appreciate the time and effort invested in evaluating our work. We have carefully considered all feedback and revised the manuscript accordingly. Below, we provided a detailed point-by-point response to each comment.

Comment 1: One major concern is the structural equation modeling. Although you recalculated the model using the recommended WLSMV estimator, the fit indices are still below the journal’s standards, and the reporting needs to be more transparent. Key details—such as confirmatory factor analysis results, factor loadings, measurement model validation, and reliability for latent constructs—are missing. The reasoning behind grouping different items into latent variables also needs more explanation. Additionally, age and nationality are still presented as if they’re psychometric constructs rather than categorical predictors. These points affect the statistical validity of the model and should be clarified or reconsidered.

Author Response: Thank you for this valuable observation. We followed point by point carefully to respond. Bivariate analysis using chi-square (Table 1) identified that age and nationality variables are non-significant predictors (p > 0.05) for the consumption of nutritional supplements. Measurement validation through Confirmatory Factor Analysis (CFA), where items Region (REG) and use supplements contain caffeine (UCC) were not met the factor loadings threshold 0.20 (Figure S2). Justifying the exclusion of four variables from subsequent modelling to reduce noise, the structural equation modelling (SEM) plot indicating 𝑥2 = 2047.530 (d.f = 99, p = 0.00), comparative fit index = 0.901, root mean square error of approximation = 0.072 (90% CI: 0.069–0.075), and standardized root mean squared residual = 0.098. The obtained three factors’ domains health (HEA= 0.11), and knowledge and practices (KP = 0.71) had a highly significant influence, while of demographic domain (DEMO = −0.02) not shown a significant relation on the usage of nutrition supplements among the active individuals, and there was no evidence of collinearity in the model (Figure 3). [Manuscript Line 272-283]

Figure 3 annotate have been changed to “Demographic and background characteristics (DEMO): GEN-Gender, MS-Marital status, EL-Education level, ES-Employment status, MI-Monthly income per capita (in Saudi riyals "SR "), SMO-Smoking. Health characteristics (HEA): GH-General health, EWC-Weight control program with a dietitian, GFL-General fitness level, PA-Physical activity per week, PS-Professionalism in sport. Knowledge and practices (KP): UNS-Use nutritional supplements, KNS-Knowledge on nutritional supplement ingredients, EBNS- Effectiveness and benefit of the nutritional supplement, MSNS-Monthly spent on nutritional supplements - (SR ) and *Highly Significant” [Manuscript Line 572-579]

Added supplementary figure and annotate [Manuscript Line 595]

Figure S2. Confirmatory factor analysis

Demographic and background characteristics (DEMO): GEN-Gender, MS-Marital status, REG-Region, EL-Education level, ES-Employment status, MI-Monthly income per capita (in Saudi riyals "SR "), SMO-Smoking. Health characteristics (HEA): GH-General health, EWC-Weight control program with a dietitian, GFL-General fitness level, PA-Physical activity per week, PS-Professionalism in sport. Knowledge and practices (KP): UNS-Use nutritional supplements, KNS-Knowledge on nutritional supplement ingredients, UCC-Use supplements contain caffeine, EBNS- Effectiveness and benefit of the nutritional supplement, MSNS-Monthly spent on nutritional supplements - (SR ) and *Highly Significant” CFA plot indicated 2 = 2619.559 (d.f = 116, p = 0.00), comparative fit index = 0.87, root mean square error of approximation = 0.075 (90% CI: 0.073–0.078), and standardized root mean squared residual = 0.100. [Manuscript Line 596-606]

Comment 2: The sampling strategy also needs clearer explanation. While you’ve clarified some parts of the process, the manuscript still doesn’t mention how many gyms were contacted, the response rate, or exactly how volunteers and administrators were involved in recruiting participants. It’s also not clear how representative the sample is, since the study uses non-probability purposive sampling among gym-goers, but the conclusions suggest national-level generalizability. Please consider either adjusting the claims or providing more detail to make the methodology transparent.

Author Response: Thank to the Editor for this valuable observation, we have responded point by point. A total of 90 gym centers were visited across the 13 provinces of Saudi Arabia, assuming an average number of gym users is 50 per each center. Based on this assumption, 4,500 eligible participants were approached. Google Maps or local business listings were used to compile lists of gymnasiums (the study was limited to gym users). Selected gym, eligible participants list with WhatsApp number collected from the gym volunteer/administrators and enrolled the participants using systematic random sampling method to ensure the representativeness of gym users from the entire nation. Every Kth participant (K = Total eligible participants in the selected gym/50) was included systematically from the list until to get the 50 participants from selected gym centers. If any participant not willing then included the following participant for the study. The online questionnaire was distributed through social media WhatsApp with the help of volunteer or gym administrators. In total 3,800 gym users participated with 84% of response rate. [Manuscript Line 159-171]

Comment 3: The data availability statement still doesn’t meet PLOS ONE requirements. The journal asks that the minimal dataset be publicly available unless there’s a strong ethical or legal reason not to. Saying that data is available upon request isn’t enough. Please update this section either by depositing the dataset in a public repository or by giving a clear justification, supported by your institution’s policy, and naming a data access committee if needed.

Author Response: Thanks to the editor for this valuable comment, we have updated the data availability statement. Data cannot be shared publicly because the data contains potentially identifying information. The research committee at the Clinical Nutrition Department, Jazan University has imposed data sharing restrictions on this study. Data requests can be directed to the corresponding author and to the chair of the research committee at the department (Dr. Huda Mubaraki cln.ams@jazanu.edu.sa) for researchers who meet the criteria for access to confidential data.

Comment 4: The way dietary supplements are defined and categorized still needs work. You’ve included the DSHEA definition, but the results don’t separate out the different types of supplements participants used. Because “dietary supplements” covers such a wide range, not breaking them down makes it hard to interpret the findings. Reviewers specifically asked for a clear distinction between products like vitamins, herbs, protein powders, energy drinks, and prescribed supplements. This breakdown should be included in both the methods and results sections.

Author Response: Thanks to the editor for this valuable comment.

In methods After dietary supplements definition: Based on the frequency of consumption of dietary supplements categized into ten main items: Protein (such as amino acids, protein bar, protein powder, creatine, protein milk), vitamins/ minerals (such as multivitamins, vitamin A, vitamin B complex, and vitamin C, calcium, selenium, zinc, and iron), sports drinks, herps, weight gain supplements, High carps bar, Fat lose supplements, omega 3, Collagen and coffee [Manuscript Line 146-151].

In results beginning of the table 3 narration: Among active individuals, supplement use was predominantly concentrated in two categories. Protein (37.00%) and vitamins/minerals (36.11%) were used by more than two-thirds of participants combined, whereas all other supplement types were reported at substantially lower frequencies (Figure S1) [Manuscript Line 243-246]

Added supplementary figure [Manuscript Line 594]

Figure S1. Consumption of dietary supplements by active individuals

Comment 5: The questionnaire documentation also needs more detail. While you mention a pilot study and approval from the National Nutrition Committee, the manuscript still doesn’t explain how the questionnaire was adapted and translated, how cognitive testing was done, or provide psychometric details like internal consistency or construct validity. These are important for transparency and reproducibility, especially since the instrument is self-developed and key to your study. Please also include the full questionnaire or its item list as supplementary material.

Author Response: Thanks to the editor for this valuable observation, we have responded point by point and also uploaded questionnaire as supplementary file.

After a critical review of local and international studies validated tools assessing nutritional supplements use, a questionnaire was adapted with minor modifications to ensure cultural relevance (12,18). Translation into Arabic followed a standardized forward–backward translation process using independent bilingual translators. Face validity was assessed through pilot testing with 25 gym users. Participants were asked to comment on clarity, wording, and understanding of each item. Minor linguistic refinements were made based on participant feedback and the attained Cronbach’s alpha (0.81) entitling the acceptable internal consistency. Content validity was evaluated by the experts from national nutrition committee, Saudi Arabia. Reliability was assed using test-retest method. In which a sample of 25 gym users completed questionnaire twice within two weeks. The outcomes of the two times compared using correlation co-efficient that has shown a significantly stability co-efficient suggesting a good test-retest reliability. A threshold of factor loading greater than 0.2, comparative fit index 0.901 and root mean square error of approximation 0.072 through the confirmatory factor analysis supporting the construct validity of the questionnaire. [Manuscript Line 86-100]

Comment 6: There are also some conceptual and interpretive inconsistencies to address. The abstract, discussion, and conclusion still imply that dietary supplements affect health and performance, even though the study didn’t measure these outcomes. If perceived effects are part of your objectives, please make this clear in your analysis and presentation. If not, revise the conclusions so they match the descriptive and correlational nature of your data, and avoid using causal language throughout the manuscript.

Author Response: Thanks to the editor for this valuable observation and we have rewritten the conclusion. In conclusion, this study revealed that the prevalence of dietary supplement use among active individuals in Saudi Arabia was higher than rates reported internationally and consistent with recent regional studies. Sociodemographic factors, such as gender, employment status, level of income, and education level, showed a significant difference in dietary supplement consumption. Furthermore, health characteristics, such as self-rated health and fitness, were positively associated with dietary supplement consumption. Additionally, the study noted that there was a shift towards social media being the main source of information, which reflects changes in where active individuals obtain health-related information. Further studies are needed to examine the knowledge of active individuals regarding the safety and effectiveness to determine the level of awareness. Hence, it is very important to tailor awareness programs and educate active individuals effectively. [Manuscript Line 408-418]

Attachment Submitted filename: Response to Reviewers.docx

# Editor report: Decision Letter 2

**DOI:** 10.1371/journal.pone.0351208.r005

**Authors:** Jordan Llego

13 Jan 2026

PONE-D-25-22005R2

The consumption of dietary supplements among active individuals in Saudi Arabia

PLOS One

Dear Dr. Alsayegh,

Thank you for submitting your manuscript to PLOS ONE. After careful consideration, we feel that it has merit but does not fully meet PLOS ONE’s publication criteria as it currently stands. Therefore, we invite you to submit a revised version of the manuscript that addresses the points raised during the review process.

Please submit your revised manuscript by Feb 27 2026 11:59PM. If you will need more time than this to complete your revisions, please reply to this message or contact the journal office at plosone@plos.org. When you're ready to submit your revision, log on to https://www.editorialmanager.com/pone/ and select the 'Submissions Needing Revision' folder to locate your manuscript file.

Please include the following items when submitting your revised manuscript:

- A letter that responds to each point raised by the academic editor and reviewer(s). You should upload this letter as a separate file labeled 'Response to Reviewers'.
- A marked-up copy of your manuscript that highlights changes made to the original version. You should upload this as a separate file labeled 'Revised Manuscript with Track Changes'.
- An unmarked version of your revised paper without tracked changes. You should upload this as a separate file labeled 'Manuscript'.

-->If you would like to make changes to your financial disclosure, please include your updated statement in your cover letter. Guidelines for resubmitting your figure files are available below the reviewer comments at the end of this letter.

If applicable, we recommend that you deposit your laboratory protocols in protocols.io to enhance the reproducibility of your results. Protocols.io assigns your protocol its own identifier (DOI) so that it can be cited independently in the future. For instructions see: https://journals.plos.org/plosone/s/submission-guidelines#loc-laboratory-protocols. Additionally, PLOS ONE offers an option for publishing peer-reviewed Lab Protocol articles, which describe protocols hosted on protocols.io. Read more information on sharing protocols at https://plos.org/protocols?utm_medium=editorial-email&utm_source=authorletters&utm_campaign=protocols.

We look forward to receiving your revised manuscript.

Kind regards,

Jordan Llego, PhD ELM, D. Hon. Ex., PhDN, RN

Academic Editor

PLOS One

Journal Requirements:

If the reviewer comments include a recommendation to cite specific previously published works, please review and evaluate these publications to determine whether they are relevant and should be cited. There is no requirement to cite these works unless the editor has indicated otherwise.

Please review your reference list to ensure that it is complete and correct. If you have cited papers that have been retracted, please include the rationale for doing so in the manuscript text, or remove these references and replace them with relevant current references. Any changes to the reference list should be mentioned in the rebuttal letter that accompanies your revised manuscript. If you need to cite a retracted article, indicate the article’s retracted status in the References list and also include a citation and full reference for the retraction notice.

Additional Editor Comments (if provided):

Thank you for sharing the revised version of your manuscript, “The consumption of dietary supplements among active individuals in Saudi Arabia” (Manuscript ID: PONE-D-25-22005R2). We truly appreciate the time and thoughtful effort you put into addressing the earlier editorial and reviewer comments.

After carefully reviewing your revised manuscript and your detailed response to reviewers, I’m pleased to let you know your work has improved substantially and is now very close to meeting the journal’s publication standards. Your study benefits from a large national sample, greater methodological transparency, and clearer reporting of questionnaire development and validation. The discussion is now more balanced and avoids causal overinterpretation. These changes have strengthened both the scientific rigor and clarity of your work.

However, there are still a few important points to address before your manuscript can be accepted.

First, while the structural equation modeling section is now clearer, some concerns remain about the measurement model’s robustness and reporting. Specifically, the relatively low factor-loading threshold, borderline fit indices, and the inclusion of a demographic latent construct with a non-significant path need clearer justification. You might want to strengthen your reporting of the CFA and reliability for each construct, or consider simplifying your analytical approach if that makes sense. Addressing this will help readers have more confidence in your model and its interpretation.

Second, the data availability statement, though expanded, may still need more clarity for full compliance with PLOS ONE policy. Please clearly state the ethical or legal reasons for restricting public access to the dataset and make sure they align with your institution’s regulations. If possible, let us know whether a de-identified or partial dataset could be shared, or clarify exactly under what conditions data access can be granted.

Third, your manuscript would benefit from a careful language edit. While your content is generally clear, there are still a few grammatical errors, typos, and minor inconsistencies in terminology to address for a polished, professional presentation.

In light of these points, the editorial decision is Minor Revision. We encourage you to address the issues above and submit a revised manuscript along with a brief, point-by-point response explaining how you handled each concern.

I believe these final refinements will make your work even stronger and ready for publication in PLOS ONE. We look forward to your revised submission.

[Note: HTML markup is below. Please do not edit.]

[NOTE: If reviewer comments were submitted as an attachment file, they will be attached to this email and accessible via the submission site. Please log into your account, locate the manuscript record, and check for the action link "View Attachments". If this link does not appear, there are no attachment files.]

To ensure your figures meet our technical requirements, please review our figure guidelines: https://journals.plos.org/plosone/s/figures

You may also use PLOS’s free figure tool, NAAS, to help you prepare publication quality figures: https://journals.plos.org/plosone/s/figures#loc-tools-for-figure-preparation.

NAAS will assess whether your figures meet our technical requirements by comparing each figure against our figure specifications.

# Author comment: Author response to Decision Letter 3

**DOI:** 10.1371/journal.pone.0351208.r006

11 Feb 2026

Dear PLOS ONE Editors,

Thank you for the opportunity to revise and resubmit my manuscript to your esteemed journal. I have carefully addressed the editorial comments and updated the submission accordingly. The following revisions have been completed:

1- Responded to all editor comments.

2- Updated the manuscript throughout as required.

3- Added Figure S3.

4- Revised the Data Availability Statement.

5- Included the certificate of professional proofreading.

6- Submitted a tracked-changes version of the manuscript.

7- Updated the NS_CLN_Data_Supporting_File.

I sincerely appreciate your time and consideration, and I look forward to your valuable feedback.

Attachment Submitted filename: Response_to_Reviewers_auresp_3.docx

# Editor report: Decision Letter 3

**DOI:** 10.1371/journal.pone.0351208.r007

**Authors:** Jordan Llego

19 Feb 2026

PONE-D-25-22005R3

Dietary supplement consumption among active individuals in Saudi Arabia

PLOS One

Dear Dr. Alsayegh,

Thank you for submitting your manuscript to PLOS ONE. After careful consideration, we feel that it has merit but does not fully meet PLOS ONE’s publication criteria as it currently stands. Therefore, we invite you to submit a revised version of the manuscript that addresses the points raised during the review process.

Please submit your revised manuscript by Apr 05 2026 11:59PM. If you will need more time than this to complete your revisions, please reply to this message or contact the journal office at plosone@plos.org. When you're ready to submit your revision, log on to https://www.editorialmanager.com/pone/ and select the 'Submissions Needing Revision' folder to locate your manuscript file.

Please include the following items when submitting your revised manuscript:

- A letter that responds to each point raised by the academic editor and reviewer(s). You should upload this letter as a separate file labeled 'Response to Reviewers'.
- A marked-up copy of your manuscript that highlights changes made to the original version. You should upload this as a separate file labeled 'Revised Manuscript with Track Changes'.
- An unmarked version of your revised paper without tracked changes. You should upload this as a separate file labeled 'Manuscript'.

-->If you would like to make changes to your financial disclosure, please include your updated statement in your cover letter. Guidelines for resubmitting your figure files are available below the reviewer comments at the end of this letter.

If applicable, we recommend that you deposit your laboratory protocols in protocols.io to enhance the reproducibility of your results. Protocols.io assigns your protocol its own identifier (DOI) so that it can be cited independently in the future. For instructions see: https://journals.plos.org/plosone/s/submission-guidelines#loc-laboratory-protocols. Additionally, PLOS ONE offers an option for publishing peer-reviewed Lab Protocol articles, which describe protocols hosted on protocols.io. Read more information on sharing protocols at https://plos.org/protocols?utm_medium=editorial-email&utm_source=authorletters&utm_campaign=protocols.

We look forward to receiving your revised manuscript.

Kind regards,

Jordan Llego, PhD ELM, D. Hon. Ex., PhDN, RN

Academic Editor

PLOS One

Journal Requirements:

If the reviewer comments include a recommendation to cite specific previously published works, please review and evaluate these publications to determine whether they are relevant and should be cited. There is no requirement to cite these works unless the editor has indicated otherwise.

Please review your reference list to ensure that it is complete and correct. If you have cited papers that have been retracted, please include the rationale for doing so in the manuscript text, or remove these references and replace them with relevant current references. Any changes to the reference list should be mentioned in the rebuttal letter that accompanies your revised manuscript. If you need to cite a retracted article, indicate the article’s retracted status in the References list and also include a citation and full reference for the retraction notice.

Additional Editor Comments:

Thank you for sending in the revised version of your manuscript, “The consumption of dietary supplements among active individuals in Saudi Arabia” (Manuscript ID: PONE-D-25-22005R3). I appreciate the time and care you’ve put into addressing the previous editorial comments.

After reviewing your latest submission and response letter, I’m glad to see the manuscript has improved even further and is now very close to meeting the journal’s publication standards. Your updates to the measurement model and SEM reporting directly address the earlier concerns, and the overall writing is clearer and more consistent.

Before I can give a final acceptance, there are just a few small issues to sort out to make sure everything meets PLOS ONE’s policies and to avoid any last-minute delays. Most importantly, please double-check that your Data Availability statement in the manuscript matches what you’ve selected in the submission system. Right now, the manuscript says an anonymized dataset is available in the Supporting Information, but the submission metadata suggests there may be restrictions—these need to be consistent. If the data are fully available, please say so clearly in both places and indicate there are no restrictions. If there are restrictions, please explain the ethical or legal reasons, say who can be contacted for data access, and clarify exactly what can be shared and under what circumstances.

Also, please do a final check to make sure that ethics approval numbers, funding information, institutional names, and any reference codes are listed the same way throughout your manuscript and submission materials. Although the writing is much improved, it’s a good idea to do one last careful edit for typos, consistent use of terms and abbreviations, and to make sure all supplementary files are correctly named, uploaded, and referenced in the text.

With these final points in mind, the editorial decision is Minor Revision. Please send your revised manuscript along with a short point-by-point response explaining how you addressed each issue.

[Note: HTML markup is below. Please do not edit.]

[NOTE: If reviewer comments were submitted as an attachment file, they will be attached to this email and accessible via the submission site. Please log into your account, locate the manuscript record, and check for the action link "View Attachments". If this link does not appear, there are no attachment files.]

To ensure your figures meet our technical requirements, please review our figure guidelines: https://journals.plos.org/plosone/s/figures

You may also use PLOS’s free figure tool, NAAS, to help you prepare publication quality figures: https://journals.plos.org/plosone/s/figures#loc-tools-for-figure-preparation.

NAAS will assess whether your figures meet our technical requirements by comparing each figure against our figure specifications.

# Author comment: Author response to Decision Letter 4

**DOI:** 10.1371/journal.pone.0351208.r008

15 Apr 2026

Dear PLOS ONE Editors,

Thank you for the opportunity to revise and resubmit my manuscript to your esteemed journal. I have carefully addressed the editorial comments and updated the submission accordingly. The following revisions have been completed:

1- Addressed all editorial comments thoroughly.

2- Submitted a revised version of the manuscript.

3- Submitted a revised version of the manuscript with tracked changes.

4- We clarify that the anonymized data is available in the supporting files, as the online submission portal no longer permits manual updates to the Data Availability section.

I sincerely appreciate your time and consideration, and I look forward to your valuable feedback.

Attachment Submitted filename: Response to Reviewers 5.docx

# Editor report: Decision Letter 4

**DOI:** 10.1371/journal.pone.0351208.r009

**Authors:** Jordan Llego

16 Apr 2026

PONE-D-25-22005R4

Dietary supplement consumption among active individuals in Saudi Arabia

PLOS One

Dear Dr. Alsayegh,

Thank you for submitting your manuscript to PLOS ONE. After careful consideration, we feel that it has merit but does not fully meet PLOS ONE’s publication criteria as it currently stands. Therefore, we invite you to submit a revised version of the manuscript that addresses the points raised during the review process.

Please submit your revised manuscript by May 31 2026 11:59PM. If you will need more time than this to complete your revisions, please reply to this message or contact the journal office at plosone@plos.org. When you're ready to submit your revision, log on to https://www.editorialmanager.com/pone/ and select the 'Submissions Needing Revision' folder to locate your manuscript file.

Please include the following items when submitting your revised manuscript:

- A letter that responds to each point raised by the academic editor and reviewer(s). You should upload this letter as a separate file labeled 'Response to Reviewers'.
- A marked-up copy of your manuscript that highlights changes made to the original version. You should upload this as a separate file labeled 'Revised Manuscript with Track Changes'.
- An unmarked version of your revised paper without tracked changes. You should upload this as a separate file labeled 'Manuscript'.

If you would like to make changes to your financial disclosure, please include your updated statement in your cover letter. Guidelines for resubmitting your figure files are available below the reviewer comments at the end of this letter.

If applicable, we recommend that you deposit your laboratory protocols in protocols.io to enhance the reproducibility of your results. Protocols.io assigns your protocol its own identifier (DOI) so that it can be cited independently in the future. For instructions see: https://journals.plos.org/plosone/s/submission-guidelines#loc-laboratory-protocols. Additionally, PLOS ONE offers an option for publishing peer-reviewed Lab Protocol articles, which describe protocols hosted on protocols.io. Read more information on sharing protocols at https://plos.org/protocols?utm_medium=editorial-email&utm_source=authorletters&utm_campaign=protocols.

As the corresponding author, your ORCID iD is verified in the submission system and will appear in the published article. PLOS supports the use of ORCID, and we encourage all coauthors to register for an ORCID iD and use it as well. Please encourage your coauthors to verify their ORCID iD within the submission system before final acceptance, as unverified ORCID iDs will not appear in the published article. *Only*   the individual author can complete the verification step; PLOS staff *cannot*   verify ORCID iDs on behalf of authors.

We look forward to receiving your revised manuscript.

Kind regards,

Jordan Llego, PhD ELM, D. Hon. Ex., PhDN, RN

Academic Editor

PLOS One

Journal Requirements:

If the reviewer comments include a recommendation to cite specific previously published works, please review and evaluate these publications to determine whether they are relevant and should be cited. There is no requirement to cite these works unless the editor has indicated otherwise.

Please review your reference list to ensure that it is complete and correct. If you have cited papers that have been retracted, please include the rationale for doing so in the manuscript text, or remove these references and replace them with relevant current references. Any changes to the reference list should be mentioned in the rebuttal letter that accompanies your revised manuscript. If you need to cite a retracted article, indicate the article’s retracted status in the References list and also include a citation and full reference for the retraction notice.

Additional Editor Comments:

Thank you for submitting the fourth revised version of the manuscript entitled “Dietary supplement consumption among active individuals in Saudi Arabia.” The manuscript demonstrates improvement and continues to address a relevant public health topic with a substantial national sample. However, following editorial assessment, the manuscript is not yet ready for acceptance in its current form. A further round of major revision is required before the paper can be reconsidered for publication.

The primary reason for this decision is the presence of unresolved issues regarding internal consistency. Specifically, the manuscript does not present a coherent interpretation of the role of demographic factors. In the abstract, demographic factors are reported as not exhibiting a significant effect. In the Results section, several demographic variables in the bivariate analysis are reported as statistically significant. In the SEM section, the demographic domain is described as non-significant. In the Conclusion, sociodemographic factors are again described as significantly associated with dietary supplement consumption. These statements are not interchangeable and should not be presented together without clear explanation. It is essential to distinguish between variables significant in the bivariate analysis and those remaining significant in the structural model, and this distinction must be maintained consistently across the Abstract, Results, Discussion, and Conclusion. Address this concern with precision, ensuring that the manuscript reflects only what the analyses support.

A second major concern involves the description and interpretation of the measurement and modeling procedures. In the abstract, the questionnaire is described as a self-developed Likert-scale instrument, while in the Methods section it is characterized as adapted from validated local and international tools with minor modifications. This inconsistency should be resolved to ensure a single, accurate, and consistent description of the instrument. Furthermore, the manuscript states that good model fit is indicated by RMSEA <0.05, CFI ≥0.92, and a non-significant chi-squared statistic, yet the reported SEM results do not fully meet these criteria. Additionally, the manuscript claims that construct validity was supported by CFA, although the reported fit indices are only modest rather than unequivocally strong. These sections require revision to provide a more precise and methodologically defensible interpretation of the model fit and validation results.

The manuscript also requires a more rigorous final editorial review. Visible formatting and presentation issues persist, including unresolved currency notation and wording that indicate the manuscript has not undergone a thorough final proofread. Although these issues may seem minor compared to the conceptual concerns, they affect the overall credibility and polish of the submission. Terminology, symbols, table labels, and sentence-level wording should be fully standardized throughout the revised manuscript. I strongly encourage you to respond to each remaining issue directly and precisely. A persuasive revision will not simply say that concerns have been addressed; it must show exactly how the interpretation has been corrected, where the text has been revised, and why the final wording now matches the evidence presented. In particular, the revised manuscript must present a coherent analytical story from the abstract to the conclusion, especially with respect to the demographic findings and the interpretation of the SEM results.

[Note: HTML markup is below. Please do not edit.]

[NOTE: If reviewer comments were submitted as an attachment file, they will be attached to this email and accessible via the submission site. Please log into your account, locate the manuscript record, and check for the action link "View Attachments". If this link does not appear, there are no attachment files.]

To ensure your figures meet our technical requirements, please review our figure guidelines: https://journals.plos.org/plosone/s/figures

You may also use PLOS’s free figure tool, NAAS, to help you prepare publication quality figures: https://journals.plos.org/plosone/s/figures#loc-tools-for-figure-preparation.

NAAS will assess whether your figures meet our technical requirements by comparing each figure against our figure specifications.

# Author comment: Author response to Decision Letter 5

**DOI:** 10.1371/journal.pone.0351208.r010

22 May 2026

Editor's Comment 1 - Internal consistency of demographic findings: The manuscript does not present a coherent interpretation of the role of demographic factors. In the abstract, demographic factors are reported as not exhibiting a significant effect. In the Results section, several demographic variables in the bivariate analysis are reported as statistically significant. In the SEM section, the demographic domain is described as non-significant. In the Conclusion, sociodemographic factors are again described as significantly associated with dietary supplement consumption. These statements are not interchangeable and should not be presented together without clear explanation. It is essential to distinguish between variables significant in the bivariate analysis and those remaining significant in the structural model, and this distinction must be maintained consistently across the Abstract, Results, Discussion, and Conclusion.

Author Response: We thank the Editor for identifying this critical inconsistency. We fully agree that the distinction between bivariate and structural findings must be explicitly and consistently maintained throughout the manuscript. We have made the following targeted revisions:

(a) Abstract - Results: The phrase "demographic factors did not exhibit a significant effect" has been replaced with: "the demographic domain did not show a significant association in the structural equation model, although several demographic variables were significantly associated with supplement use in bivariate analysis." This wording now accurately reflects the layered nature of the findings.

(b) Conclusion: The statement "Sociodemographic factors - including gender, employment status, income level, and education - were significantly associated with dietary supplement consumption" has been revised to: "In bivariate analysis, sociodemographic factors - including gender, employment status, income level, and education - were significantly associated with dietary supplement consumption; however, the demographic domain did not retain significance in the structural equation model." This ensures readers clearly understand the level of analysis at which each association was observed.

The Results and Discussion sections already correctly describe both the bivariate (Table 1) and SEM findings separately, and these sections have been reviewed to confirm they remain consistent with the above changes.

Editor's Comment 2 - Inconsistent description of the questionnaire instrument: In the abstract, the questionnaire is described as a self-developed Likert-scale instrument, while in the Methods section it is characterized as adapted from validated local and international tools with minor modifications. This inconsistency should be resolved to ensure a single, accurate, and consistent description of the instrument.

Author Response: We apologize for this inconsistency. The Methods section accurately describes the instrument as adapted from validated local and international tools with minor modifications to ensure cultural relevance. The Abstract has been corrected accordingly. The phrase "self-developed Likert-scale questionnaire" has been replaced with "Likert-scale questionnaire adapted from validated local and international tools with minor modifications," bringing the Abstract into full alignment with the Methods section.

Editor's Comment 3 - SEM model fit criteria and interpretation: The manuscript states that good model fit is indicated by RMSEA <0.05, CFI >=0.92, and a non-significant chi-squared statistic, yet the reported SEM results do not fully meet these criteria. Additionally, the manuscript claims that construct validity was supported by CFA, although the reported fit indices are only modest rather than unequivocally strong.

Author Response: We thank the Editor for this important methodological observation. The reported SEM indices (RMSEA = 0.070, CFI = 0.953, SRMR = 0.082) do not meet the stricter thresholds of RMSEA <0.05 and CFI >=0.92 stated in the original Statistical Analysis section. We have therefore revised the stated thresholds to those that the model actually satisfies: RMSEA <0.08 and CFI >=0.90, which are widely accepted as indicators of acceptable fit in structural equation modeling (Hu & Bentler, 1999; Browne & Cudeck, 1993). The phrase regarding chi-squared non-significance has also been revised, as a significant chi-squared statistic is expected with large samples and is not informative as a standalone criterion.

Regarding construct validity, the CFA fit indices (CFI = 0.901, RMSEA = 0.072) support acceptable rather than strong construct validity. The relevant text has been reviewed to ensure the claim is appropriately qualified and does not overstate the evidence.

Editor's Comment 4 - Currency notation and formatting issues: Visible formatting and presentation issues persist, including unresolved currency notation and wording that indicate the manuscript has not undergone a thorough final proofread. Terminology, symbols, table labels, and sentence-level wording should be fully standardized throughout the revised manuscript.

Author Response: We sincerely apologize for these presentation issues. A thorough editorial review has been conducted across the full manuscript. Specifically:

(a) Currency notation: All instances of the unresolved Saudi Riyal symbol - appearing as an embedded image or unrendered character - have been replaced uniformly with the standard abbreviation "SAR" throughout the manuscript, including the Methods, Results, Tables, and all figure/supplementary captions.

(b) General proofreading: Sentence-level wording, table labels, and terminology have been reviewed and standardized throughout the manuscript. We are confident the revised manuscript now meets the journal presentation standards.

We hope that the revised manuscript now satisfactorily addresses all concerns raised. We remain available to provide any further clarification required.

Attachment Submitted filename: Response to Reviewers 5-26.docx

# Editor report: Decision Letter 5

**DOI:** 10.1371/journal.pone.0351208.r011

**Authors:** Jordan Llego

25 May 2026

Dietary supplement consumption among active individuals in Saudi Arabia

PONE-D-25-22005R5

Dear Dr. Alsayegh,

We’re pleased to inform you that your manuscript has been judged scientifically suitable for publication and will be formally accepted for publication once it meets all outstanding technical requirements.

Within one week, you’ll receive an e-mail detailing the required amendments. When these have been addressed, you’ll receive a formal acceptance letter and your manuscript will be scheduled for publication.

An invoice will be generated when your article is formally accepted. Please note, if your institution has a publishing partnership with PLOS and your article meets the relevant criteria, all or part of your publication costs will be covered. Please make sure your user information is up-to-date by logging into Editorial Manager at Editorial Manager® and clicking the ‘Update My Information' link at the top of the page. For questions related to billing, please contact billing support.

If your institution or institutions have a press office, please notify them about your upcoming paper to help maximize its impact. If they’ll be preparing press materials, please inform our press team as soon as possible -- no later than 48 hours after receiving the formal acceptance. Your manuscript will remain under strict press embargo until 2 pm Eastern Time on the date of publication. For more information, please contact onepress@plos.org.

Kind regards,

Jordan Llego, PhD ELM, D. Hon. Ex., PhDN, RN

Academic Editor

PLOS One

Additional Editor Comments (optional):

Thank you for submitting the revised version of your manuscript entitled “Dietary supplement consumption among active individuals in Saudi Arabia”.

I am pleased to inform you that the manuscript is now accepted for publication. The revised version has adequately addressed the previously raised concerns, particularly the clarification of demographic findings, the consistent description of the questionnaire, and the more appropriate interpretation of the SEM results.

Only routine editorial and proofreading corrections may be made during the production stage to ensure consistency in formatting, terminology, symbols, and sentence-level presentation.

Congratulations to you and your co-authors.

Reviewers' comments:

# Editor report: Acceptance letter

**DOI:** 10.1371/journal.pone.0351208.r012

**Authors:** Jordan Llego

PONE-D-25-22005R5

PLOS One

Dear Dr. Alsayegh,

I'm pleased to inform you that your manuscript has been deemed suitable for publication in PLOS One. Congratulations! Your manuscript is now being handed over to our production team.

At this stage, our production department will prepare your paper for publication. This includes ensuring the following:

* All references, tables, and figures are properly cited

* All relevant supporting information is included in the manuscript submission,

* There are no issues that prevent the paper from being properly typeset

You will receive further instructions from the production team, including instructions on how to review your proof when it is ready. Please keep in mind that we are working through a large volume of accepted articles, so please give us a few days to review your paper and let you know the next and final steps.

Lastly, if your institution or institutions have a press office, please let them know about your upcoming paper now to help maximize its impact. If they'll be preparing press materials, please inform our press team within the next 48 hours. Your manuscript will remain under strict press embargo until 2 pm Eastern Time on the date of publication. For more information, please contact onepress@plos.org.

You will receive an invoice from PLOS for your publication fee after your manuscript has reached the completed accept phase. If you receive an email requesting payment before acceptance or for any other service, this may be a phishing scheme. Learn how to identify phishing emails and protect your accounts at https://explore.plos.org/phishing.

If we can help with anything else, please email us at customercare@plos.org.

Thank you for submitting your work to PLOS ONE and supporting open access.

Kind regards,

PLOS ONE Editorial Office Staff

on behalf of

Dr. Jordan Llego

Academic Editor

PLOS One
